# Macrocyclic bis-diphosphenes demonstrating bimetallic *exo*- and *endo*-cyclic binding modes†

**DOI:** 10.1039/d4sc03516j

**Published:** 2024-07-18

**Authors:** Lisa N. Kreimer, Terrance J. Hadlington

**Affiliations:** a Fakultät für Chemie, Technische Universität München Lichtenberg Strasse 4 85747 Garching Germany terrance.hadlington@tum.de

## Abstract

Macrocyclic bis-diphosphenes, formally heavier derivatives of macrocyclic azobenzenes, are accessed for the first time. These are synthesised in a reproducible fashion, through the nickel-mediated homocoupling of xanthene-derived NHC-stabilised bis-phosphinidene units. This gives direct access to target macrocyclic bis-diphosphenes 2, featuring *exo*-cyclic coordinated Ni^0^ fragments. The *endo*-cyclic binding mode in 3 is realised by NHC-abstraction using CuCl, so demonstrating two homometallic binding modes for this system. Additionally, reaction with CuCl in acetonitrile yields small amounts of a tetra-metallic Ni^II^/Cu^I^ complex, which establishes simultaneous *exo*- and *endo*-cyclic metal binding. Fluctional solution state behavior in these systems is explored through variable temperature NMR spectroscopy, in addition to computational bonding analyses, giving the first insights into this novel class of compounds.

## Abstract 2

Macrocyclic bis-diphosphenes, formally heavier derivatives of macrocyclic azobenzenes, are accessed for the first time.

## Graphical abstract

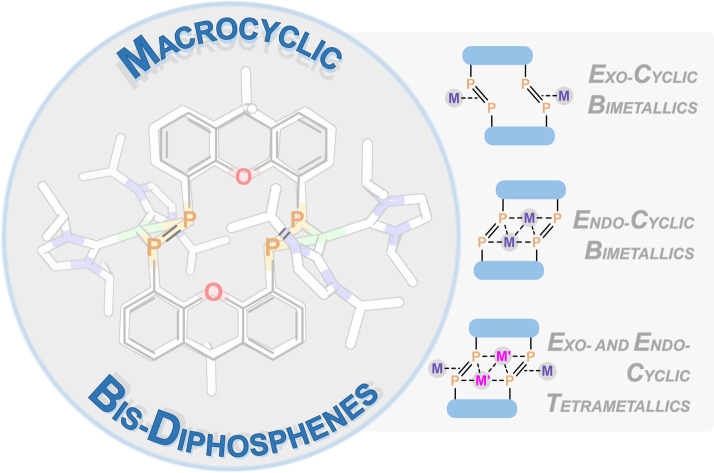

## Introduction

**Fig. 1:**
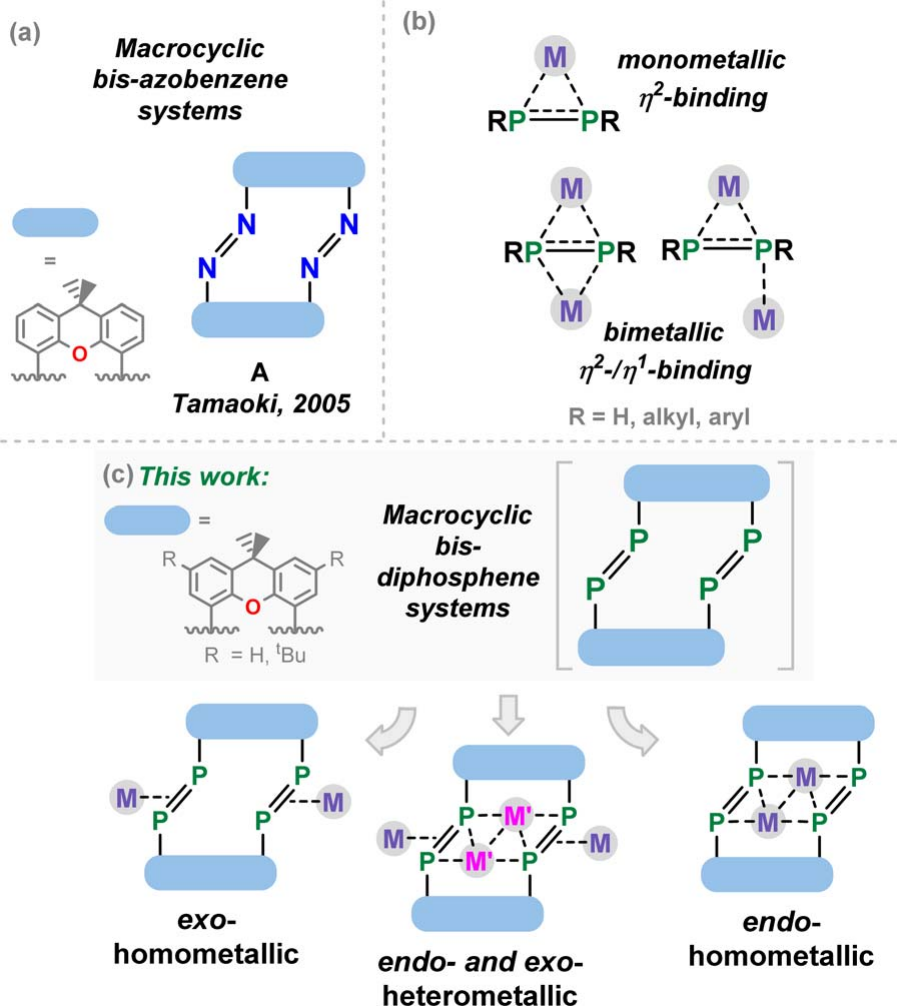
(a) An example of a xanthene-derived macrocyclic bis-azobenzene system; (b) mono- and bi-metallic binding modes of diphosphenes; (c) binding modes of macrocyclic bis-diphosphene systems described in this work.

The discovery of crown-ethers by Pedersen 55 years ago stands as the starting point for synthetic macrocyclic chemistry,^1,2^ laying the foundations for the broader fields of host-guest and supramolecular chemistry.^3,4^ Since that time, numerous classes of macrocyclic compounds have been developed, particularly finding applications as molecular sensors,^5^ mechanically interlocked supramolecules,^6,7^ and switchable systems. This latter class of compound has benefited from azobenzene photo-switches,^8^ leading to, for example, the development of the xanthene-derived azobenzene photoswitch A from Tamaoki and co-workers in 2005 (Fig. 1(a)).^9^ Since that time, significant efforts have gone towards the synthesis of azobenzene-containing macromolecules, essentially defining a new subfield in supramolecular chemistry.^10^ In addition to those nitrogen-containing macrocycles and their saturated derivatives (*e.g.* aza-crown ethers^11^), considerable work has demonstrated that phosphine-containing macrocycles are an accessible class of macrocyclic support.^12,13^ It has also been demonstrated that ‘free’ diphosphenes, as well as their metal complexes, undergo both thermal and visible-light mediated conformational switching.^14–19^ Combining these concepts, one may consider heavier macrocyclic azobenzene derivatives: the macrocyclic diphosphenes. The coordination chemistry of diphosphenes is arguably richer than that of the lighter nitrogen congeners, possibly owing to the perturbed π-bonding in heavier p-block dimers allowing for more favourable complexation due to a greater charge localisation at the P-centres.^20^ Indeed, a range of binding modes are known for discreet diphosphene moieties, including monometallic η^2^-,^21–23^ bimetallic η^2^-,^24,25^ and mixed bimetallic η^2^/η^1^-binding (Fig. 1(b)).^26^ We therefore hypothesized that macrocyclic diphosphene systems may be powerful scaffolds for access to discreet multi-metallic complexes (Fig. 1(c)). Though bis-diphosphenes are known,^27–30^ a controlled synthetic protocol for access to such macrocyclic derivatives has prevented a deeper exploration of their coordination chemistry, particularly relative to that for well-established nitrogen congeners.

In this contribution, we describe our efforts towards developing such a synthetic route, in the reproducible Ni-meditated phosphinidene coupling of xanthene-derived NHC-stabilised bis-phosphinidenes (NHC-PPs).‡‡We refer to these species as NHC-stabilised bis-phophinidenes given that the chemistry described herein utilises these systems as phosphinidene transfer reagents. We are aware that these systems could equally be described as inversely-polarised bis-phosphaalkenes. These reactions give access to macrocyclic bis-diphosphenes, bearing a central 14-membered ring featuring two P=P linkages. These macrocycles demonstrate both *exo*- and *endo*-cyclic binding of Ni^0^, in addition to simultaneous *exo*- and *endo*-cyclic binding of Ni and Cu, respectively. Solid- and solution-state behaviour of these species, in conjunction with DFT calculations, gives the first insights into bonding aspects of this novel class of macrocycles.

## Results and discussion

**Scheme 1:**
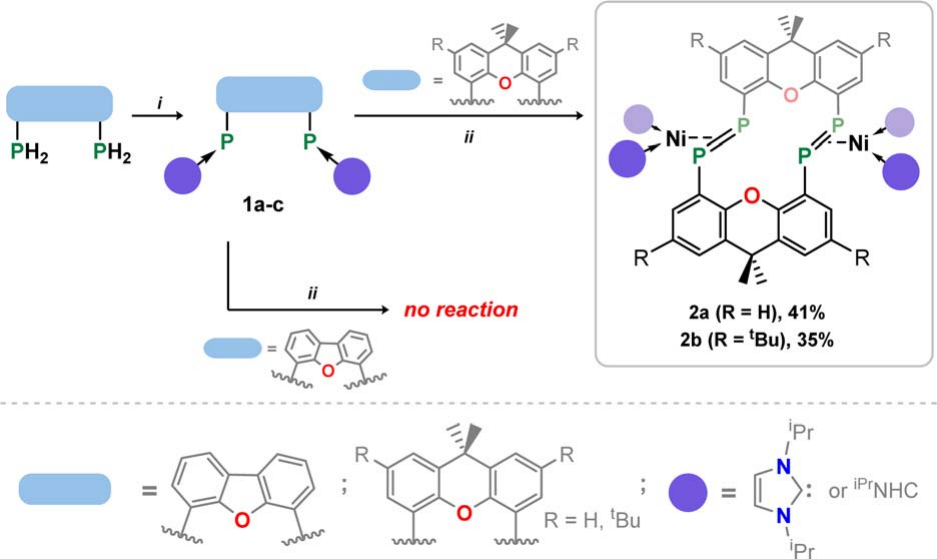
(i) 4 equiv. ^iPr^NHC, 100 °C, 2 days (1b) or 4 day (1c), ^iPr^NHCH_2_; (ii) 2 equiv. [Ni(cod)_2_], 100 °C, 4–6 days, 2 COD.

**Fig. 2:**
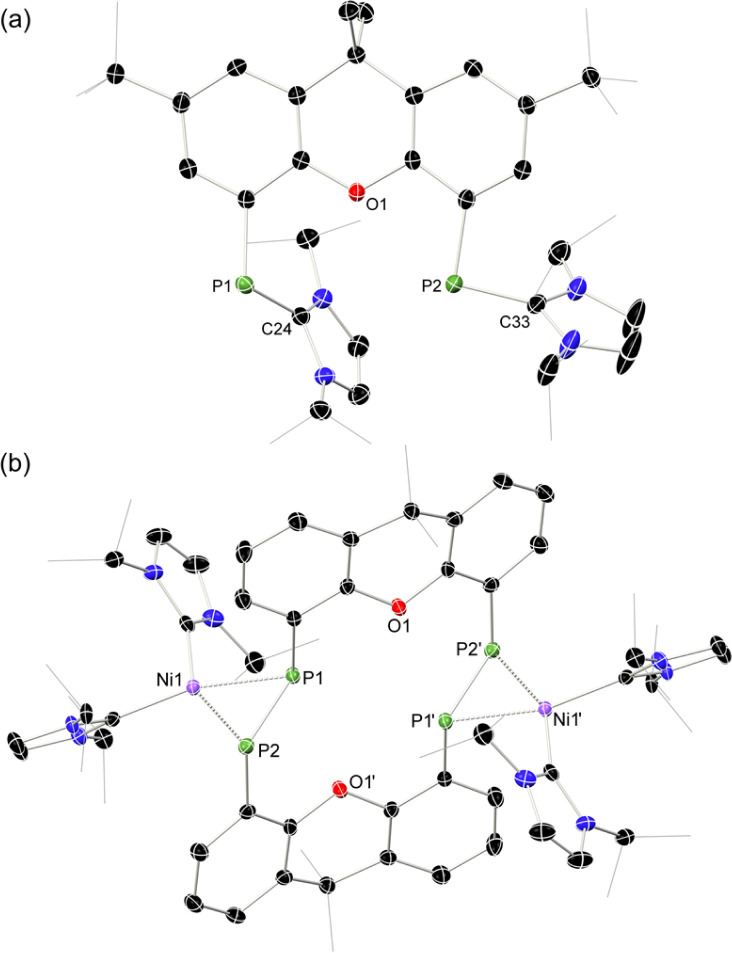
Molecular structures of (a) 1b, and (b) 2a, with thermal ellipsoids at 30% probability, and hydrogen atoms omitted for clarity. Selected bond lengths (Å) and angles (°) for 1b: P1–C24 1.779(4); P2–C33 1.809(4); C2–P1–C24 106.6(2); C12–P2–C33 96.2(2). For 2a: P1–P2 2.147(1); P1–Ni1 2.260(1); P2–Ni1 2.251(1); P1⋯P1′ 3.542(2); C2–P2–P1 105.3(1); C12–P1–P2 99.0(1); P1–Ni1–P2 56.83(4).

Our starting point for access to macrocyclic diphosphene systems drew inspiration from tetrylene- and phosphinidene-coupling chemistry. Inoue *et al.* showed in 2012 the NHC-stabilised (silyl)(hydrido)silylene [^Me^NHC·Si(H)(Si^*t*^Bu_3_)] reacts with [Ni(COD)_2_] in forming the bis(NHC) nickel-disilene complex (^Me^NHC)_2_Ni·[η^2^{Si(H)(Si^*t*^Bu_3_)}_2_].^31^ Similar chemistry was later demonstrated for related bis(aryl)stannylene species, by the group of Wesemann.^32^ More appropriate to the current work, Radius *et al.* reported that NHC-stabilised phenyl-phosphinidene also reacts with [Ni(COD)_2_] to form the diphosphene complex [(NHC)_2_Ni·{η^2^-(P_2_Ph_2_)}].^33^ Therefore, we aimed to utilize NHC-PPs as building blocks for the targeted macrocyclic species. For this we employed our earlier reported 4,5-bis(phosphinidene)xanthene 1a,^34^ and additionally developed two further examples, namely the 2,7-di-*tert*-butylxanthene derivative 1b, and 4,6-bis(phosphinidene)dibenzofuran system 1c. Notably, NHC-PP 1b has a considerably improved solubility over 1a, whilst 1c has a wider bite-angle (*i.e. d*_P⋯P_: for 1b: 4.435(2) Å; for 1c: 5.674(3) Å), similar to differences observed for related bis-phosphine ligands,^35,36^ which is expected to affect PP-coupling chemistry. These two novel examples of NHC-PPs were accessed *via* the same route as used for 1a, that is through carbene reduction of the bis-phosphine systems (Scheme 1). Both could be crystallised, as deep orange (1b) or red (1c) solids (see Fig. 2(a) and S40 in ESI†), with characteristic ^31^P NMR shifts at −72.8 (1b) and −86.0 (1c) ppm.

The PP-coupling reaction was carried out through the addition of 1 equiv. [Ni(COD)_2_] to 1a–1c in toluene, followed by heating to 100 °C. For dibenzofuran system 1c no coupling reaction was observed, and only the NHC-PP starting material was recovered, even after heating the mixture for 1 week. Conversely, heating of reaction mixtures involving 1a or 1b for 4 and 6 days, respectively, led to the gradual deposition of large, deep red crystals. Structural analysis of these samples revealed that targeted macrocyclic bis-diphosphene complexes 2a and 2b are indeed formed (Fig. 2 and S41 in ESI†). Optimisation of reaction conditions was key in maximizing the yield of these species: 100 mg mL^−1^ of 1a or 1b in toluene was found to be the ideal concentration. Heating above 100 °C negatively affects yields, and lower temperatures led to considerably longer reaction times. Optimised conditions allow for a reproducible yield of both systems of between 35 and 40%. That 1c did not lead to a PP-coupling reaction may suggest that pre-chelation of Ni^0^ plays a role in this process, which gives some evidence that formation of 2a and 2b is a templated synthesis, known to be an important factor more broadly in macrocycle synthesis.^37^ This is further supported by the ready formation of 1 : 1 chelation complexes of both 1a and 1b towards [GeCl]^+^ upon addition of these ligands to [GeCl_2_·dioxane],§§For 1a this species can be found in ref. 34. For 1b, this details for the synthesis and characterisation of this compound can be found in the ESI, as 1b·GeCl. a reaction which apparently does not proceed with 1c.

**Fig. 3:**
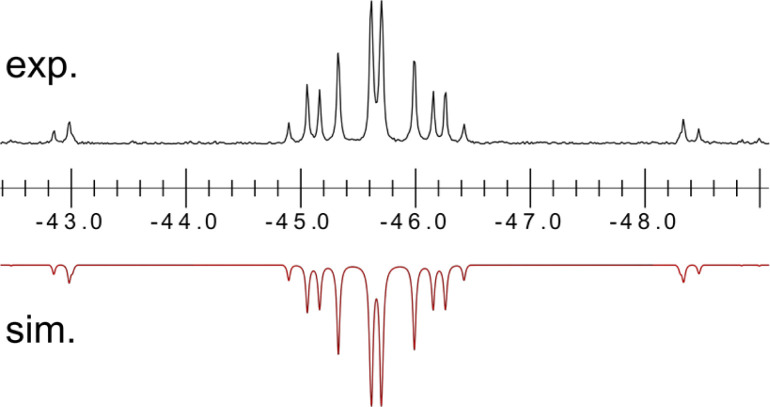
Experimental (above) and simulated (below) ^31^P{^1^H} NMR spectrum for 2b at ambient temperature in d_8_-THF.

**Fig. 4:**
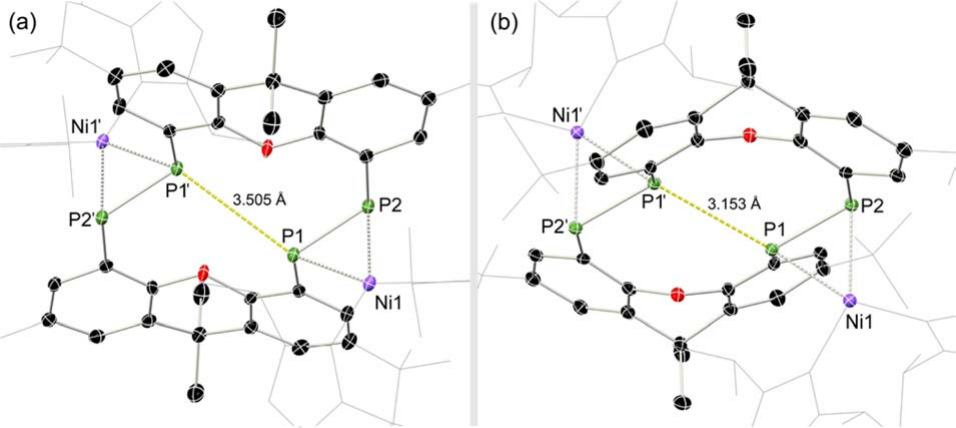
Molecular structures of polymorphs (a) 2b and (b) 2b′, indicating distinct changes in cross-ring P–P distance.

Once crystallised, both macrocyclic species 2a and 2b are poorly soluble in aliphatic solvents. 2a is only slightly soluble in THF, with the ^*t*^Bu-substituents in 2b improving this to some degree. Both species feature two xanthene scaffolds linked by diphosphene fragments, in a *trans*,*trans*-conformation, and forming a central 14-membered ring. Each [PP] fragment is η^2^-bound by a [Ni(NHC)_2_] fragment perpendicular to the xanthene planes, and in the plane of the central [P_4_] unit. The molecular structures of both 2a and 2b are inversion symmetric, crystallising in the spacegroup *P*2_1_/*c*, giving two identical P–P bond distances in each species, of 2.145(1) Å (2a) and 2.147(1) Å (2b), which are between typical P–P double and single bonds.^38^ In addition, only slight *cis*-bending of the [P_2_] units is observed (C–P–P–C torsion in 2a = 5.4(1)°; in 2b = 8.8(1)°), suggesting a high degree of π-complex character in the [NiP_2_] moieties in these complexes. In both systems the two diphosphene fragments do not lie perpendicular to the xanthene planes, with this twisting leading to short cross-ring P1⋯P1′ distances (2a: 3.542(2) Å; 2b: 3.505(1) Å), which sit just within the sum of the van der Waals radii (3.6 Å).^39^ Despite the highly symmetric solid-state structure of 2b, a complex multiplet is observed in the solution-state ^31^P{^1^H} NMR spectrum of this species at room temperature (Fig. 3), which we presume arises from through-space coupling of P1 and P1′.¶¶We note that this coupling is not observed for 2a, possibly due to a longer cross-ring P⋯P bond. We hypothesise that the ^*t*^Bu groups in 2b lower the energy of xanthene flexing, through steric repulsion between those groups and Ni-coordinated NHC ligands. This is somewhat confirmed through the simulation of this multiplet, which gives large through-space ^1^*J*_P⋯P_ coupling value of 180 Hz.||||The simulation was modelled using gNMR. This value, however, is rather large when considering the observed P1⋯P1′ distance in the solid state structure of 2b (3.505(1) Å), where literature known systems with such a coupling are expected to have a P⋯P distance of ∼3.0–3.2 Å.^40–42^ We thus attempted to crystallise 2b under varying conditions, and found that THF/pentane solutions of this species led to the crystallization of an additional polymorph at room temperature (*i.e.*2b′) which contains a markedly shorter P1⋯P1′ distance of 3.153(2) Å, now correlating well with known systems demonstrating a large through-space PP-coupling constant. This shorter contact in polymorph 2b′ is apparently made viable through bending of the xanthene backbone (Fig. 4), also bringing one aryl group of this backbone (*viz* C8–C13) into coplanarity with the PP bonds, perhaps leading to favourable π-orbital overlap.

**Fig. 5:**
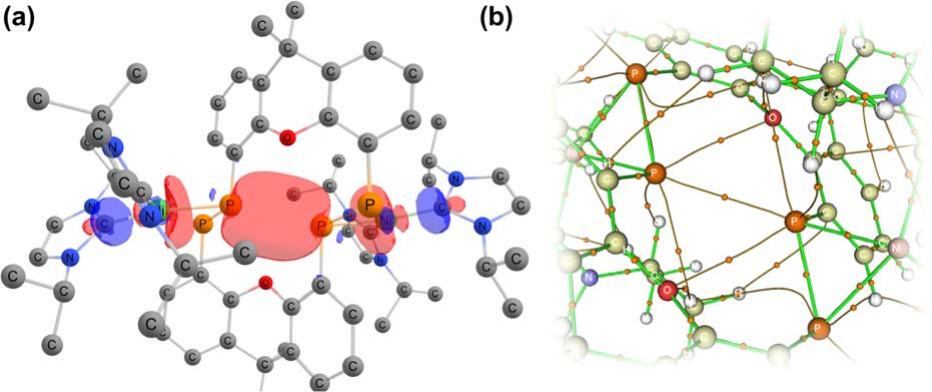
(a) Combination of orbitals leading to a cross-ring P–P interaction, found through a second-order perturbation theory anaylsis of 2; (b) a QTAIMs analysis of 2 indicating a bond critical point between P1 and P1′.

Further investigating this phenomenon using VT NMR ^31^P{^1^H} NMR spectroscopy in the range from −80 to 60 °C yielded surprising results (Table 1). The multiplet features of the described RT ^31^P{^1^H} NMR spectrum of 2b coalesce to a relatively sharp singlet at −80 °C (Fig. S28†), suggesting that no short P1⋯P1′ interaction is present at this temperature. On warming, this resonance broadens and splits into a multiplet at −40 °C, becoming more resolved with increasing temperature. The *J*-coupling parameters within this multiplet can be determined by simulating each spectrum (Table 1): this reveals a gradual increase in the observed through-space ^1^*J*(*P*_A_⋯*P*_A′_), up to a maximum of 188.5 Hz at 60 °C, and a minimum of 174.3 Hz at −40 °C. This would suggest that an increase in temperature leads to a shorter P1⋯P1′ contact; we hypothesise that this energy is required to affect xanthene flexing, allowing for the through-space contact *e.g.* as observed in polymorph 2b′. In addition to this observation, we note that all other PP-coupling constants increase dramatically at −40 °C (Table 1). This would suggest that all values tend towards parity at low temperatures, culminating in the observed singlet at −60 °C, relating to a symmetrical system in which the P1–P1′ bond distance is too great to allow for through-space coupling. This is in-keeping with molecular flexibility being of importance in allowing for the observed coupling at higher temperatures. A computational analysis of the bonding in 2b gives further information regarding this effect.**In all cases, the full structure of 2a was used, beginning from the coordinates for the solid-state structures of 2b and 2b′ (*i.e.* through removal of ^*t*^Bu). An initial optimisation was carried out at the B3LYP//def2SVP level of theory. A further single-point optimisation was carried using those geometries, at the B97D3//def2TZVPP level of theory, employing the SCF[THF] solvent model. Optimisation of polymorph 2b′ leads to a lowest energy conformation in which a further contraction of the through-space P1⋯P1′ distance is observed, from 3.153 to 3.082 Å. Analysing the frontier orbitals of this species, the HOMO (−3.166 eV) represents electron density at P1 and P1′, oriented towards each other (Fig. S44 in ESI†); this is known to be a key factor in systems which demonstrated a high through-space coupling constant, notably so in reported *peri*-substituted diphosphino-naphthalene,^41–43^ and tetraphosphine-ferrocenyl derivatives.^40^ The HOMO−1 (−3.417 eV) and −2 (−3.424 eV) are Ni-centred, whilst HOMO−3 (−3.614 eV) and −4 (−3.629 eV) represent a degree of electron density at P2/P2′. Finally, the HOMO−9 (−4.156 eV) formally represents a weak σ-bonding interaction between P-centres P1 and P1′. A deeper analysis using NBO and QTAIM methodologies further reinforces this picture (Fig. 5): the former reveals a weak but present cross-ring P–P bonding interaction, with small Wiberg and Mayer bond indices of 0.07. A second-order perturbation theory analysis reveals that lone-pairs on P1 and P1′ interact with the σ*-orbital of the opposing P–Ni bond (*e.g.* LP_P1_ → 
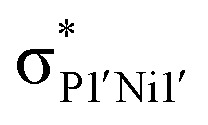
), with a total energy of 5.78 kcal mol^−1^, visualised in Fig. 5(a) and S44.† Whilst such an interaction has been noted as a contributor to through-space coupling in *e.g. peri*-substituted phosphorus–tellurium systems,^44^ the aforementioned lone-pair orientation likely plays a stronger role.^42,45^ Nevertheless, a QTAIMs analysis also shows a clear bond critical point between P1 and P1′ (Fig. 5(b)), in agreement with the described weak but present interaction.

**Table 1:** Simulated *J*-coupling parameters for variable-temperature ^31^P{^1^H} NMR spectra of 2b between −40 and 60 °C^a^

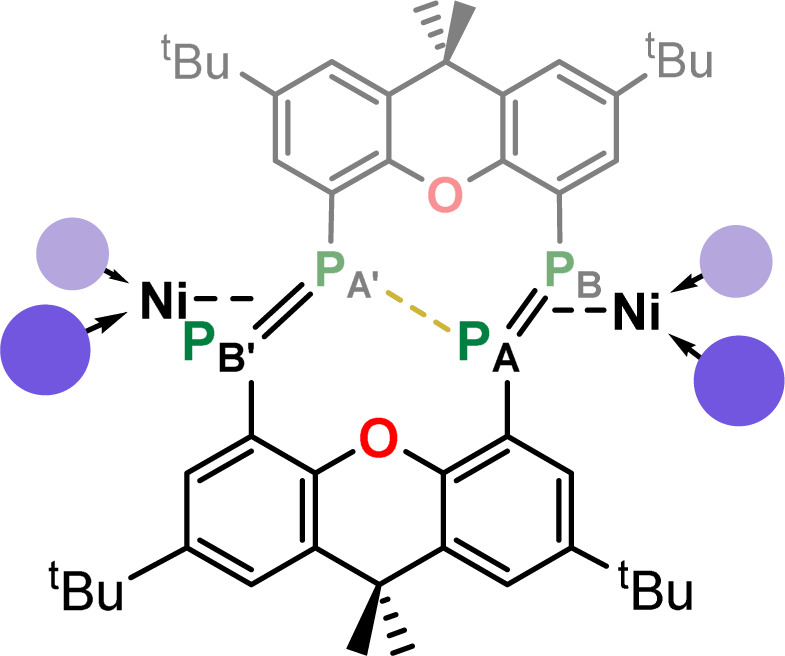						
Temp. [°C]	AB	A′B′	AA′	BB′	AB′	A′B
60	404.7	404.7	188.5	0.6	1.5	1.5
40	401.3	406.5	183.4	1.0	9.7	6.4
20	403.1	403.1	179.8	1.0	1.4	1.4
0	403.6	398.0	175.4	1.3	9.6	6.1
−40	372.2	368.2	174.3	65.2	55.3	45.2

a For solutions of 2b in D_8_-THF; all values given in Hz.

**Fig. 6:**
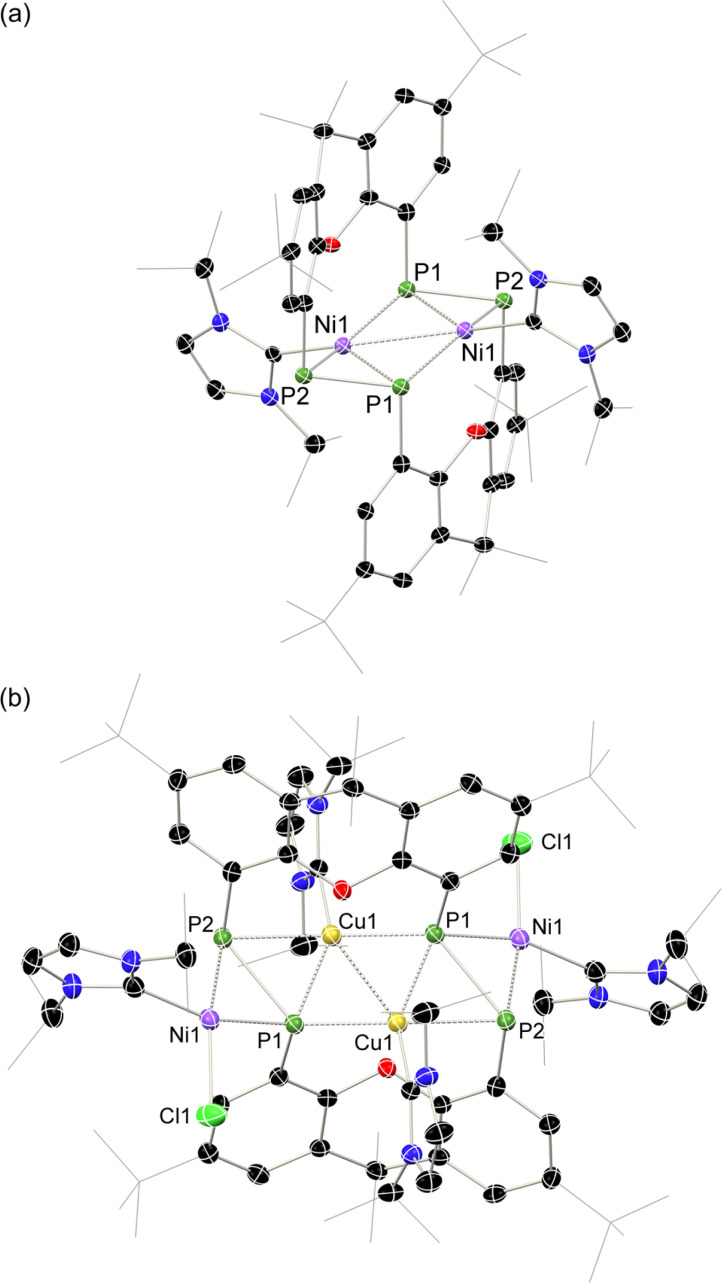
Molecular structures of polymorphs (a) 3 and (b) 4, indicating distinct changes in cross-ring P–P distance. Selected bond lengths (Å) and angles (°) for 3: P1–P2 2.154(2); P1–Ni1 2.359(2); P1–Ni1′ 2.278(2); P1–Ni1 2.229(2); Ni1–Ni1′ 2.745(1); C2–P1–P2 98.3(2); C12–P2–P1 97.5(2); C2–P1–Ni1 105.6(2); C12–P2–Ni1 100.9(2); Ni1–P2–P1 65.10(6). For 4: P1–P2 2.353(2); P1–Ni1 2.141(2); P2–Ni1 2.259(2); P1–Cu1 2.218(2); P1–Cu1′ 2.164(2); P2–Cu1 2.369(2); Cu1–Cu1′ 2.551(1); C2–P1–P2 105.3(2); C12–P2–P1 115.2(2).

Given the 14 membered macrocyclic nature of 2a and 2b, we were curious as to their potential host-guest chemistry. For these studies only 2b was utilized, due to its considerably more favourable solubility when compared with 2a. We aimed to explore the potential formation of hetero-metallic species, focusing on coinage metals given their rich coordination chemistry. It quickly became apparent that, although 2b itself is not particularly air sensitive, upon reaction with coinage metal species an extremely oxygen sensitive compound is formed. Conducting the reaction of CuCl with 2b under stringent exclusion of oxygen and moisture leads to the formation of deep green solutions. Initial analysis *via*^31^P{^1^H} NMR spectroscopy indicates the disappearance of the characteristic multiplet for 2b, and the appearance of two highly broadened resonances at *δ* = 129 and −81 ppm, indicative of two distinct coordination environments for P. Careful crystallisation of concentrated THF solutions at −30 °C led to the formation of extremely air sensitive dichroic green-red crystals. Single-crystal X-ray diffraction (SC-XRD) analysis revealed this product to be the *endo*-cyclic [Ni_2_] macrocycle complex 3, through NHC-abstraction from *exo*-cyclic 2b (Fig. 6(a)). At the core of 3 is a discreet [Ni_2_] unit, each Ni centre coordinated by three P-centres, and one NHC. This leads to the above observed differing P-environments, with P2 and P2′ being 4-coordinate, and P1 and P1′ being 3-coordinate. It is presumably the highly exposed nature of these P1/P1′ centres which renders 3 highly reactive towards oxidation. The P–P distances in 3 (*d*_P1P2_ = 2.154(2) Å) are similar to those observed in 2b (*d*_P1P2_ = 2.145(1) Å), as are the observed P–P torsion angles (C–P–P–C torsion = 7.3(2)°), again indicative of π-complex character. Given the long Ni⋯Ni distance of 2.745(1) Å, which is beyond what is expected for the sum of the covalent radii, only a very weak Ni–Ni bond is likely. This is corroborated by a computational NBO analysis, in which a Wiberg bond index of 0.07 is found. The ^31^P{^1^H} NMR spectrum of a d_8_-THF solution of 3 is in keeping with that observed in crude reaction mixtures, with two highly broadened doublet resonances at *δ* = 129.4 and −81.2 ppm (^1^*J*_PP_ = 447 Hz).††††The resolution of these peaks does not improve in cold solutions, as ascertained by VT NMR studies, and the complex seems to decompose at elevated temperatures. This *J*-coupling value aligns with those simulated for the diphosphene units in 2b (*i.e.* 403 Hz at 20 °C, Table 1). In addition, and again as per 2b, this value between reported values for unsymmetrical diphosphenes and diphosphines.^18,30,46–49^ A single xanthene environment is observed in the ^1^H NMR spectrum, however, which would indicate coordinate fluctionality in solution. THF solutions of 3 are deep green in colour, in contrast to orange 2a/b, showing absorptions in the visible region at 614 and 416 nm. A TD-DFT analysis of a reduced version of 3 (*viz*. 3′, Fig. S46 in ESI†) indicates numerous absorptions between 675 nm and 425 nm which contribute to a major absorption centred at ∼530 nm (Fig. S45†), shifted from that observed experimentally (*λ*_max_ = 614 nm). One main absorption here is found at 507 nm (*f* = 0.0397), comprising 6 transitions. These absorptions represent Ni → P or Ni/P → Ar charge transfer processes (Fig. S46 in ESI†), and are strongly delocalized within the [P_4_Ni_2_] unit, which may point towards delocalized orbitals within this central moiety.

**Scheme 2:**
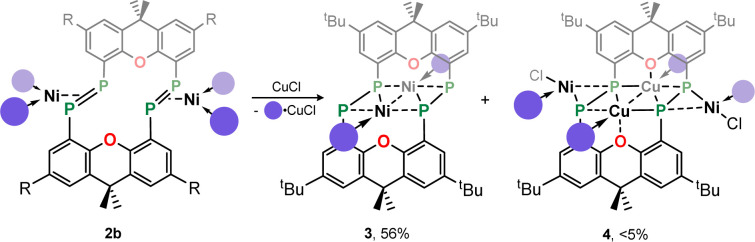
The synthesis of *endo*-cyclic [Ni]_2_ complex 3, and tetra-metallic 4.

Conducting the described reaction of 2b with CuCl in MeCN, without stirring, led to solutions of 3 in addition to the formation of a small amount of deep red block-like crystals, which contrasts to the green-red colouration of 3. A SC-XRD analysis revealed this complex to be 4 (Scheme 2, Fig. 6(b)), a hetero-tetrametallic complex in which both Ni and Cu bind the macrocyclic bis-diphosphene ligand. Specifically, two *endo*-cyclic [NHC·Cu] units and two *exo*-cyclic [NHC·NiCl] units bind the macrocyclic system, presumably through the formal oxidative addition of *in situ* generated NHC·CuCl to concomitantly formed 3.‡‡‡‡We note that refinement withe *exo*-cyclic Ni and *endo*-cyclic Cu yields considerably more favourable *R*-values, making this the most realistic metal distribution mode in this system. Only a very low yield of 4 is formed (<5%), which may suggest that such a process is only weakly favourable. As 2a/b and 3, the molecular structure of 4 has a mirror plane which dissects the P–P bonds, and as such each P–P unit is identical. This species bears P–P bonds ∼0.2 Å longer than the former two species, and is thus better described as having formal P–P single bonds, indeed longer than many diphosphines. Observing the bond distances between these diphosphine units (*i.e.* P1–P2) and the metal centres in this complex (*i.e.* Cu1, Cu1′, and Ni1), all such interactions are shorter when compared to those found in 2a/b and 3. This would therefore suggest formal covalent bonding character in complex 4, compared to what we describe as π-complex character in 2a/b and 3. As such, 4 is best described as containing formally Ni^II^ and Cu^I^ centres. Once crystallised, compound 4 has an extremely poor solubility in organic solvents, which, in conjunction with its low yield, precluded the acquisition of any data beyond its molecular structure. Nevertheless, its formation suggests that the class of macrocyclic ligand reported here may be a powerful scaffold for the development of hetero-polymetallic complexes.

## Conclusions

In summary, we have developed a synthetic protocol for access to macrocyclic bis-diphosphenes, *via* the nickel-mediated coupling of NHC-PPs. This reaction is dependent on the binding angle of the phosphindene precursors, with a wide bite angle failing to produce targeted macrocycles. Further, we have demonstrated both *exo*- and *endo*-cyclic homo-bimetallic binding of Ni^0^ in these systems, in addition to the hetero-tetrametallic binding of Ni^II^/Cu^I^, demonstrating the impressive coordination capacity of this ligand class. This stands as an initial entry into the chemistry of these heavier macrocyclic azobenzene analogues, which we continue to explore.

## Author contributions

L. N. K. carried out all experimental work and characterisation. T. J. H. designed and supervised the project, carried out computational work, and prepared the manuscript.

## Conflicts of interest

There are no conflicts to declare.

## Supplementary Material

SC-015-D4SC03516J-s001

SC-015-D4SC03516J-s002

## Data Availability

All data is available in the ESI† of this manuscript (synthetic and characterisation data, reprints of all spectra, crystallographic and computational details and coordinates), through the CCDC (X-ray data), or by contacting the authors.
