# Supplementary material for: Macrocyclic bis-diphosphenes demonstrating bimetallic exo- and endo-cyclic binding modes
Source: Chem Sci. 2024 Jul 18. Online ahead of print. doi: 10.1039/d4sc03516j (PMC11389489; doi:10.1039/d4sc03516j)
Supplement: SC-OLF-D4SC03516J-s001 [file SC-OLF-D4SC03516J-s001.pdf]

Electronic Supplementary Material (ESI) for Chemical Science.
This journal is © The Royal Society of Chemistry 2024

Supporting Information

Macrocyclic bis-diphosphenes demonstrating bimetallic *exo-* and *endo*-cyclic binding modes

Lisa N. Kreimer,[a] and Terrance J. Hadlington*,[a]

[a] Lehrstuhl für anorganische Chemie mit Schwerpunkt neue Materialien, Technische Universität München, Lichtenbergstraße 4, 85747 Garching

1. **Experimental methods and data……………………..S2**
   - General Considerations……………..…………**S2**
   - Synthetic details and data.……….……………**S2**
   - NMR, MS, IR, and UV/vis plots……..…..……..**S8**
2. **X-ray crystallographic details……………………..…S29**
3. **Computational methods and details……………..…S33**
4. **References……………………………………………...S42**

1. Experimental methods and data

General considerations. All experiments and manipulations were carried out under dry oxygen free argon atmosphere using standard Schlenk techniques or in a MBraun inert atmosphere glovebox containing an atmosphere of high purity argon. C_6D_6 was dried, degassed by standard procedures and stored over a potassium mirror. All other solvents were degassed by standard procedures and dried over activated 4Å mol sieves. $Ni(cod)_2$, ^{iPr}NHC (^{iPr}NHC = [{(H)CN(^{i}Pr)}$_2$C:]), ^{tBu}Xant (^{tBu}Xant = 2,7-di-*tert*-butyl-9,9-dimethylxanthene), ^{tBu}XantBr$_2$ (^{tBu}XantBr$_2$ = 4,5-dibromo-2,7-di-*tert*-butyl-9,9-dimethylxanthene), XantP$_2$ (XantP$_2$ = 4,5-(^{iPr}NHC·P)$_2$-9,9-dimethylxanthene) were synthesized according to known literature procedures.[1],[2],[3],[4] All other reagents were used as received. NMR spectra were recorded on a Bruker AV 400 Spectrometer. The ^{1}H and ^{13}C{^{1}H} NMR spectra were referenced to the residual solvent signals as internal standards. ^{31}P{^{1}H} NMR spectra were externally calibrated with H_3PO_4. Liquid Injection Field Desorption Ionization Mass Spectrometry (LIFDI-MS) was measured directly from an inert atmosphere glovebox with a Thermo Fisher Scientific Exactive Plus Orbitrap equipped with an ion source from Linden CMS.[5] Elemental analyses (C, H, N) were performed with a combustion analyzer (elementar vario EL, Bruker). Absorption spectra (UV/vis) were recorded on an Agilent Cary 60 UV/vis spectrophotometer.

Synthesis of 4,5-(PH$_2$)$_2$-2,7-di-*tert*-butyl-9,9-dimethylxanthene (^{tBu}Xant(PH$_2$)$_2$).

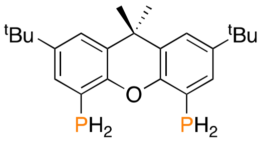

^{tBu}XantBr$_2$ (5.0 g, 10.4 mmol) was dissolved in 75 mL THF and the solution cooled to - 78 °C. To this cooled solution, *n*-BuLi (2.5 M in hexanes, 8.74 mL, 21.9 mmol) was added slowly over a period of 10 min. The mixture was stirred in the cold for 3h before adding ClP(OEt)$_2$ (3.14 mL, 21.9 mmol) at - 78 °C. The reaction mixture was stirred overnight. Volatiles were subsequently removed *in vacuo* and Et_2O (70 mL) added to yield a solution of ^{tBu}Xant(P(OEt)$_2$)$_2$. In a separate flask, A solution of $LiAlH_4$ (0.99 g, 26.0 mmol) in Et_2O (70 mL) was prepared and cooled to - 78 °C, followed by the addition of Me_3SiCl (3.30 mL, 26.0 mmol). The prepared solution of ^{tBu}Xant(P(OEt)$_2$)$_2$ in Et_2O was filtered directly to this mixture in a dropwise manner, the reaction mixture slowly warmed to RT and stirred for 16h. The reaction was subsequently quenched with degassed H_2O (45 mL), the organic and aqueous phases separated, and the aqueous phase extracted with Et_2O (3 x 30 mL). The combined organic phases were dried over dry $MgSO_4$. Volatiles were removed *in vacuo* to give ^{tBu}Xant(PH$_2$)$_2$ as an off-white solid, which was used without further purification.

^{1}H NMR (400 MHz, C_6D_6, 298 K): δ = 1.24 (s, 18H, ^{t}Bu-C*H*$_3$), 1.50 (s, 6H, Xant-C*H*$_3$), 4.16 (d, $^{1}J_{PH}$ = 202.6 Hz, 4H, P*H*$_2$), 7.36 (d, $^{4}J_{HH}$ = 2.3 Hz, 2H, Ar*H*), 7.44 (d, $^{3}J_{HH}$ = 6.8 Hz, 2H, Ar*H*).

^{13}C{^{1}H} NMR (101 MHz, C_6D_6, 298 K): δ = 31.6 (^{t}Bu-*C*H$_3$), 32.2 (Xant-*C*H$_3$), 34.6 (Xant-*C$_q$*), 35.2 (^{t}Bu-*C$_q$*), 117.5, 123.4, 129.5, 131.2, 146.0 and 150.3 (Ar*C*).

^{31}P{^{1}H} NMR (162 MHz, C_6D_6, 298 K): δ = - 140.7 (s, *P*H$_2$).

^{31}P NMR (162 MHz, C_6D_6, 298 K): δ = - 140.7 (tm, $^{1}J_{PH}$ = 202.1 Hz, *P*H$_2$).

Synthesis of ^{tBu}XantP$_2$, 1b.

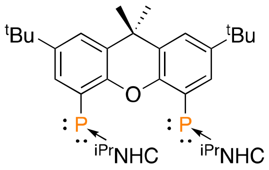

^{iPr}NHC (6.33 mL, 41.6 mmol) was added to a solution of ^{tBu}Xant(PH$_2$)$_2$ (4.02 g, 10.4 mmol) in toluene (70 mL), and the mixture heated to 100 °C for 48h. All volatiles were subsequently removed *in vacuo.* Pentane (40 mL) was added to the red oil and the reaction mixture sonicated in an ultrasonic bath for 30 min. The reaction mixture was filtered and the orange precipitate washed with pentane (2 x 30 mL). The precipitate was dried *in vacuo* at 80 °C to yield **1b** as an orange powder (2.44 g, 35%). Orange single crystals of **1b** suitable for SC-XRD analysis could be obtained from the pentane washing solutions.

^{1}H NMR (400 MHz, C_6D_6, 298 K): δ = 1.04 (d, $^{3}J_{HH}$ = 6.7 Hz, 24H, ^{i}Pr-C*H*$_3$), 1.31 (s, 18H, ^{t}Bu-C*H*$_3$), 1.77 (s, 6H, Xant-C*H*$_3$), 5.18 (tddd, *J* = 8.5, 6.7, 3.6, 1.8 Hz, 4H, ^{i}Pr-C*H*), 6.38 (s, 4H, ^{i}Pr-NC*H*), 7.18 (d, $^{4}J_{HH}$ = 2.4 Hz, 2H, Ar*H*), 7.44 (q, $^{4}J_{HH}$ = 2.6 Hz, 2H, Ar*H*).

^{13}C{^{1}H} NMR (101 MHz, C_6D_6, 298 K): δ = 22.2 (^{i}Pr-*C*H$_3$), 32.0 (^{t}Bu-*C*H$_3$), 32.7 (Xant-*C*H$_3$), 34.4 (Xant-*C$_q$*), 35.5 (^{t}Bu-*C$_q$*), 49.4 (^{i}Pr-*C*H), 114.9 (^{i}Pr-N*C*H), 117.1, 128.6, 136.0, 136.5, 143.4 and 150.5 (Ar*C*).

^{31}P{^{1}H} NMR (162 MHz, C_6D_6, 298 K): δ = - 72.8 (s, ^{iPr}NHC-*P*).

Anal.calcd. for $C_{41}H_{60}N_4OP_2$: C, 71.69%; H, 8.80%; N, 8.16%; found: C, 71.30%; H, 9.18%; N, 7.83%

MS/LIFDI-HRMS found (calcd.) m/z: 686.4218 (686.4241) for $[M]^+$.

λmax, nm (ε, Lmol^{-1} cm^{-1}): 416 (11309), 345 (9070)

Synthesis of DBF-P$_2$, 1c.

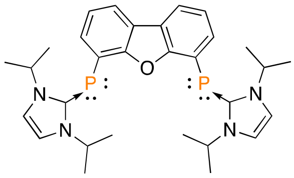

Dibenzofuran (DBF) (1.0 g, 5.95 mmol) was dissolved in a hexane-Et_2O mixture (1:2, 30 mL), TMEDA (1.8 mL, 11.9 mmol) added, and the reaction mixture cooled to - 78 °C. To this cooled solution, *n*-BuLi (2.5 M in hexanes, 5.0 mL, 12.5 mmol) was added leading to the formation of a yellow solution. After 30 min the cold bath was removed, and the solution heated to 40 °C for 2h. The reaction mixture was subsequently cooled to - 78 °C and $ClP(OEt)_2$ (1.7 mL, 11.9 mmol) added. Volatiles were removed *in vacuo* to give 4,6-$\{P(OEt)_2\}_2$-DBF. In a separate flask, a solution of $LiAlH_4$ (0.67 g, 17.8 mmol) in Et_2O (20 mL) was prepared, cooled to - 78 °C, and Me_3SiCl (1.8 mL, 14.2 mmol) added. The prepared 4,6-$\{P(OEt)_2\}_2$-DBF was dissolved in Et_2O and filtered directly onto the $LiAlH_4/Me_3SiCl$ mixture dropwise. The reaction mixture was slowly warmed to RT and stirred for 16 h, and subsequently quenched with degassed H_2O (10 mL). The organic and aqueous phases were separated, and the aqueous phase extracted with Et_2O (3 x 5 mL). The combined organic phases were dried over $MgSO_4$. Volatiles were removed *in vacuo* to give 4,6-$(PH_2)_2$-DBF, which was used directly in the next step.

^{1}H NMR (400 MHz, C_6D_6, 298 K): δ = 4.06 (d, $^1J_{PH}$ = 202.6 Hz, 4H, PH_2), 6.96 (t, $^3J_{HH}$ = 7.6 Hz, 2H, Ar*H*), 7.30 (t, $^3J_{HH}$ = 7.7 Hz, 2H, Ar*H*), 7.50 (d, $^3J_{HH}$ = 7.7 Hz, 2H, Ar*H*).

^{31}P{^{1}H} NMR (162 MHz, C_6D_6, 298 K): δ = - 154.0 (s, *P*H_2).

^{31}P NMR (162 MHz, C_6D_6, 298 K): δ = - 154.0 (td, $^1J_{PH}$ =202.7 Hz, $^3J_{PH}$ = 8.1 Hz, *P*H_2).

The as prepared 4,6-$(PH_2)_2$-DBF was dissolved in toluene, and ^{iPr}NHC (3.6 mL, 23.8 mmol) was added. The reaction mixture was heated to 90 °C for 16h to yield dark red crystals of **3** (661 mg, 21%) upon cooling to RT.

^{1}H NMR (400 MHz, C_6D_6, 298 K): δ = 0.95 (d, $^3J_{HH}$ = 6.7 Hz, 24H, ^{i}Pr-CH_3), 5.20 (pd, *J* = 6.7, 3.8 Hz, 4H, ^{i}Pr-C*H*), 6.41 (s, 4H, ^{i}Pr-NC*H*), 7.09 (t, $^3J_{HH}$ = 7.6 Hz, 2H, Ar*H*), 7.53 (dd, *J* = 7.5, 1.3 Hz, 2H, Ar*H*), 7.58 (ddd, *J* = 7.3, 5.8, 1.3 Hz, 2H, Ar*H*).

^{31}P{^{1}H} NMR (162 MHz, C_6D_6, 298 K): δ = - 86.0 (s, ^{iPr}NHC-*P*).

MS/LIFDI-HRMS found (calcd.) m/z: 532.2508 (532.2518) for $[M]^+$.

λmax, nm (ε, $Lmol^{-1}$ cm^{-1}): 455 (2853)

N.B. The acquisition of meaningful ^{13}C{^{1}H} NMR spectra of this compound was impeded by its limited solubility in available deuterated solvents.

Synthesis of $XantP_2(Ni(NHC)_2)_2$, 2a.

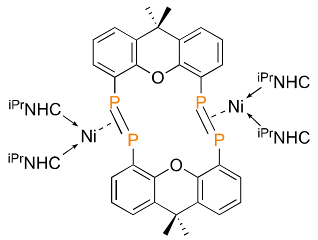

A flask was loaded with Xant-P_2 (500 mg, 0.87 mmol) and Ni(cod)$_2$ (263 mg, 0.96 mmol). Toluene (5 mL) was added and the reaction mixture heated to 100 °C for 4 days without stirring, leading to formation of large red crystals. The reaction mixture was filtered, the solid residue washed with CH_3CN, and dried *in vacuo* to yield **4** as dark red crystals (220 mg, 41%). Single crystals suitable for a SC-XRD measurement could be isolated directly from the reaction mixture.

^{1}H NMR (400 MHz, THF-d_8, 333 K): δ = 1.09 (br s, 24H, ^{i}Pr-C*H$_3$*), 1.31 (s, 6H, Xant-C*H$_3$*), 1.50 (s, 6H, Xant-C*H$_3$*), 5.33 (br s, 8H, ^{i}Pr-C*H*), 6.50 (t, $^{3}J_{HH}$ = 7.4 Hz, 4H, Ar*H*), 6.85 (dd, *J* = 7.6, 1.6 Hz, 4H, Ar*H*), 6.89 (br s, 8H, ^{i}Pr-NC*H*), 7.37 (d, $^{3}J_{HH}$ = 7.4 Hz, 4H, Ar*H*).

^{31}P{^{1}H} NMR (162 MHz, THF-d_8, 298 K): δ = - 47.1 (s, ^{iPr}NHC$_2$Ni*P*$_2$).

MS/LIFDI-HRMS found (calcd.) m/z: 1264.4634 (1264.4604) for [M]$^+$.

λmax, nm (ε, Lmol^{-1} cm^{-1}): 357 (22386), 310 (32089).

N.B. The acquisition of meaningful ^{1}H and ^{13}C{^{1}H} NMR spectra of this compound was impeded by its limited solubility in available deuterated solvents.

Synthesis of ^{tBu}XantP$_2$(Ni(NHC)$_2$)$_2$, 2b.

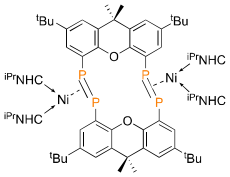

A flask was loaded with ^{tBu}XantP$_2$ (500 mg, 0.73 mmol) and Ni(cod)$_2$ (200 mg, 0.73 mmol). Toluene (5 mL) was added and the reaction mixture heated to 100 °C for 6 days without stirring, leading to the formation of large red crystals. The reaction mixture was filtered, washed with CH_3CN and dried *in vacuo* to yield **3** as red crystals (190 mg, 35%). Single crystals suitable for a SC-XRD measurement could be isolated directly from the reaction mixture.

^{1}H NMR (400 MHz, THF-d_8, 298 K): δ = 0.59 d, $^{3}J_{HH}$ = 6.7 Hz, 6H, ^{i}Pr-C*H$_3$*), 0.66 (d, $^{3}J_{HH}$ = 6.8 Hz, 6H, ^{i}Pr-C*H$_3$*), 0.74 (dd, *J* = 7.0, 3.1 Hz, 12H, ^{i}Pr-C*H$_3$*), 1.00 (d,

$^3J_{HH}$ = 6.8 Hz, 6H, ^{i}Pr-CH_3), 1.07 (s, 18H, ^{t}Bu-CH_3), 1.27 (s, 6H, Xant-CH_3), 1.39 (s, 18H, ^{t}Bu-CH_3), 1.52 (s, 6H, Xant-CH_3), 1.57 (d, $^3J_{HH}$ = 6.8 Hz, 6H, ^{i}Pr-CH_3), 1.66 (d, $^3J_{HH}$ = 6.4 Hz, 6H, ^{i}Pr-CH_3), 1.94 (d, $^3J_{HH}$ = 5.1 Hz, 6H, ^{i}Pr-CH_3), 4.61 (m, 2H, ^{i}Pr-CH), 5.04 (m, 2H, ^{i}Pr-CH), 5.51 (m, 2H, ^{i}Pr-CH), 6.28 (m, 2H, ^{i}Pr-CH), 6.69 (d, $^4J_{HH}$ = 2.0 Hz, 2H, ArH), 6.83 (s, 2H, ArH), 6.88 (br s, 2H, ArH), 6.91 (s, 2H, ArH), 6.96 (d, $^4J_{HH}$ = 2.4 Hz, 2H, ArH), 7.17 (s, 2H, ArH), 7.80 (s, 2H, ArH).

^{13}C{^{1}H} NMR (101 MHz, THF-d_8, 298 K): δ = 23.1, 23.2, 23.3, 23.4, 23.8, 24.5 and 24.8 (^{i}Pr-CH$_3$), 30.9 and 32.1 (Xant-CH$_3$), 32.4 and 32.7 (^{t}Bu-CH$_3$), 34.7 (Xant-C_q), 35.4 and 36.2 (^{t}Bu-C_q), 51.8 and 52.0 (^{i}Pr-CH), 115.9, 116.7, 116.9, 117.2, 117.5, 117.7, 129.2, 131.1, 141.8, 152.2 (ArC).

^{31}P{^{1}H} NMR (162 MHz, THF-d_8, 298 K): δ = - 42.7 – - 48.4 (m, ^{iPr}NHC$_2$NiP_2).

Anal.calcd. for $C_{82}H_{120}N_8O_2P_4$: C, 66.05%; H, 8.11%; N, 7.51%; found: C, 64.23%; H, 8.00%; N, 7.23%.

N.B. Repeated elemental analysis gave variable but consistently low values for C, possibly due to Ni-carbide formation.

MS/LIFDI-HRMS found (calcd.) m/z: 1490.7163 (1490.7112) for [M]$^+$.

λmax, nm (ε, Lmol^{-1} cm^{-1}): 513 (2012), 364 (33850), 313 (23657).

Synthesis of ^{tBu}XantP$_2$(NiNHC)$_2$, 3, and [^{tBu}XantP$_2${Ni(Cl)NHC}$_2$(CuNHC)$_2$], 4.

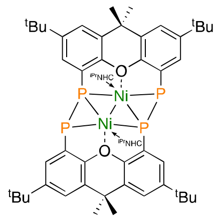

^{tBu}XantP$_2$(Ni(NHC)$_2$)$_2$ (100 mg, 0.067 mmol) was dissolved in THF (15 mL) and cooled to - 80 °C, a solution of CuCl (13.3 mg, 0.134 mmol) in CH_3CN (0.5 mL) added dropwise *via* syringe, and the reaction mixture stirred overnight. The reaction mixture was filtered, and the solvent concentrated to ~ 3 mL. After 48h storage of this solution at RT dark green crystals of **3** (27 mg, 24%) suitable for X-ray crystallography could be isolated. Conducting the same reaction in acetonitrile, without stirring, leads to the formation of a small amount (<5%) of dark red crystalline blocks, which were found to be compound **4**. Due to the low-yield and insolubility of this compound in common organic solvents, no data aside from its X-ray crystal structure were collected.

^{1}H NMR (400 MHz, THF-d_8, 298 K): δ = 0.74 (br s, 24H, ^{i}Pr-CH_3), 1.34 (s, 36H, ^{t}Bu-CH_3), 1.53 (s, 6H, Xant-CH_3), 1.85 (s, 6H, Xant-CH_3), 4.76 (p, $^3J_{HH}$ = 6.7 Hz, 4H, ^{i}Pr-CH), 6.84 (s, 4H, ^{i}Pr-NCH), 7.36 (d, $^4J_{HH}$ = 2.3 Hz, 4H, ArH), 7.67 (d, $^4J_{HH}$ = 3.9 Hz, 4H, ArH).

$^{31}P\{^1H\}$ NMR (162 MHz, THF-d_8, 298 K): δ = 129.4 (br d, $^1J_{PP}$ = 407.9 Hz), -81.2 (br d, $^1J_{PP}$ = 447.0 Hz).

MS/LIFDI-HRMS found (calcd.) m/z: 1186.4559 (1186.4496) for $[M]^+$.

λmax, nm (ε, $Lmol^{-1}$ cm^{-1}): 614 (1199), 416 (6065), 367 (9112)

N.B. The acquisition of meaningful $^{13}C\{^1H\}$ NMR spectra of this compound was impeded by its limited solubility in available deuterated solvents.

Synthesis of 1b·$GeCl_2$.

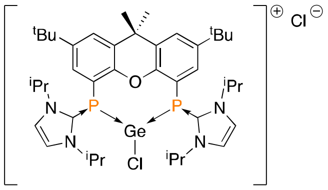

$GeCl_2$· dioxane (17 mg, 0.072 mmol) and **1b** (50 mg, 0.072 mmol) were dissolved in CH_3CN (0.5 mL) and layered with *n*-pentane (5 mL). Yellow crystals of [^{tBu}XantP_2·GeCl]Cl (117 mg, 48%), which were suitable for SC-XRD could be isolated from the reaction mixture.

^1H NMR (400 MHz, CD_3CN, 298 K): δ = 1.34 (d, $^3J_{HH}$ = 6.6 Hz, 6H, ^{i}Pr-CH_3), 1.43 (d, $^3J_{HH}$ = 6.7 Hz, 6H, ^{i}Pr-CH_3), 1.49 (s, 3H, Xant-CH_3), 1.57 (dd, J = 12.0, 6.7 Hz, 12H, ^{i}Pr-CH_3), 1.80 (s, 3H, Xant-CH_3), 4.85 (p, $^3J_{HH}$ = 6.6 Hz, 2H, ^{i}Pr-C*H*), 5.55 (br s, 2H, ^{i}Pr-C*H*), 6.81 (d, $^4J_{HH}$ = 2.3 Hz, 2H, Ar*H*), 7.46 (d, $^4J_{HH}$ = 2.0 Hz, 2H Ar*H*), 7.72 (s, 2H, ^{i}Pr-NC*H*), 7.76 (s, 2H, ^{i}Pr-NC*H*).

$^{31}P\{^1H\}$ NMR (162 MHz, CD_3CN, 298 K): δ = - 72.4 (s, P_2-GeCl).

MS/LIFDI-HRMS found (calcd.) m/z: 795.3124 (795.3105) for $[^{tBu}XantP_2{\cdot}GeCl]^+$.

N.B. The acquisition of meaningful $^{13}C\{^1H\}$ NMR spectra of this compound was impeded by its limited solubility in available deuterated solvents.

NMR, MS, IR, and UV/vis plots:

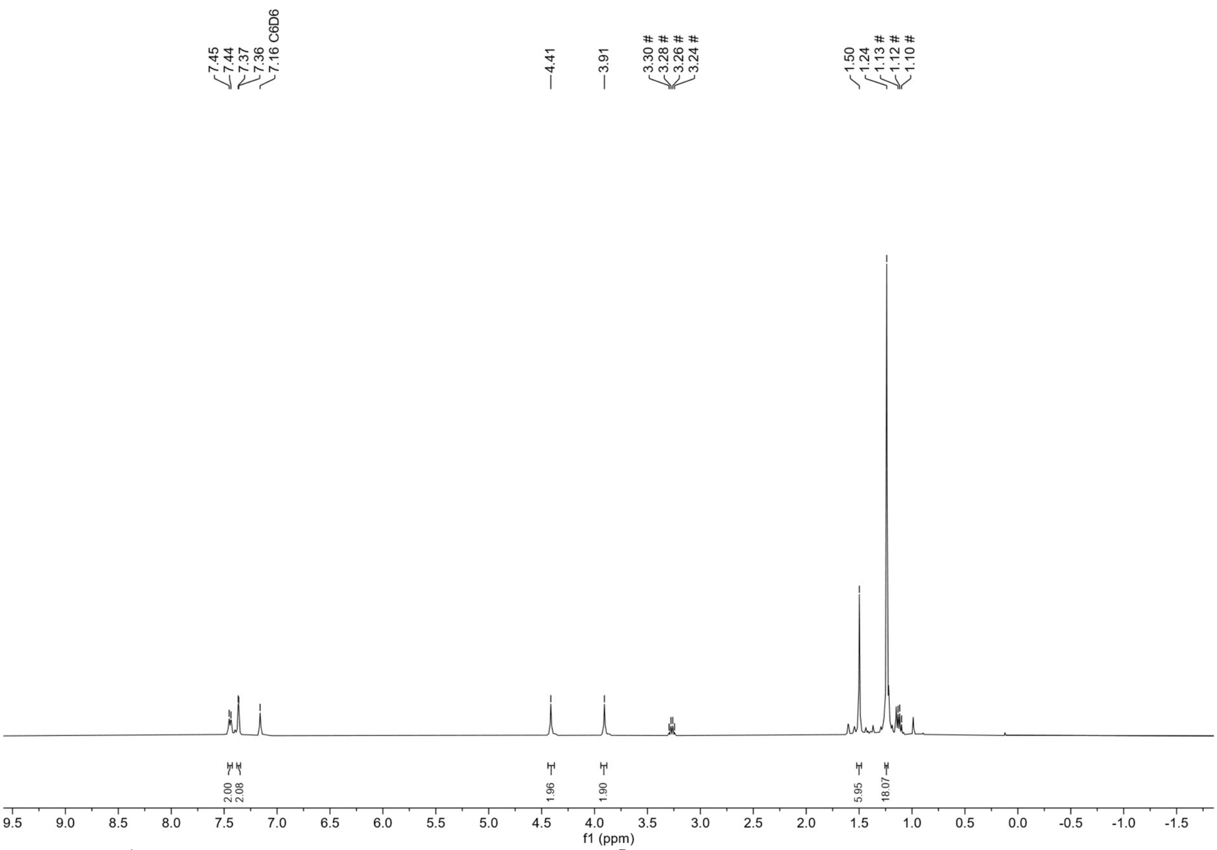

Figure S1. ^{1}H NMR spectrum (400 MHz, C_6D_6, 298 K) of ^{tBu}Xant(PH_2)$_2$. # denotes minor amounts of Et_2O.

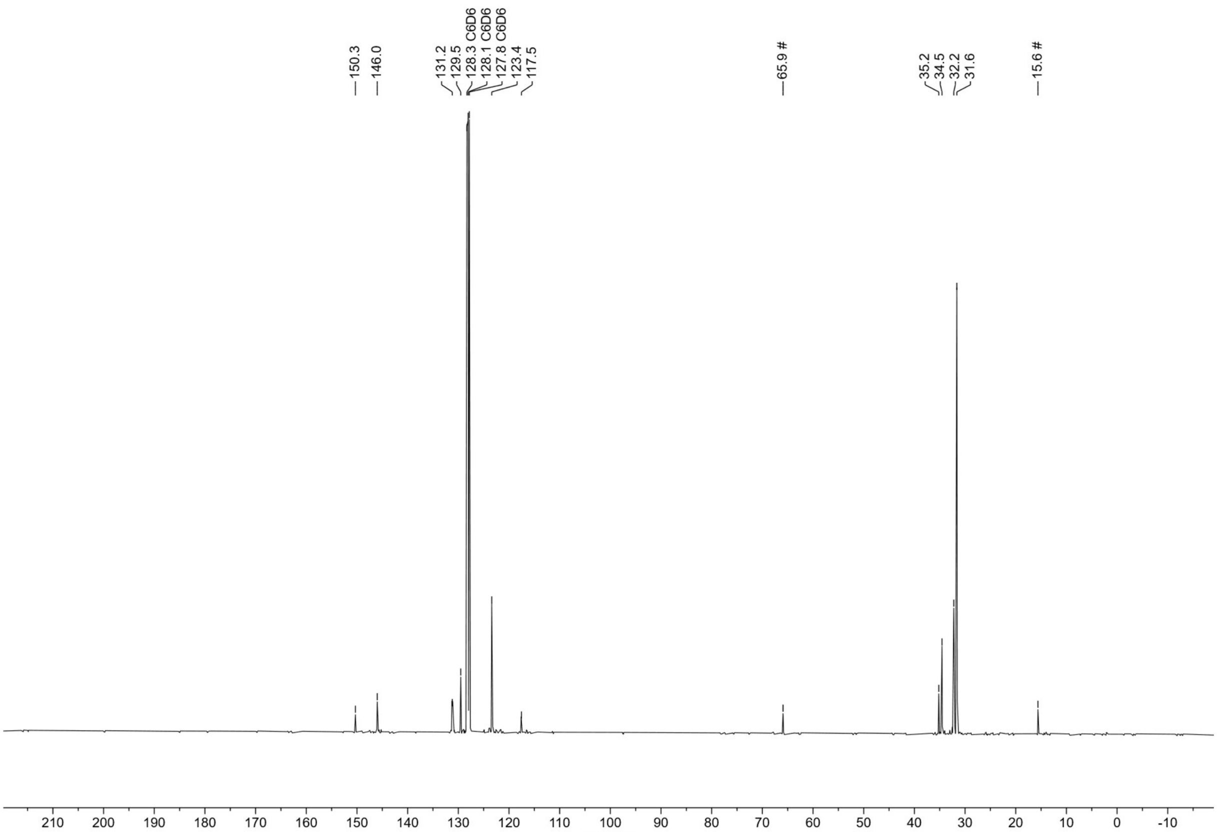

Figure S2. $^{13}C\{^{1}H\}$ NMR spectrum (101 MHz, C_6D_6, 298 K) of ^{tBu}Xant(PH_2)$_2$. # denotes minor amounts of Et_2O.

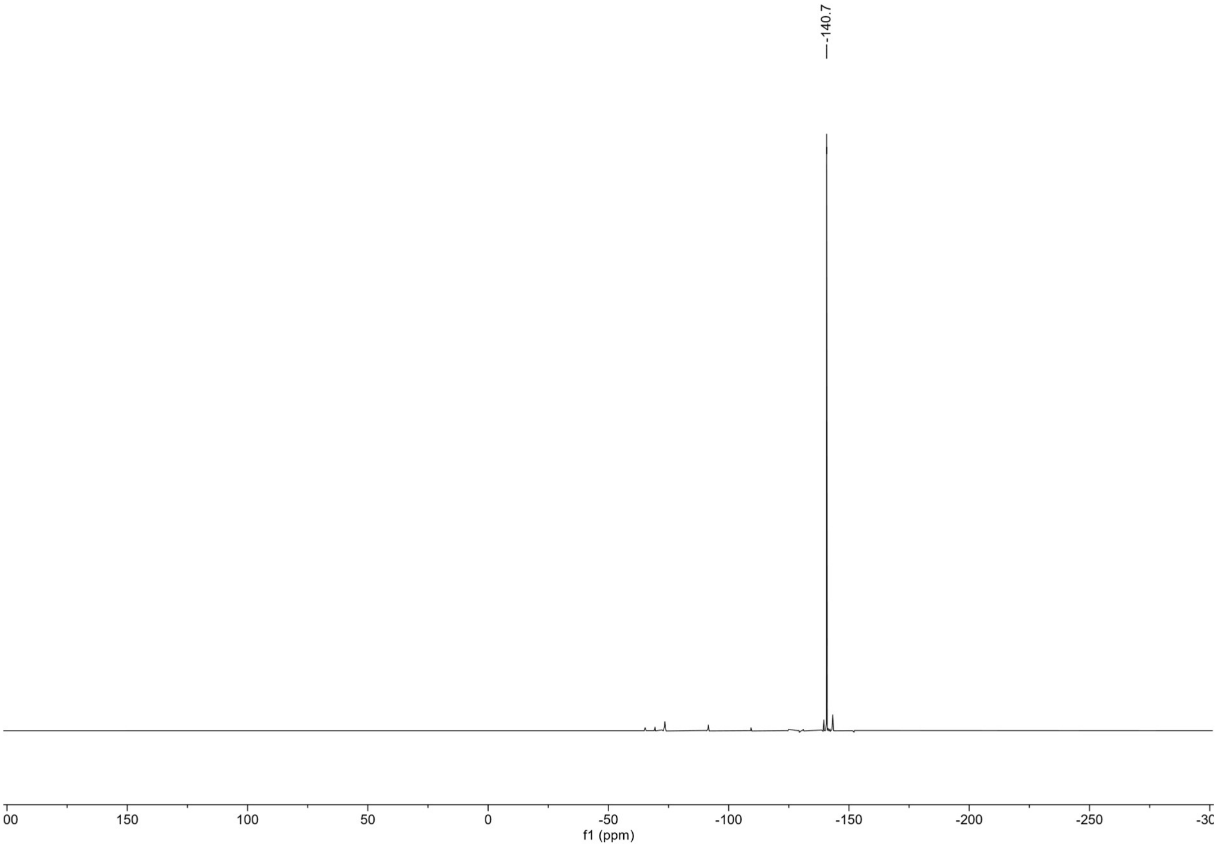

Figure S3. $^{31}P\{^{1}H\}$ NMR spectrum (162 MHz, C_6D_6, 298 K) of ^{tBu}Xant$(PH_2)_2$.

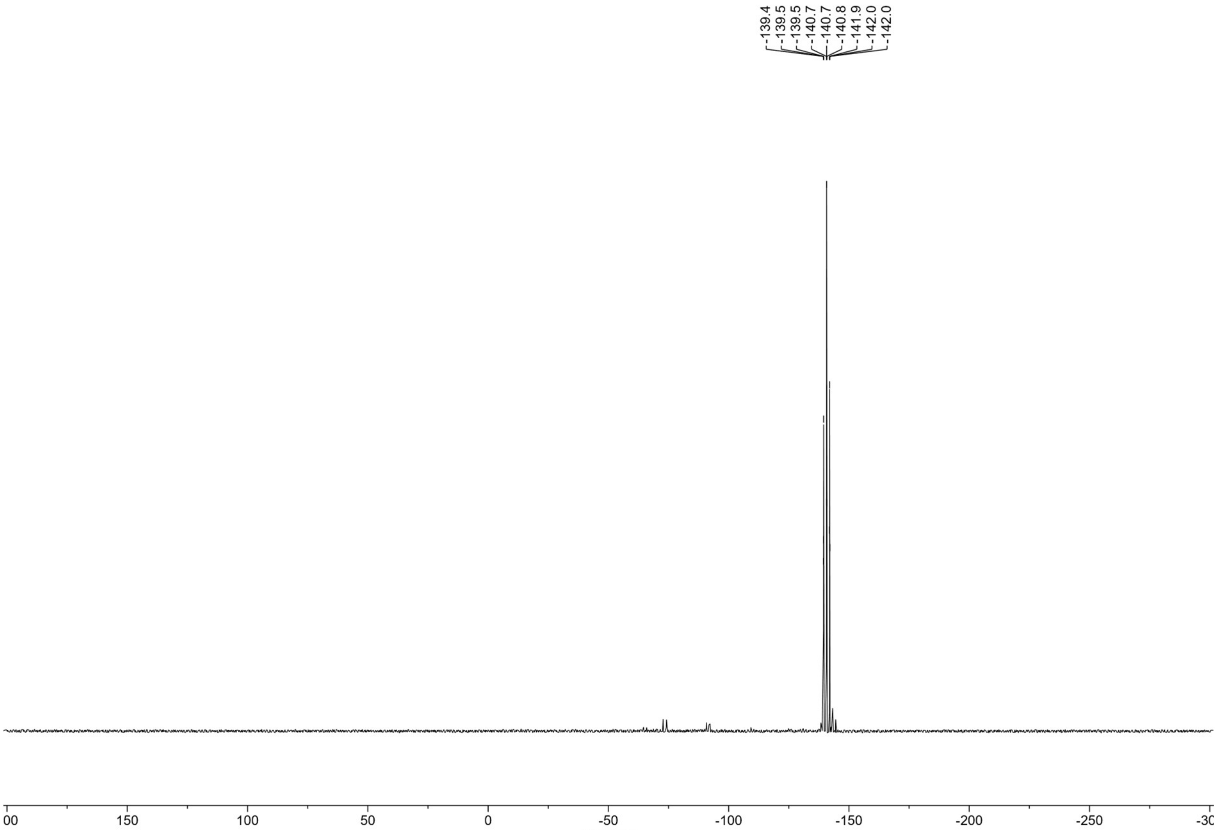

Figure S4. ^{31}P NMR spectrum (162 MHz, C_6D_6, 298 K) of ^{tBu}Xant$(PH_2)_2$.

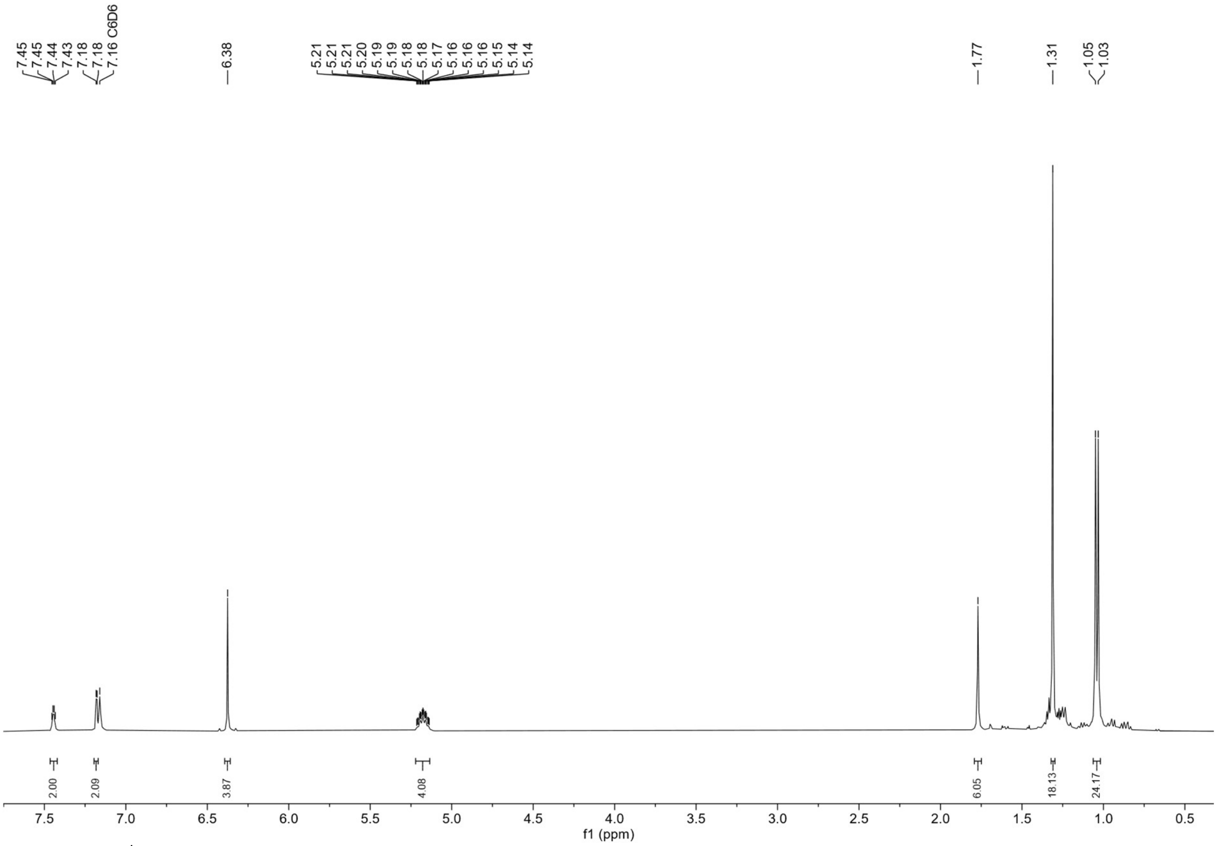

Figure S5. ^{1}H NMR spectrum (400 MHz, C_6D_6, 298 K) of **1b**.

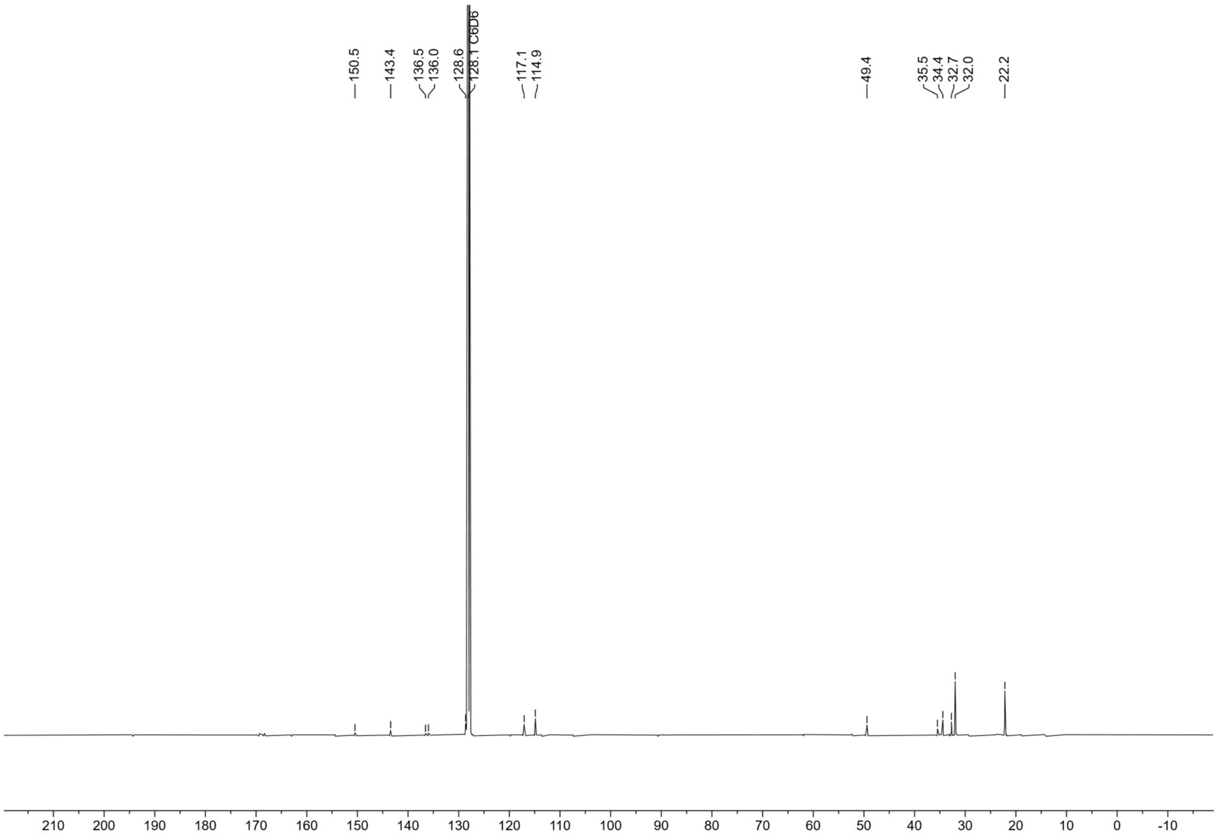

Figure S6. $^{13}C\{^1H\}$ NMR spectrum (101 MHz, C_6D_6, 298 K) of **1b**.

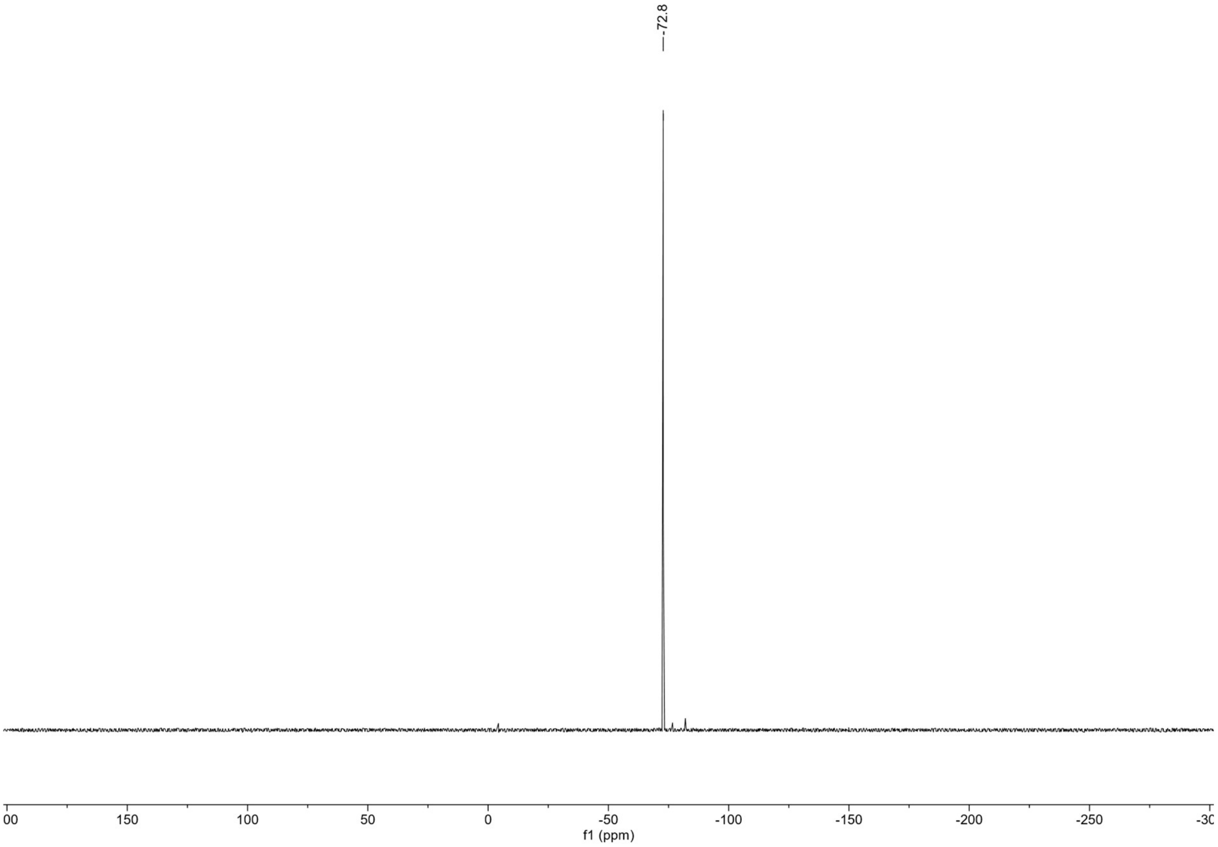

Figure S7. $^{31}P\{^{1}H\}$ NMR spectrum (162 MHz, C_6D_6, 298 K) of **1b**.

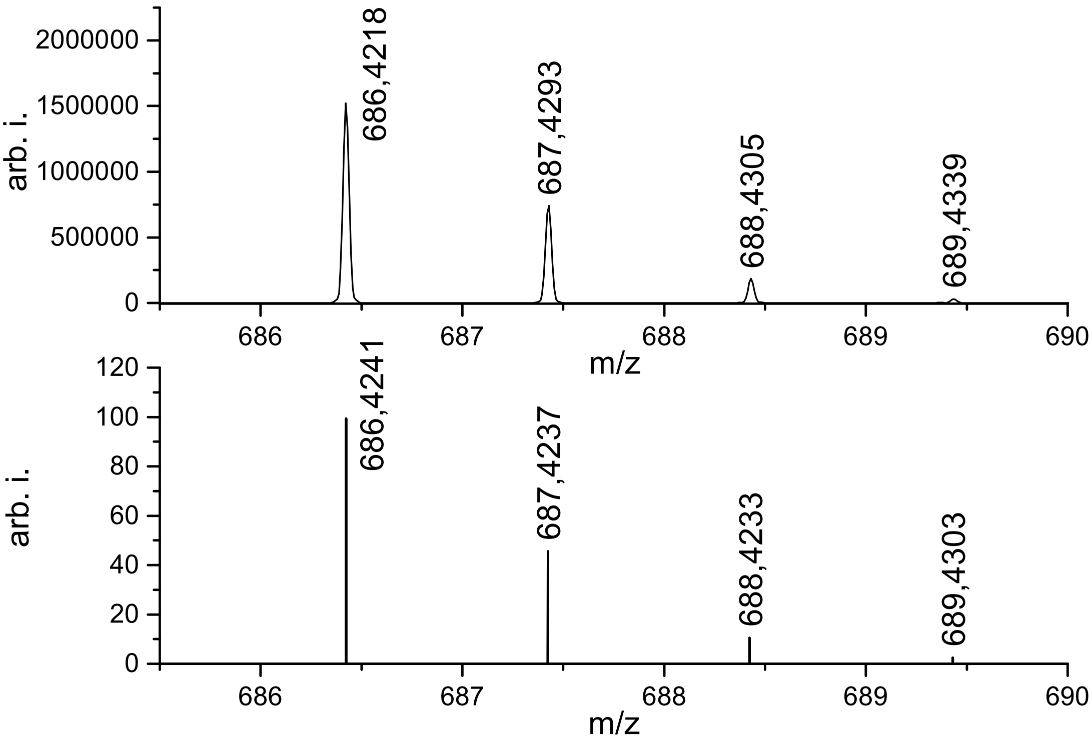

Figure S8. Cutout from LIFDI/MS of **1b**, Top: found MS for $[^{tBu}XantP_2]^+$; Bottom: Calculated MS spectrum of $[^{tBu}XantP_2]^+$.

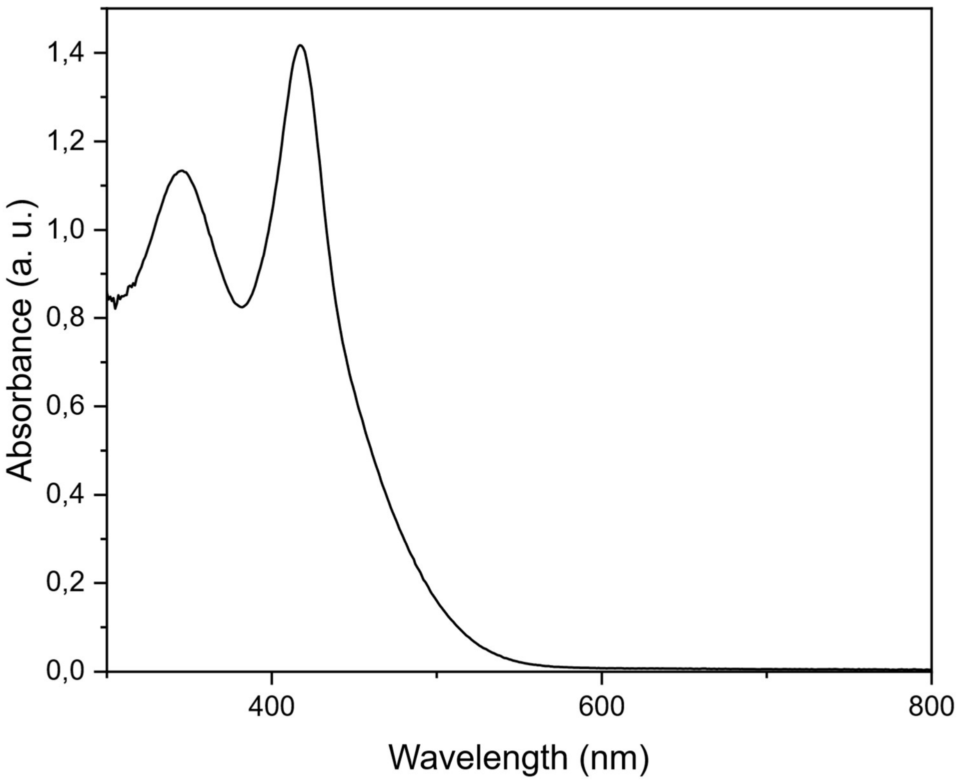

Figure S9. UV/Vis spectrum of a $1.25x10^{-4}$ M solution of **1b** in toluene at ambient temperature.

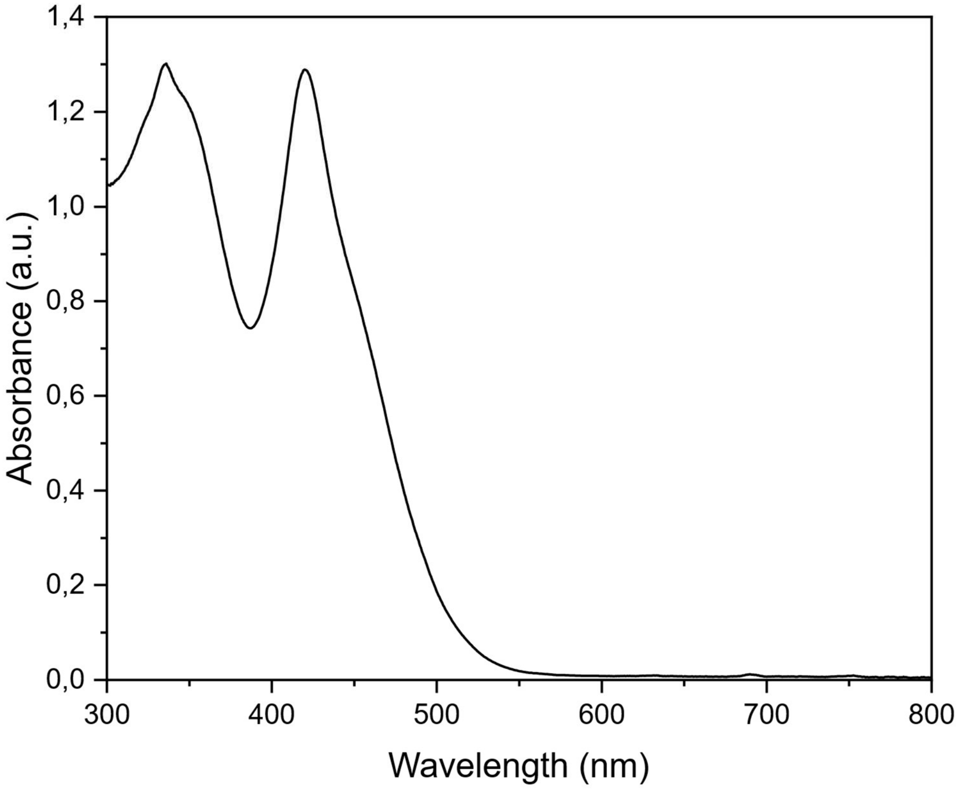

Figure S10. UV/Vis spectrum of a $6.25x10^{-5}$ M solution of **1a**, for comparison, in toluene at ambient temperature.

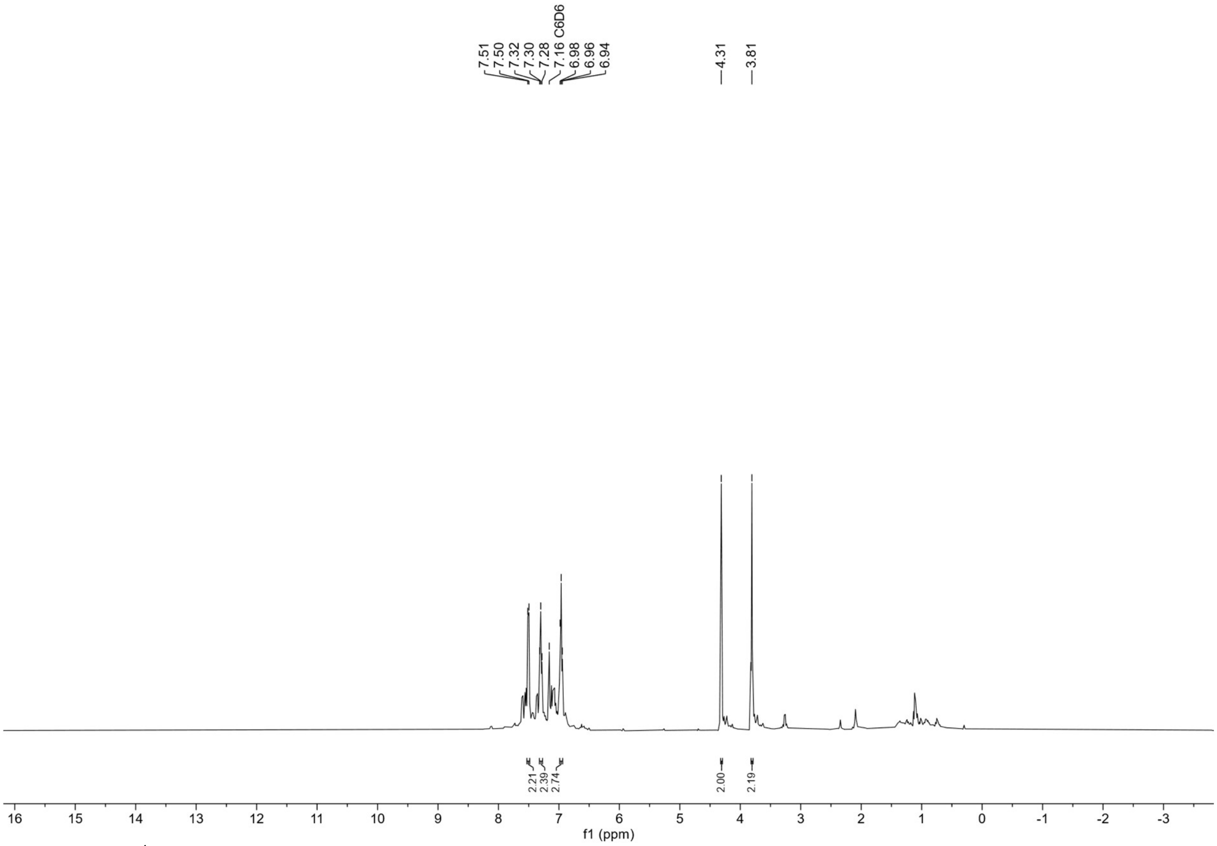

Figure S11. ^{1}H NMR spectrum (400 MHz, C_6D_6, 298 K) of 4,6-$(PH_2)_2$-DBF.

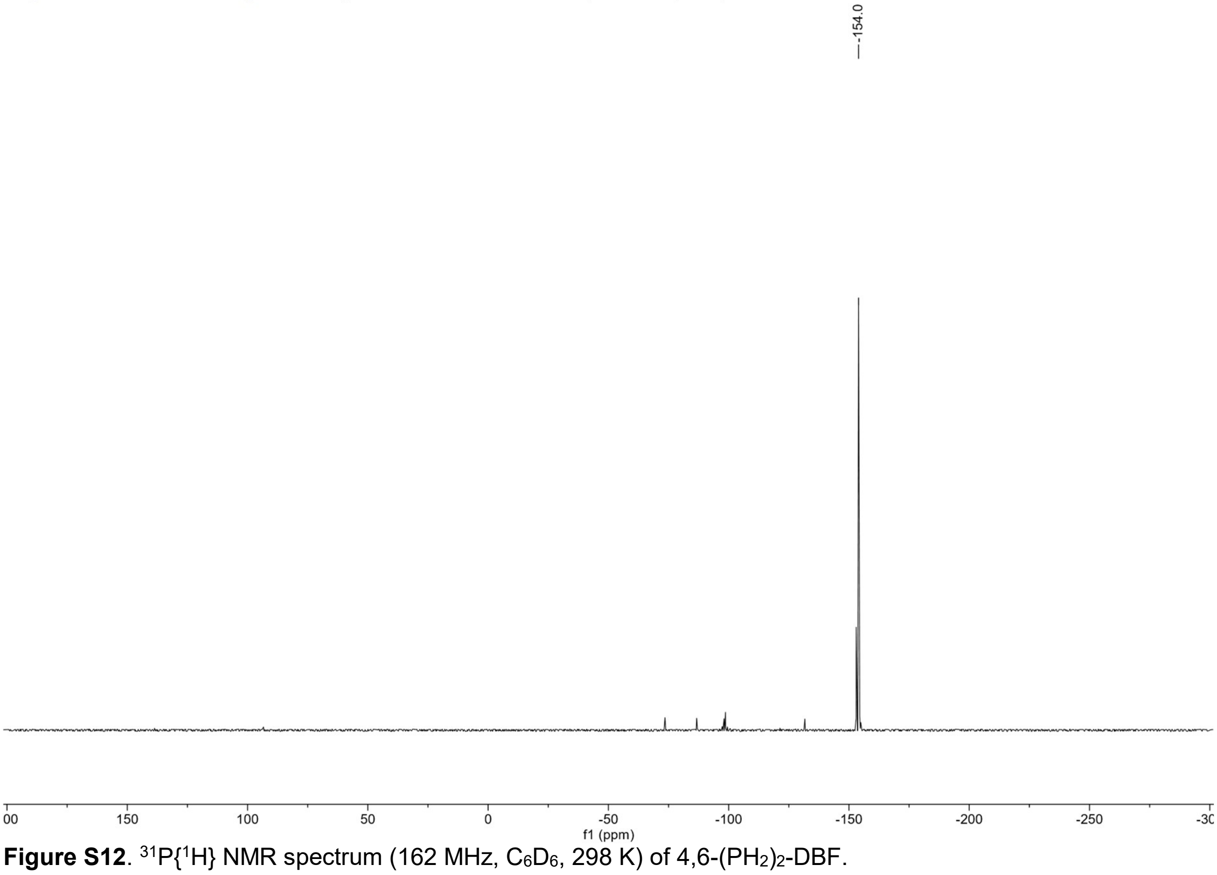

Figure S12. $^{31}P\{^{1}H\}$ NMR spectrum (162 MHz, C_6D_6, 298 K) of 4,6-$(PH_2)_2$-DBF.

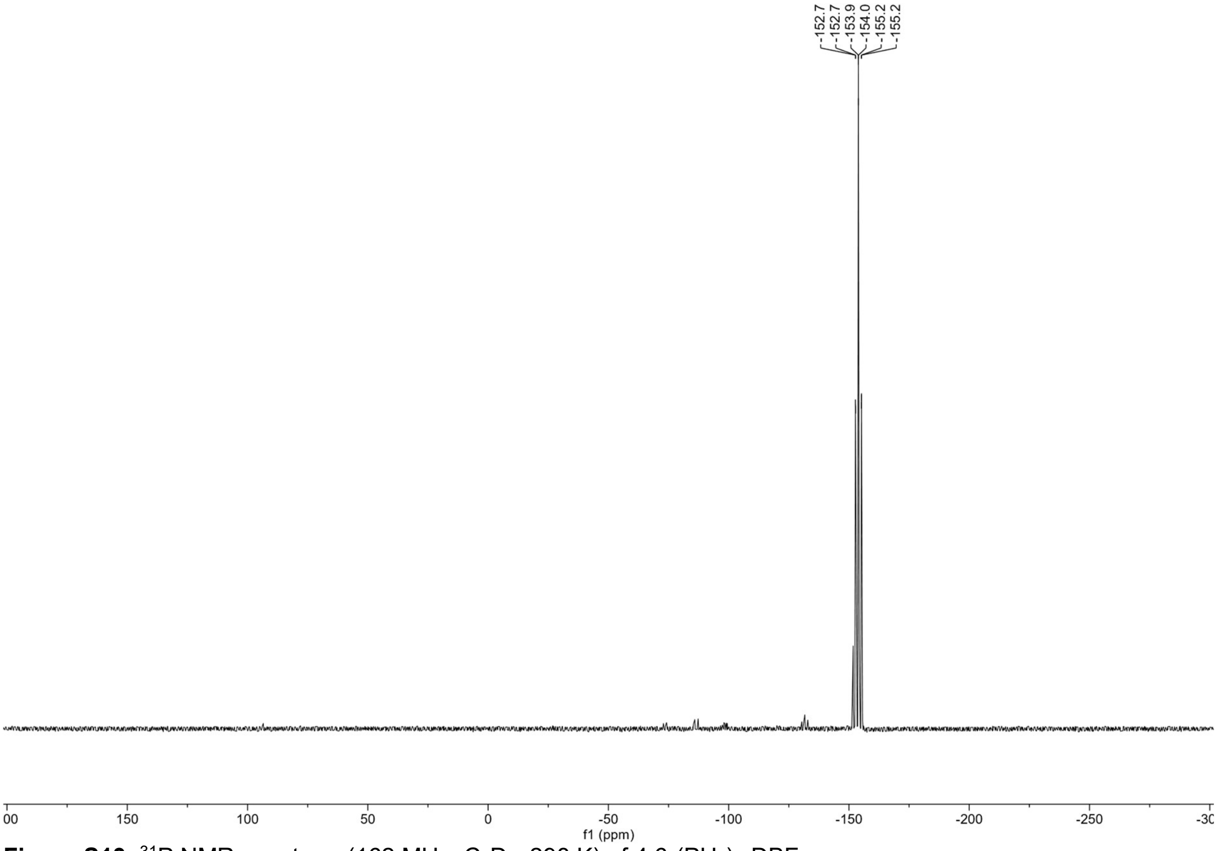

Figure S13. ^{31}P NMR spectrum (162 MHz, C_6D_6, 298 K) of 4,6-$(PH_2)_2$-DBF.

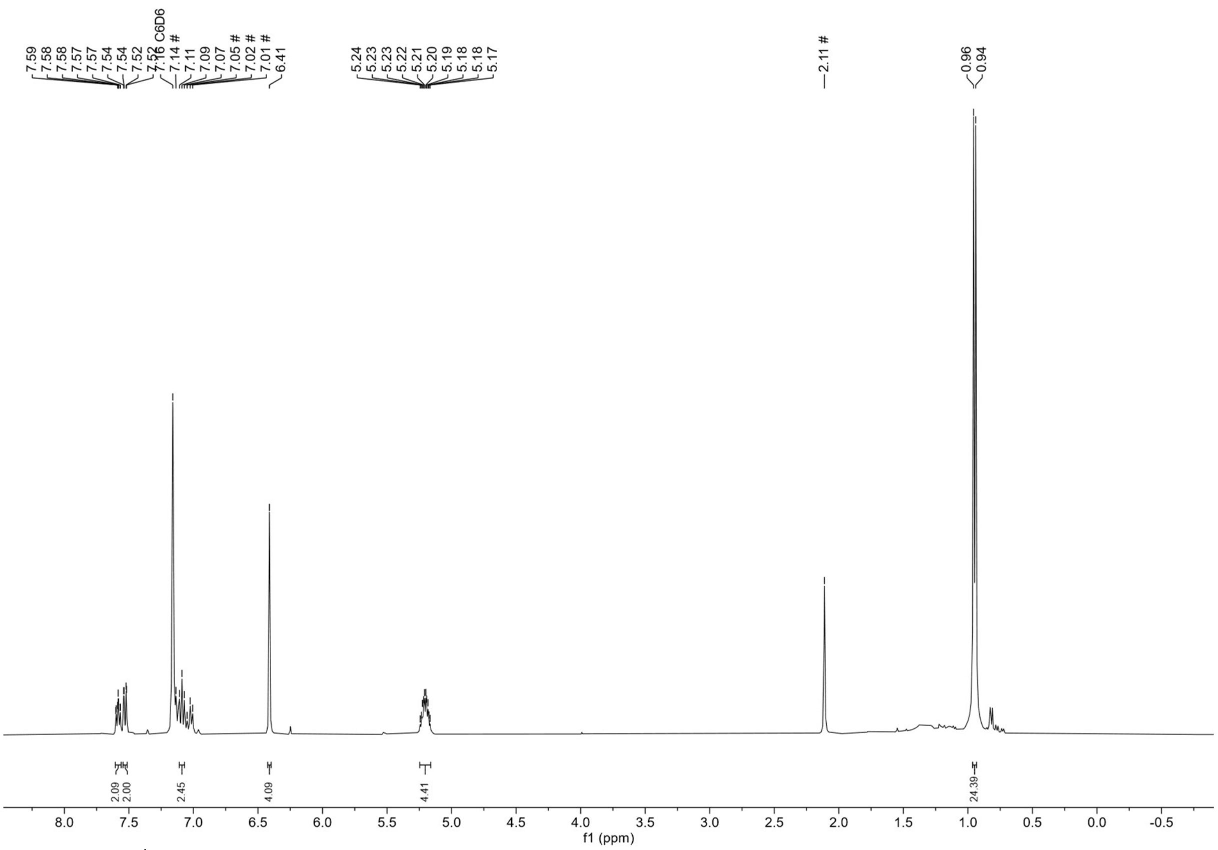

Figure S14. ^{1}H NMR spectrum (400 MHz, C_6D_6, 298 K) of **1c**. # denotes minor amounts of toluene.

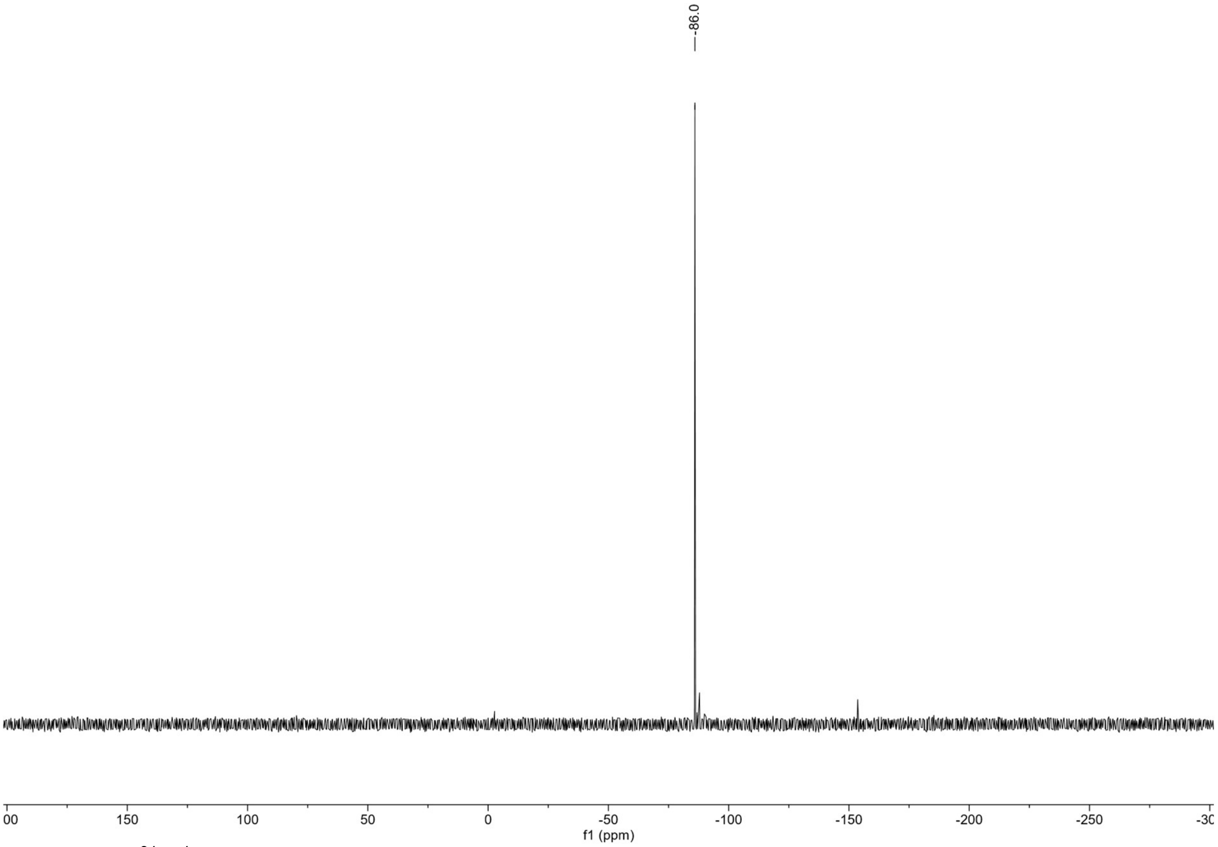

Figure S15. $^{31}P\{^1H\}$ NMR spectrum (162 MHz, C_6D_6, 298 K) of **1c**.

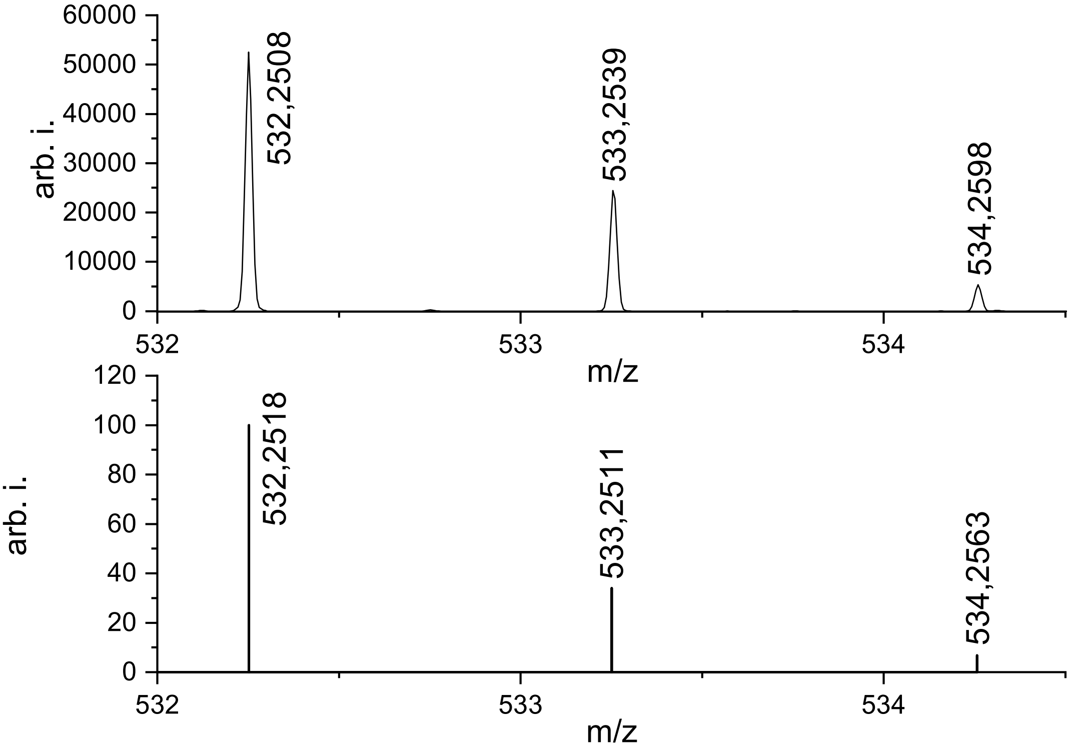

Figure S16. Cutout from LIFDI/MS of **1c**, Top: found MS for $[DBF\text{-}P_2]^+$; Bottom: Calculated MS spectrum of $[DBF\text{-}P_2]^+$.

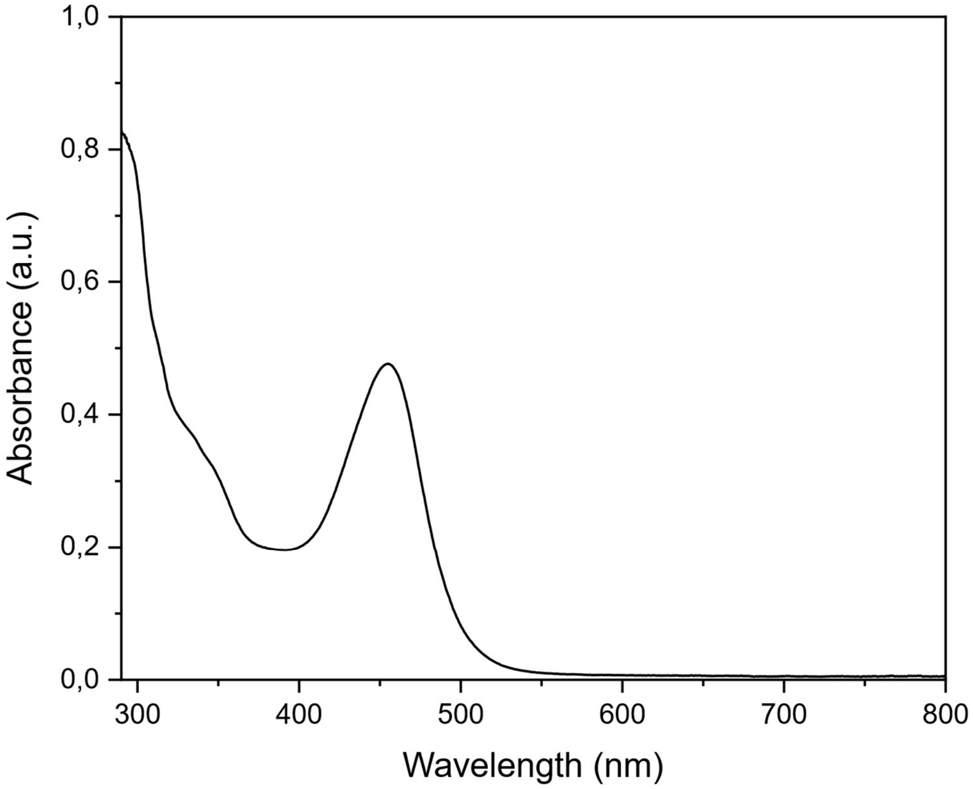

Figure S17. UV/Vis spectrum of a 1.67x10^{-4} M solution of **1c** in toluene at ambient temperature.

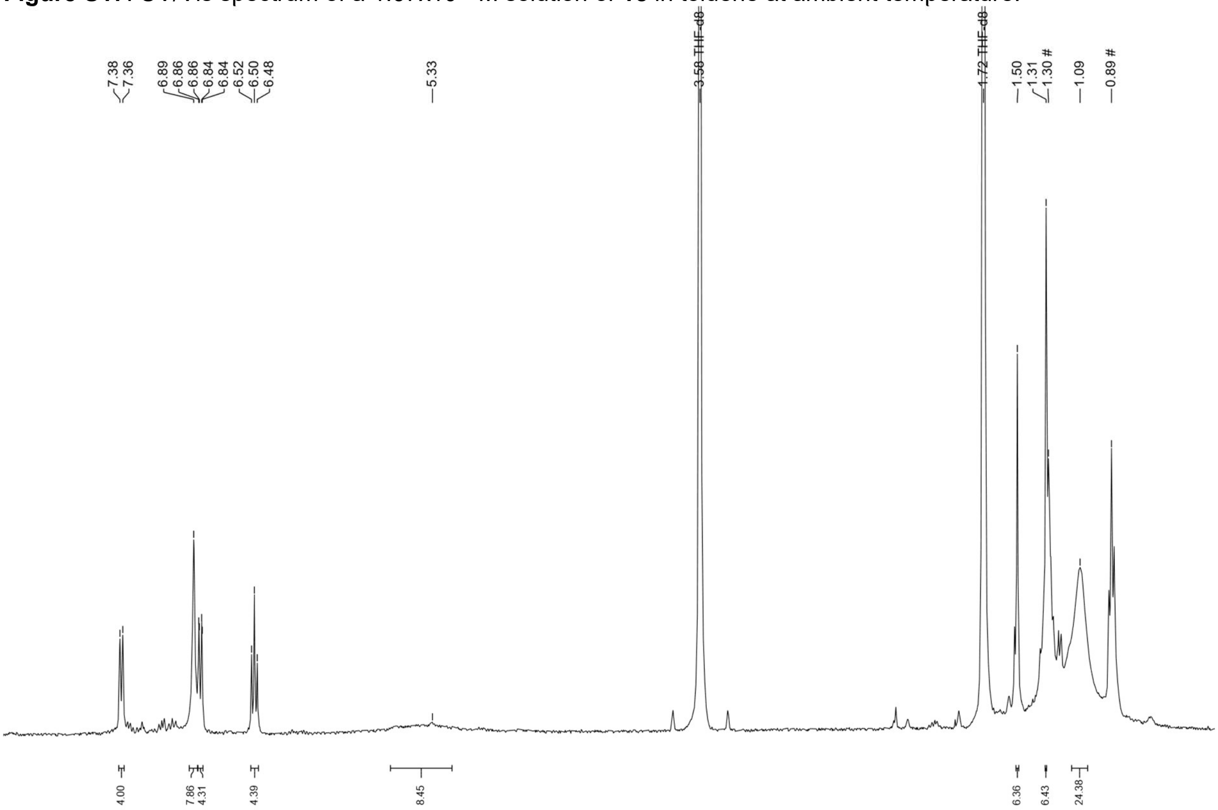

Figure S18. ^{1}H NMR spectrum (400 MHz, THF-d_8, 333 K) of **2a**. # denotes minor amounts of hexane.

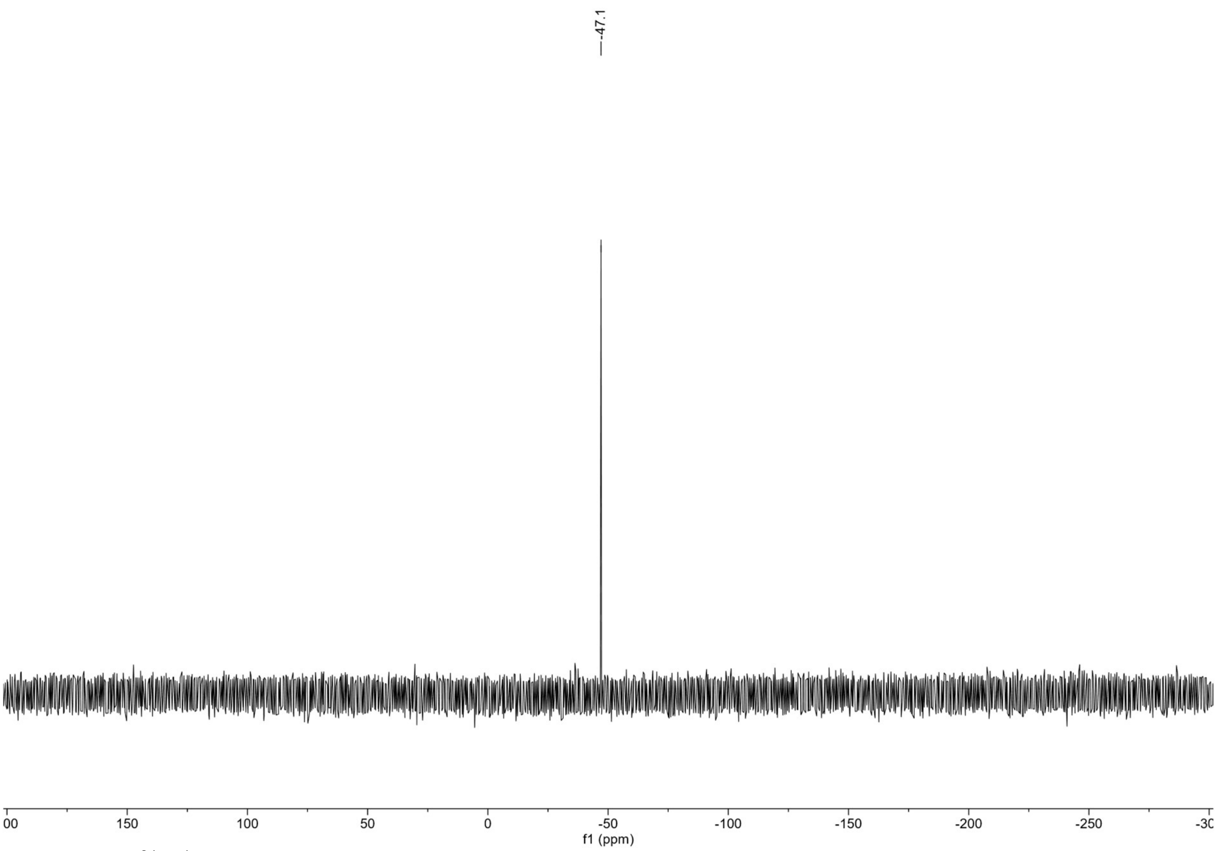

Figure S19. $^{31}P\{^{1}H\}$ NMR (162 MHz, THF-d_8, 298 K) of **2a.**

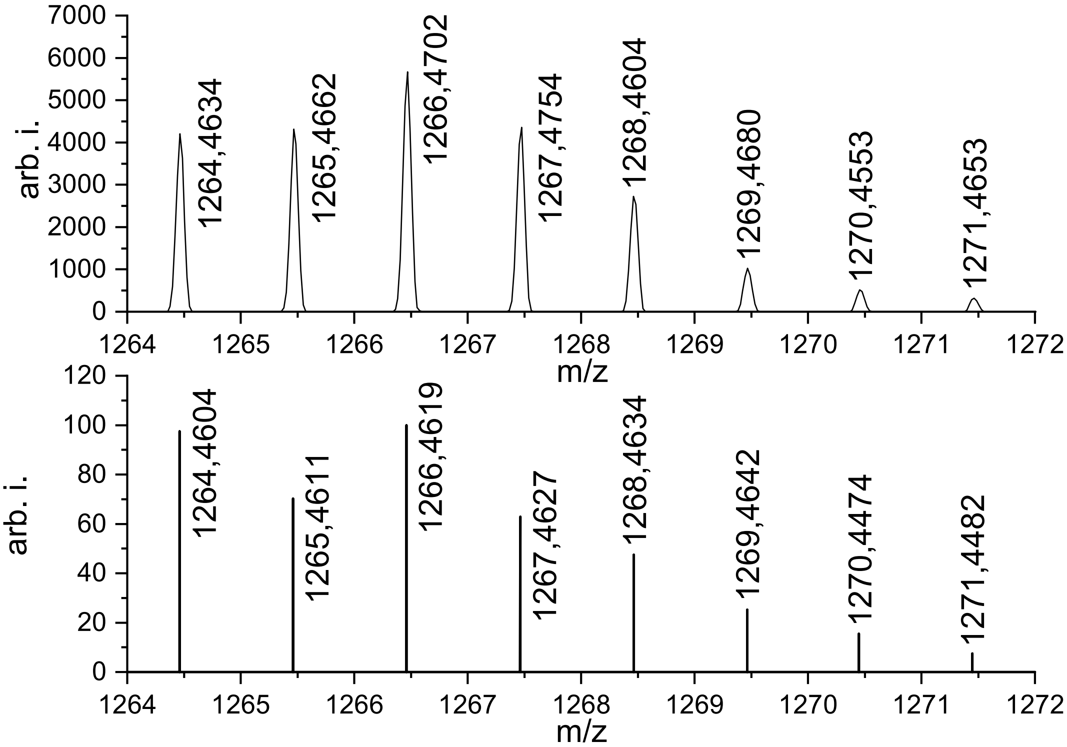

Figure S20. Cutout from LIFDI/MS of **2a**, Top: found MS for $[XantP_2(Ni(NHC)_2)_2]^+$; Bottom: Calculated MS spectrum of $[XantP_2(Ni(NHC)_2)_2]$.

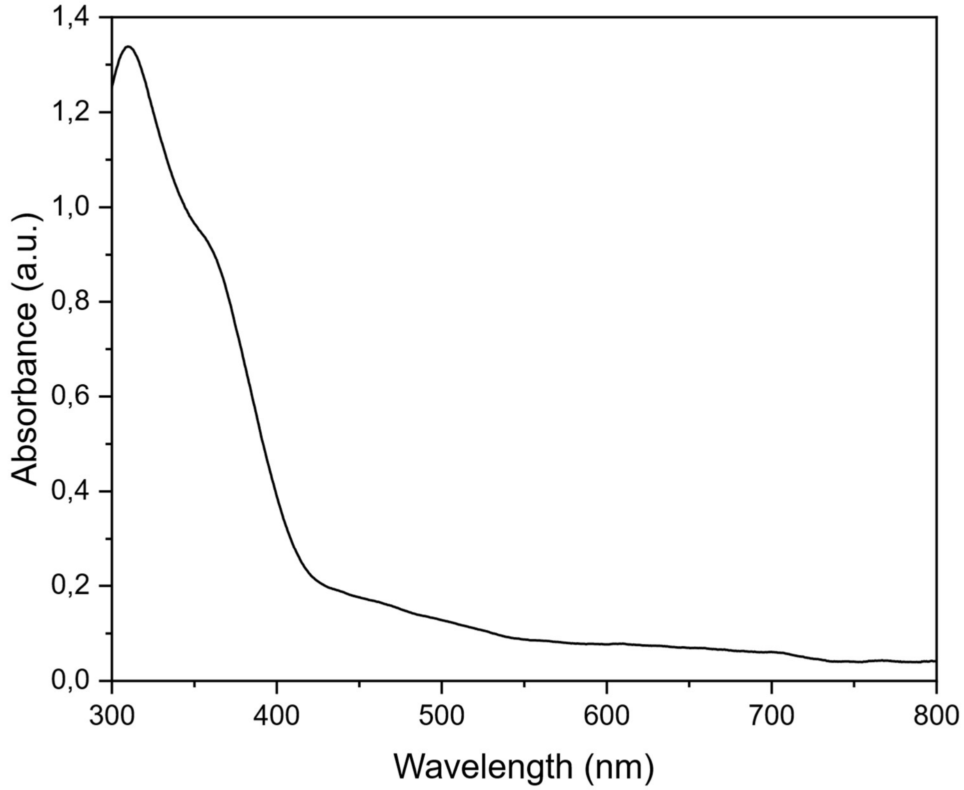

Figure S21. UV/Vis spectrum of a 4.17x10^{-5} M solution of **2a** in THF at ambient temperature.

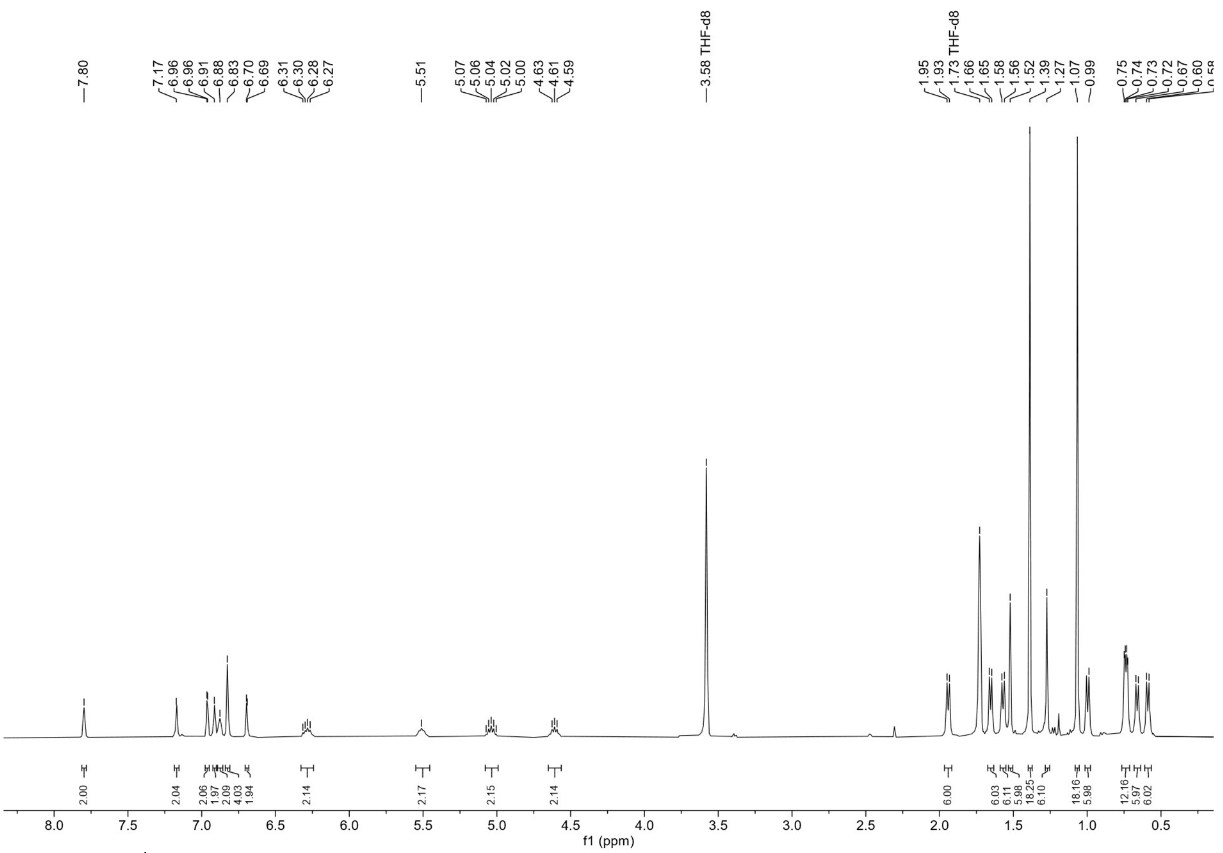

Figure S22. ^{1}H NMR spectrum (400 MHz, THF-d_8, 298 K) of **2b**.

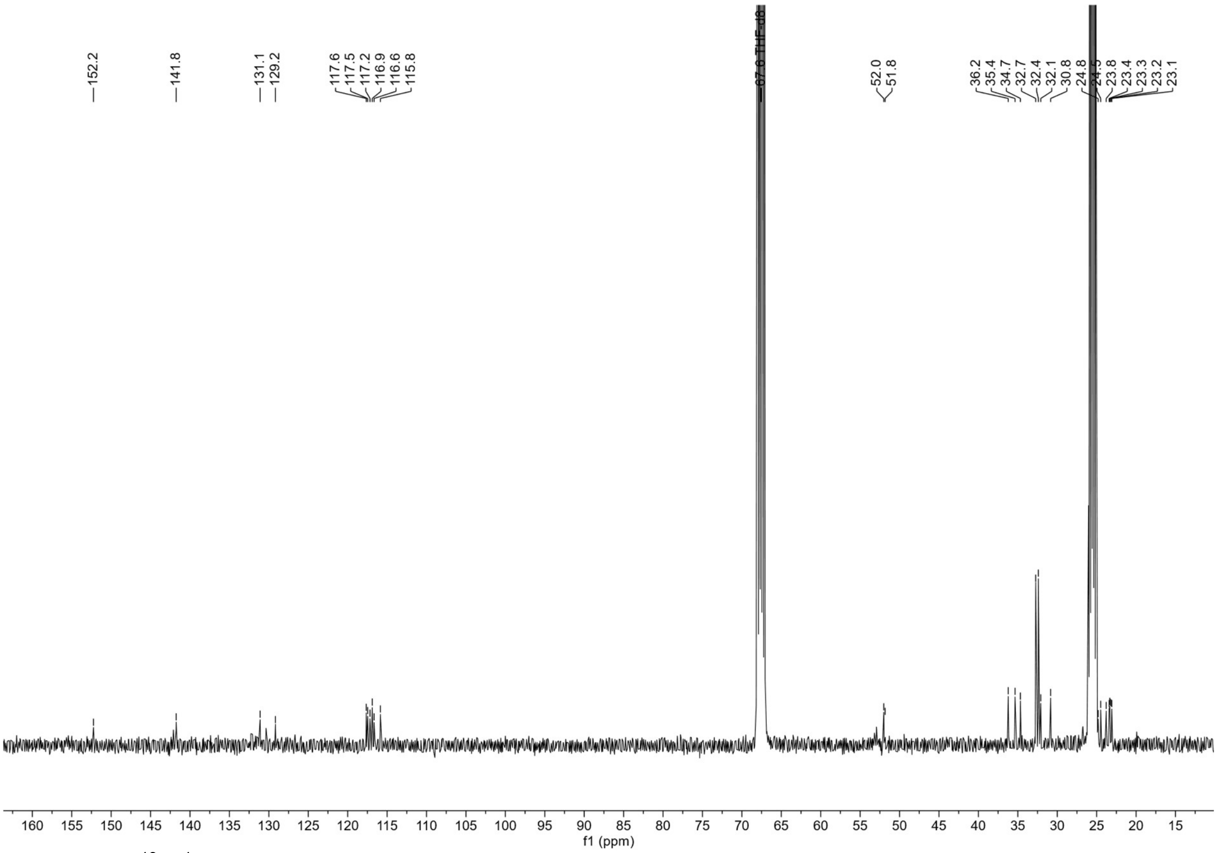

Figure S23. $^{13}C\{^{1}H\}$ NMR spectrum (101 MHz, C_6D_6, 298 K) of **2b**.

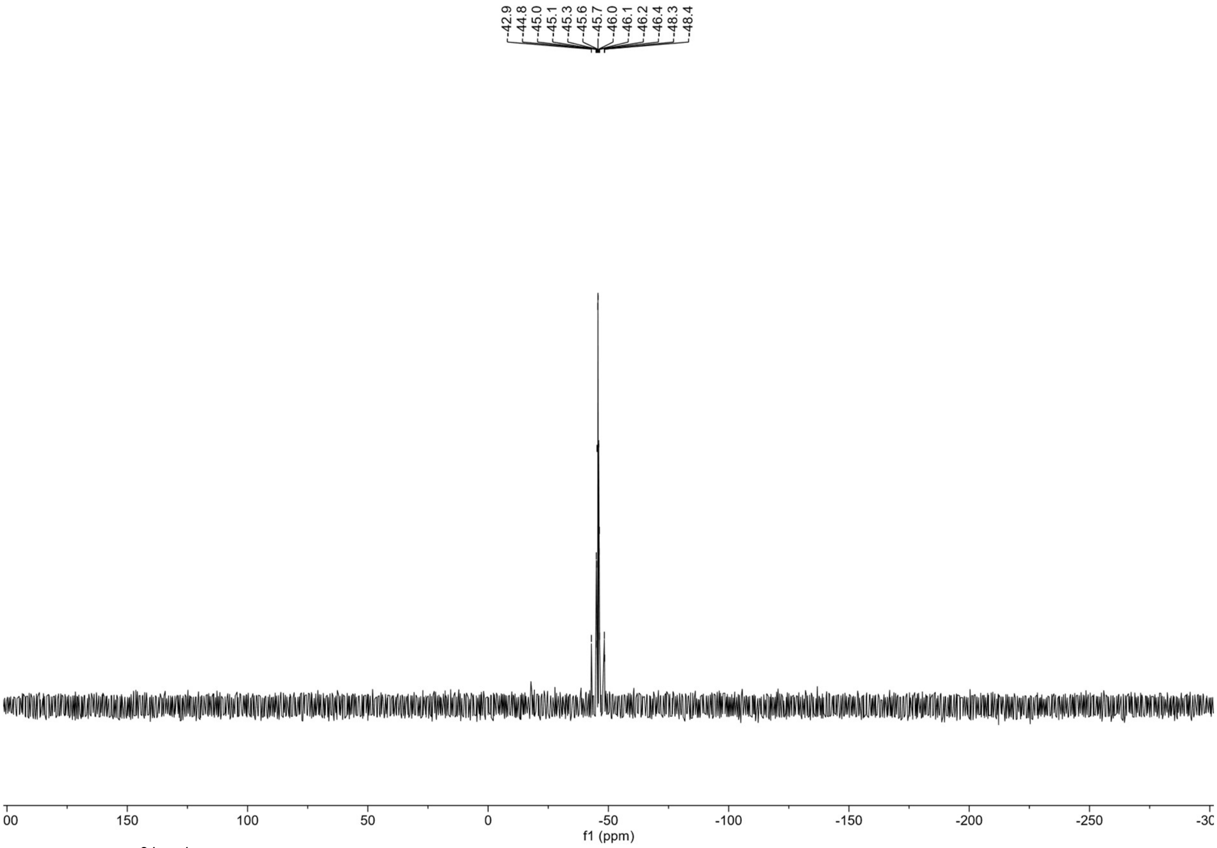

Figure S24. $^{31}P\{^{1}H\}$ NMR spectrum (162 MHz, THF-d_8, 298 K) of **2b**.

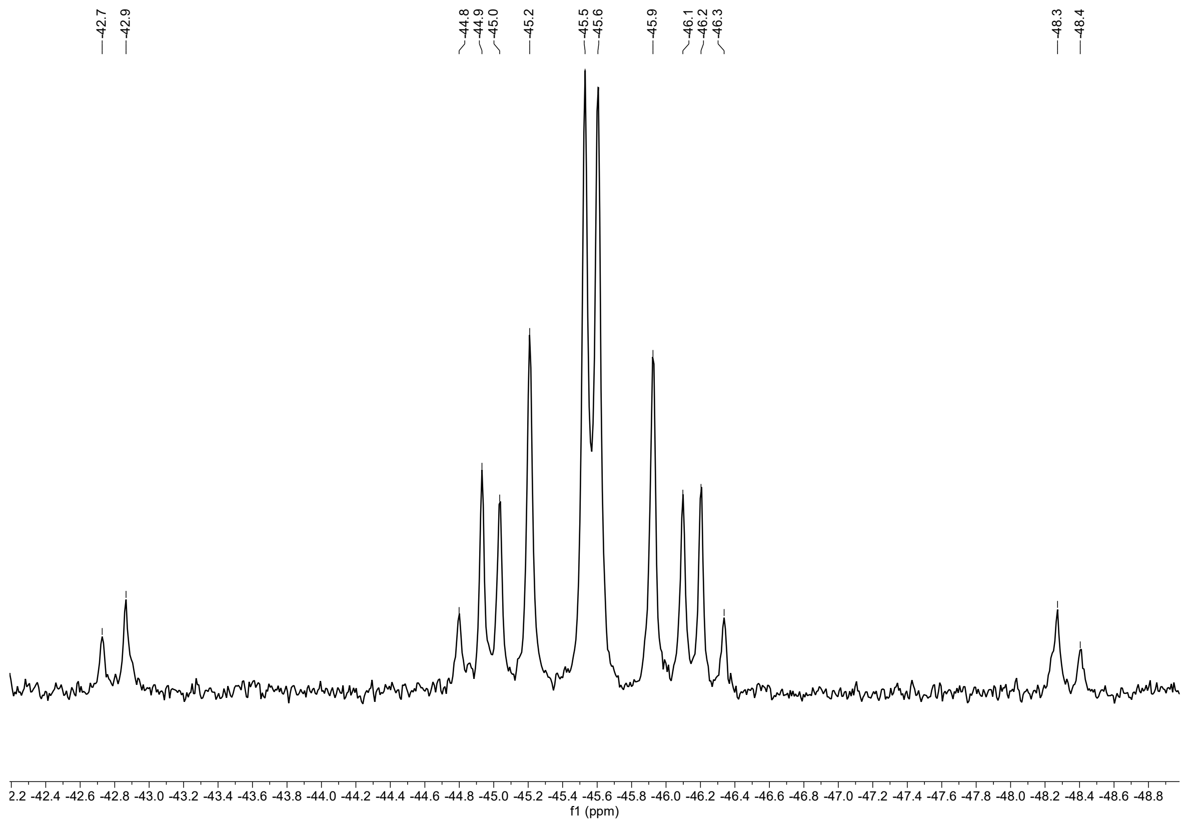

Figure S25. $^{31}P\{^{1}H\}$ NMR spectrum (162 MHz, THF-d_8, 298 K) of **2b** in the region from -42 to -49 ppm.

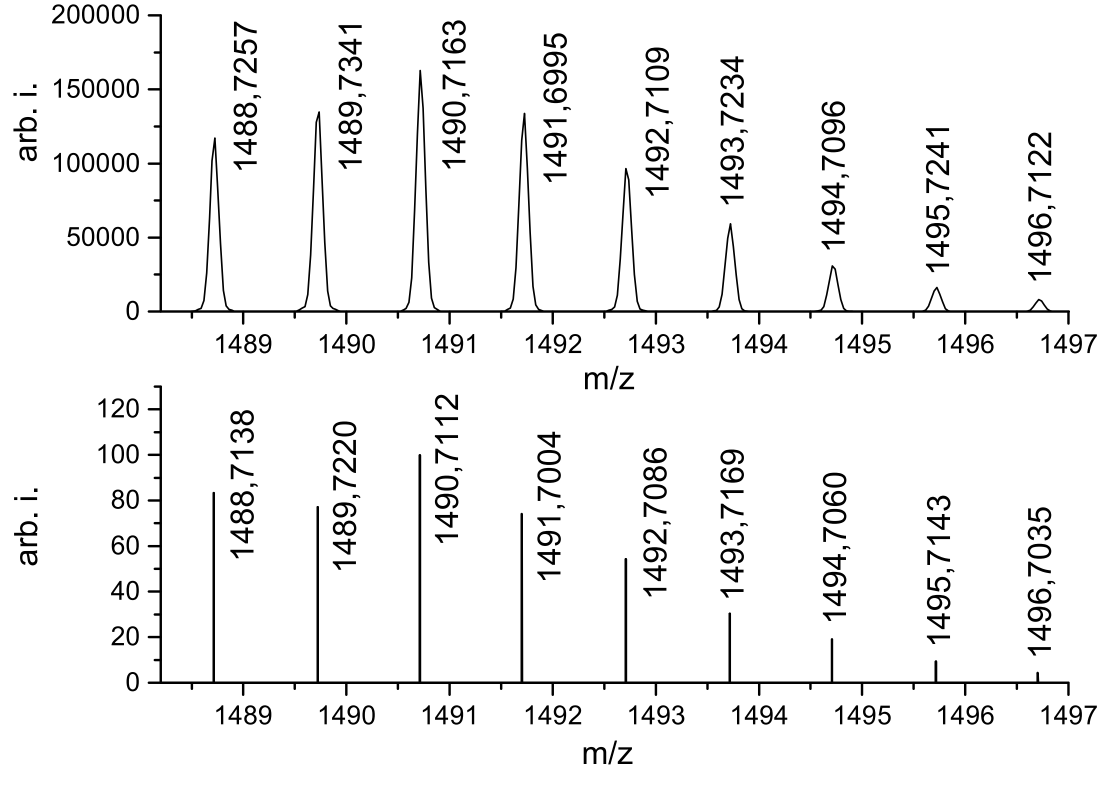

Figure S26. Cutout from LIFDI/MS of **2b**, Top: found MS for $[^{tBu}XantP_2(Ni(NHC)_2)_2]^+$; Bottom: Calculated MS spectrum of $[^{tBu}XantP_2(Ni(NHC)_2)_2]^+$.

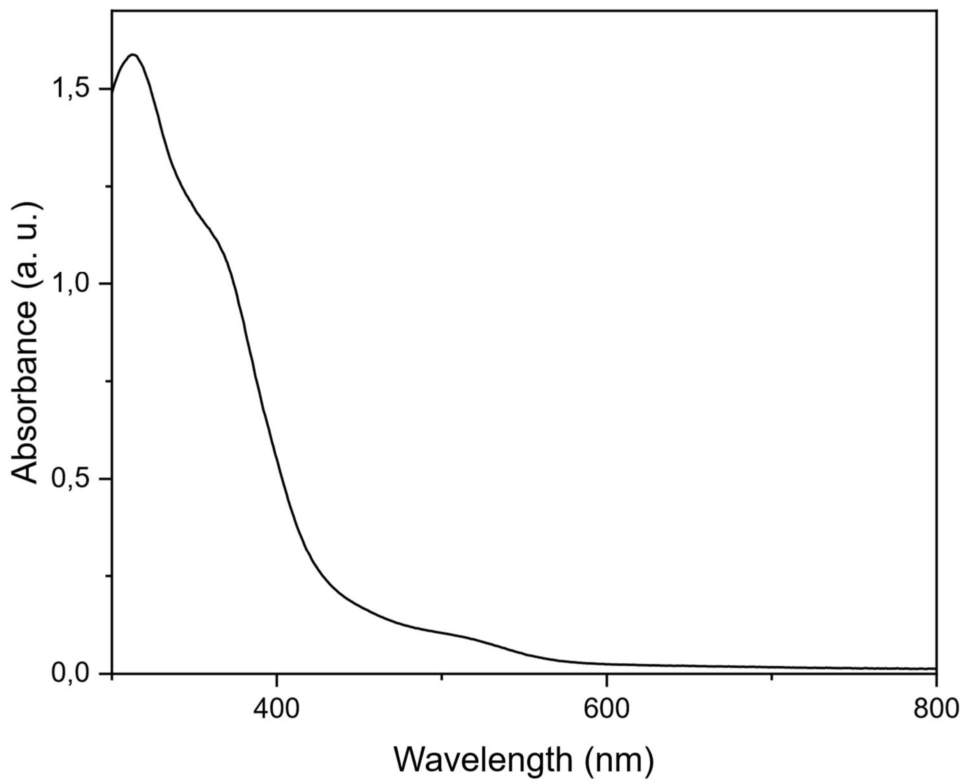

Figure S27. UV/Vis spectrum of a 4.69x10^{-5} M solution of **2b** in THF at ambient temperature.

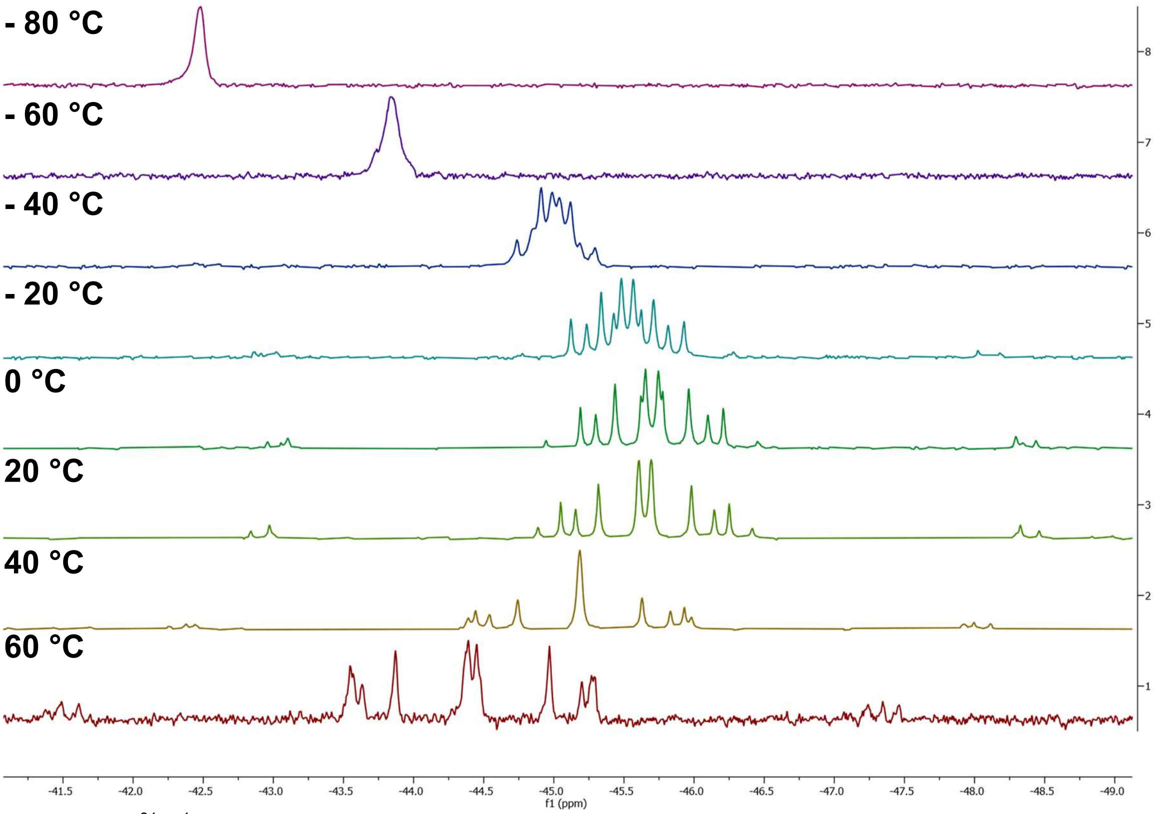

Figure S28. $^{31}P\{^{1}H\}$ VT-NMR (162 MHz, THF-d_8) of **2b**.

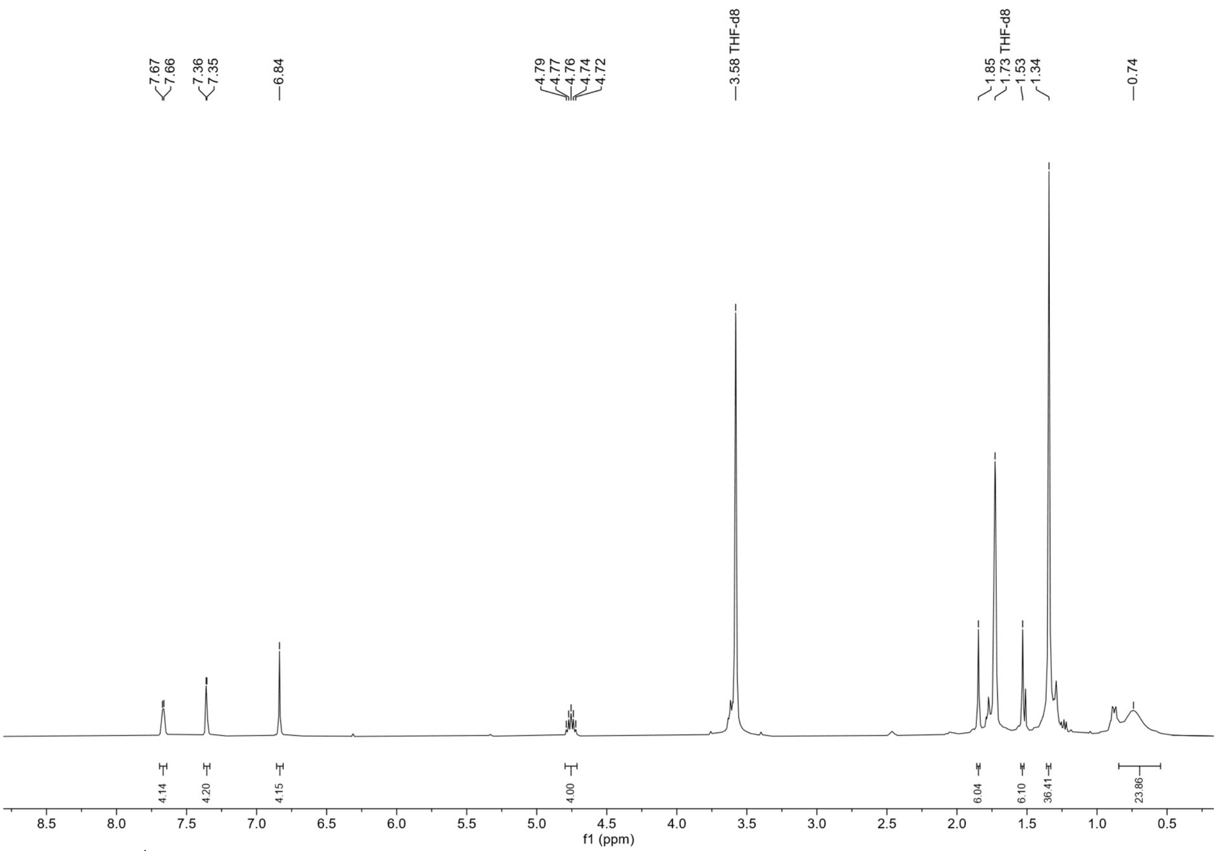

Figure S29. ^{1}H NMR spectrum (400 MHz, THF-d_8, 298 K) of **3**.

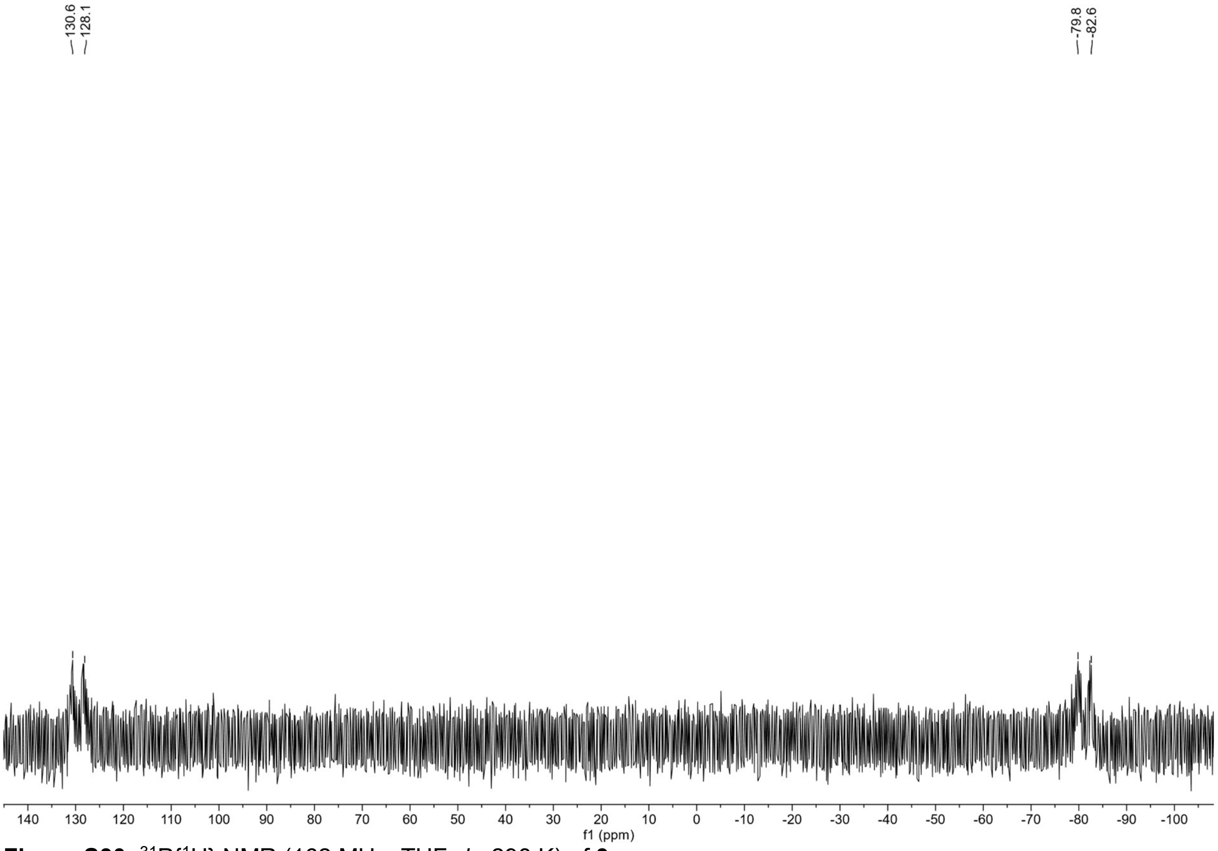

Figure S30. $^{31}P\{^{1}H\}$ NMR (162 MHz, THF-d_8, 298 K) of **3**.

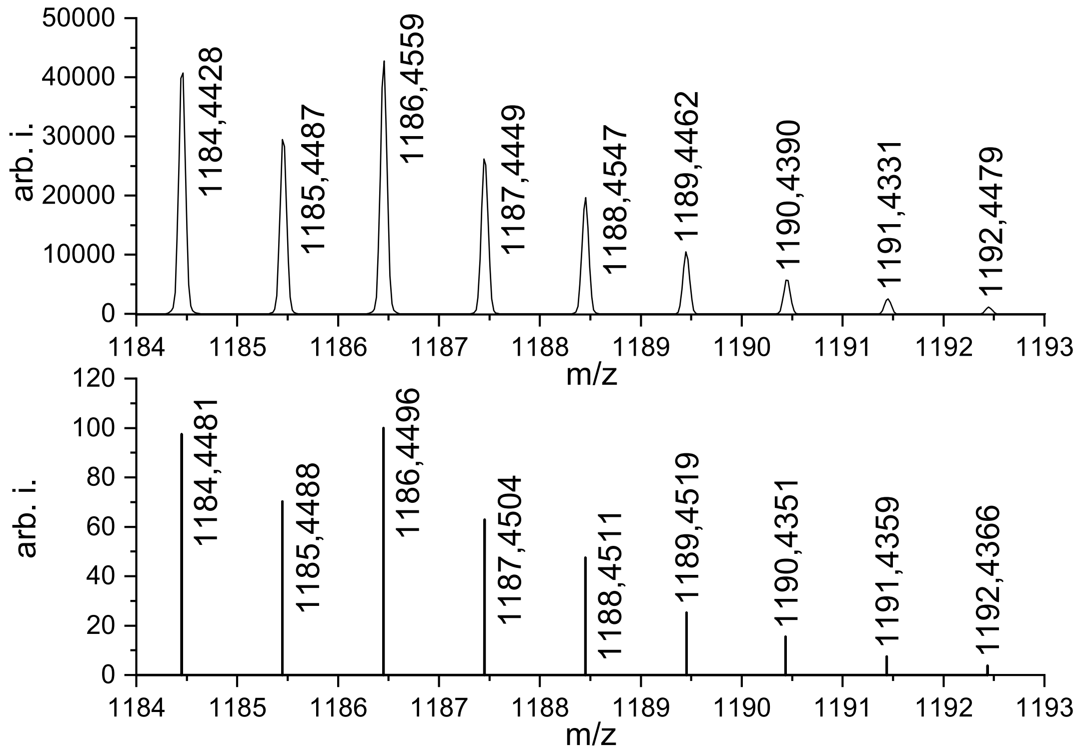

Figure S31. Cutout from LIFDI/MS of **3**, Top: found MS for $[^{tBu}XantP_2(NiNHC)_2]^+$; Bottom: Calculated MS spectrum of $[^{tBu}XantP_2(NiNHC)_2]^+$.

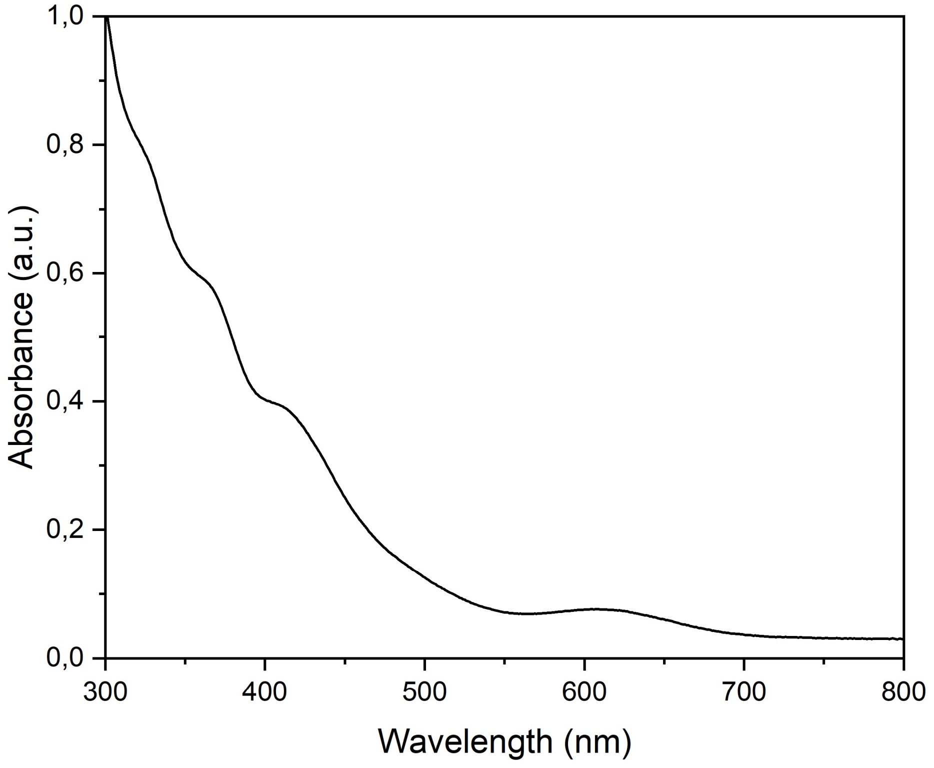

Figure S32. UV/Vis spectrum of a $6.32x10^{-5}$ M solution of **3** in THF at ambient temperature.

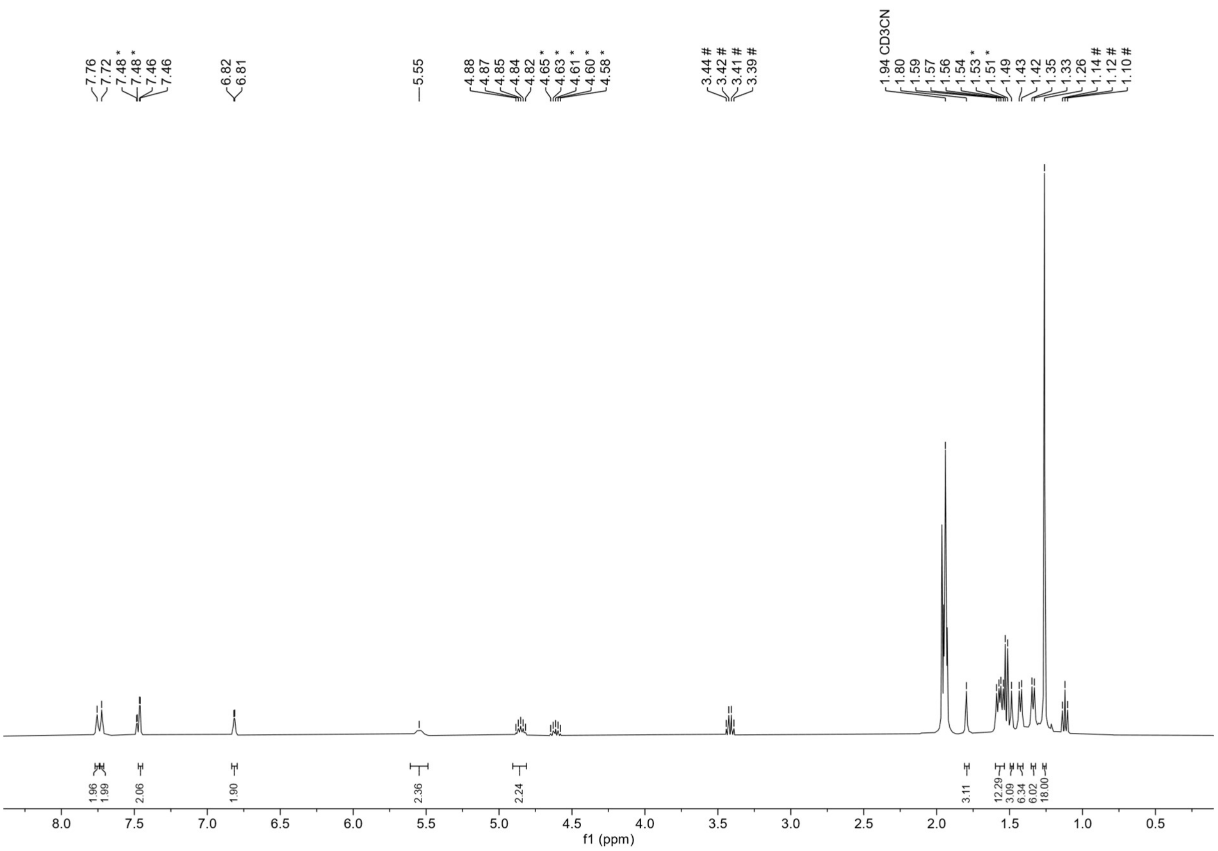

Figure S33. ^{1}H NMR spectrum (400 MHz, CD_3CN, 298 K) of **1b**·$GeCl_2$. # denotes minor amounts of diethylether and * denotes minor amounts of NHC-byproduct.

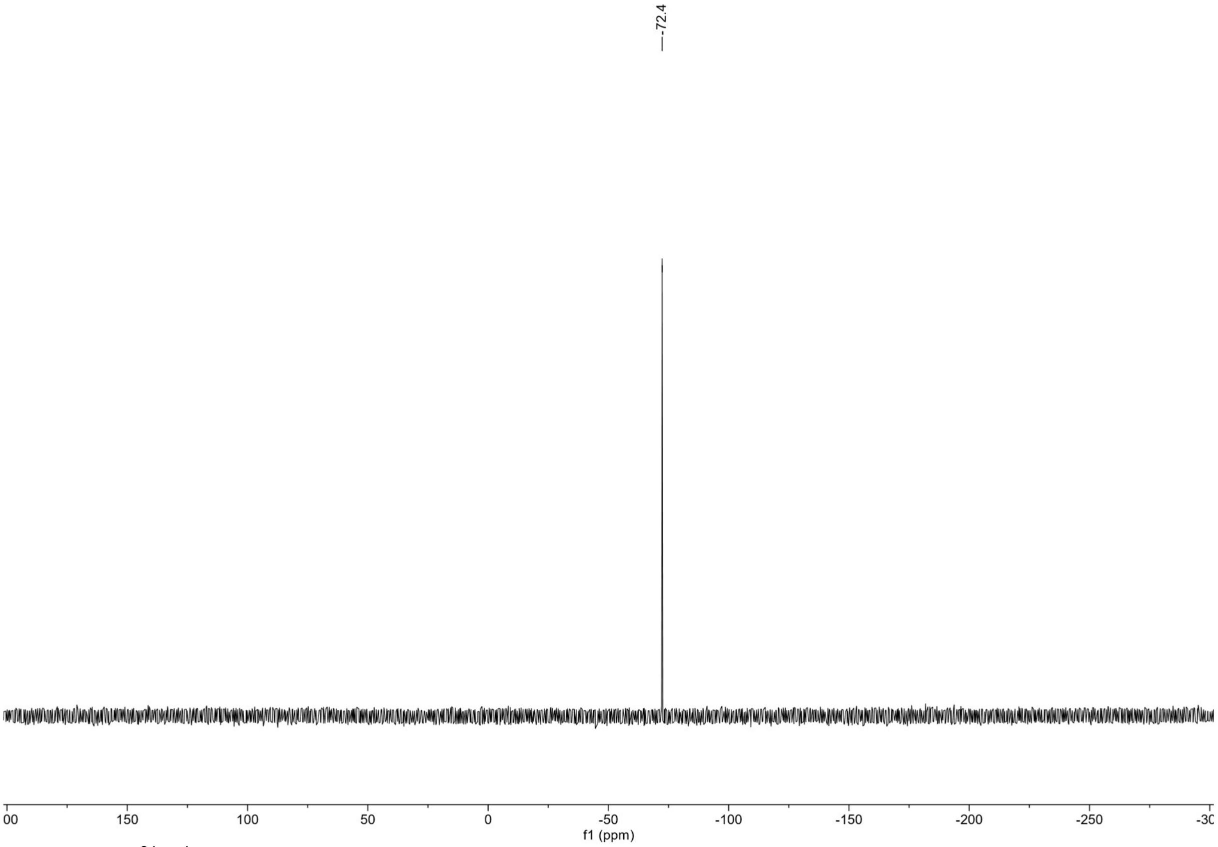

Figure S34. $^{31}P\{^1H\}$ NMR (162 MHz, CD_3CN, 298 K) of **1b**·$GeCl_2$.

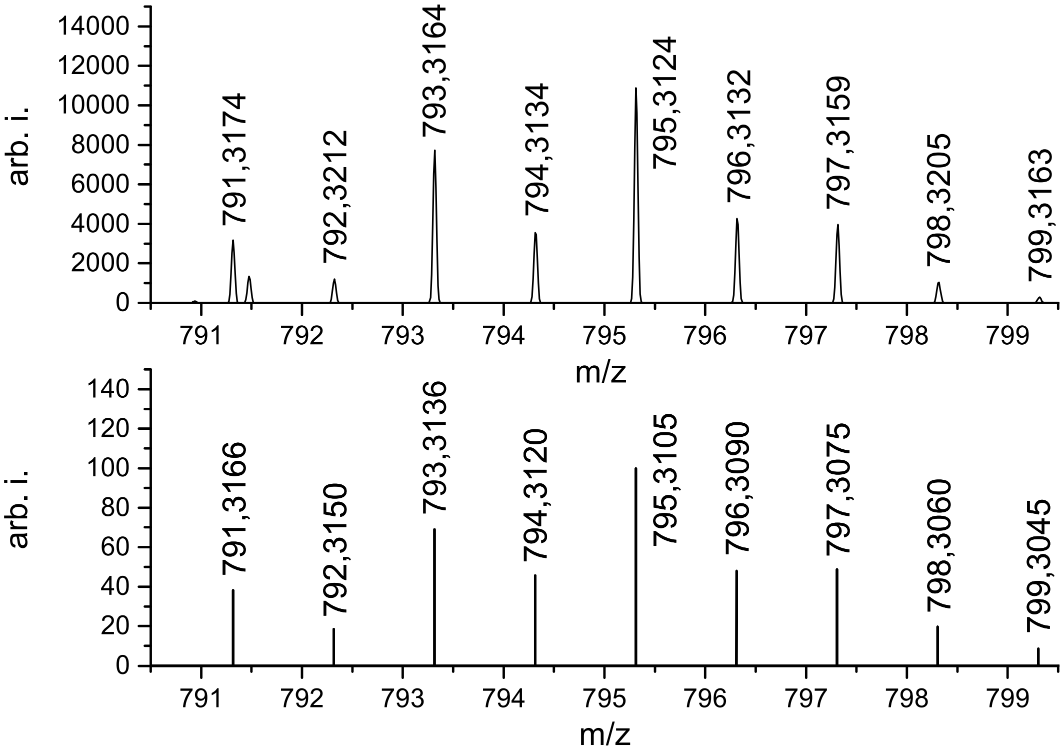

Figure S35. Cutout from LIFDI/MS of **1b**·$GeCl_2$, Top: found MS for $[^{tBu}XantP_2{\bullet}GeCl]^+$; Bottom: Calculated MS spectrum of $[^{tBu}XantP_2{\bullet}GeCl]^+$.

Simulations of $^{31}P\{^1H\}$ VT-NMR spectra of 2b

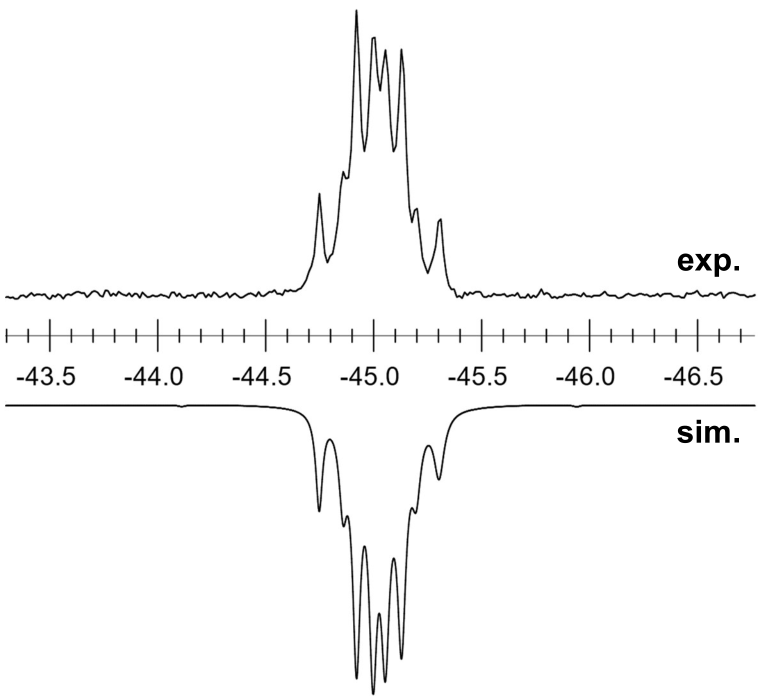

Figure S36. Top: Experimental $^{31}P\{^1H\}$ NMR (162 MHz, THF-d_8) of **2b** at - 40 °C. Bottom: Simulated $^{31}P\{^1H\}$ NMR spectrum of **2b** at - 40 °C

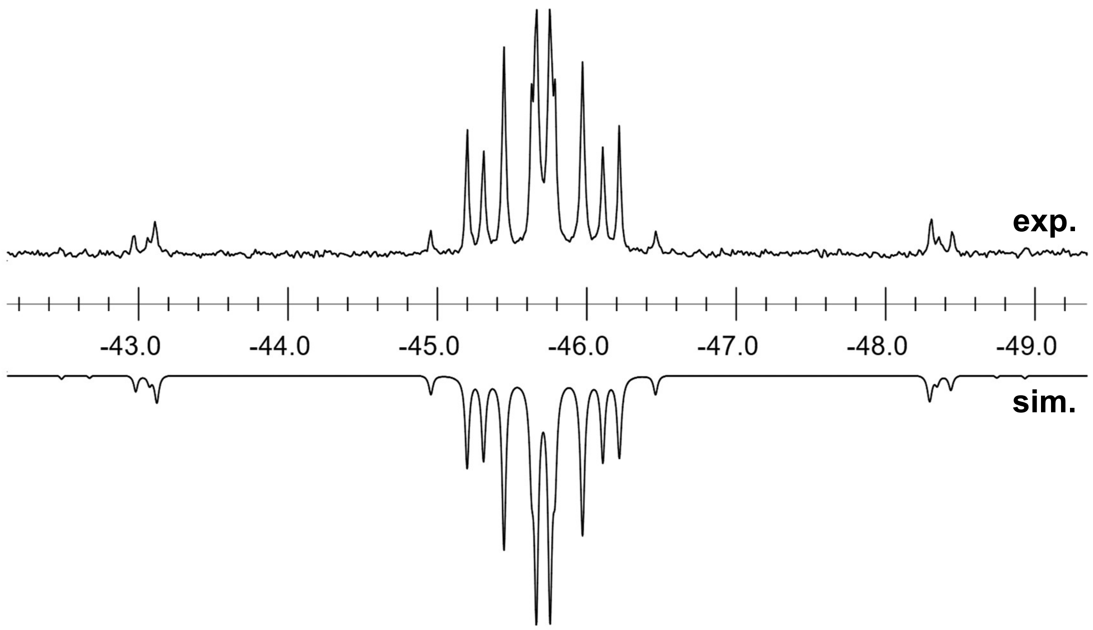

Figure S37. Top: Experimental $^{31}P\{^1H\}$ NMR (162 MHz, THF-d_8) of **2b** at 0 °C. Bottom: Simulated $^{31}P\{^1H\}$ NMR spectrum of **2b** at 0 °C

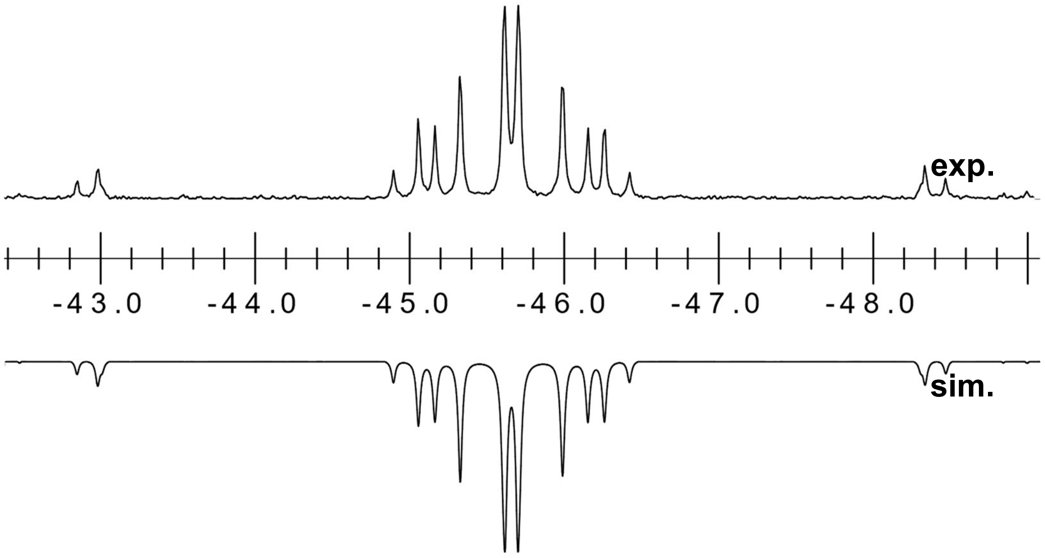

Figure S38. Top: Experimental $^{31}P\{^1H\}$ NMR (162 MHz, THF-d_8) of **2b** at 20 °C. Bottom: Simulated $^{31}P\{^1H\}$ NMR spectrum of **2b** at 20 °C

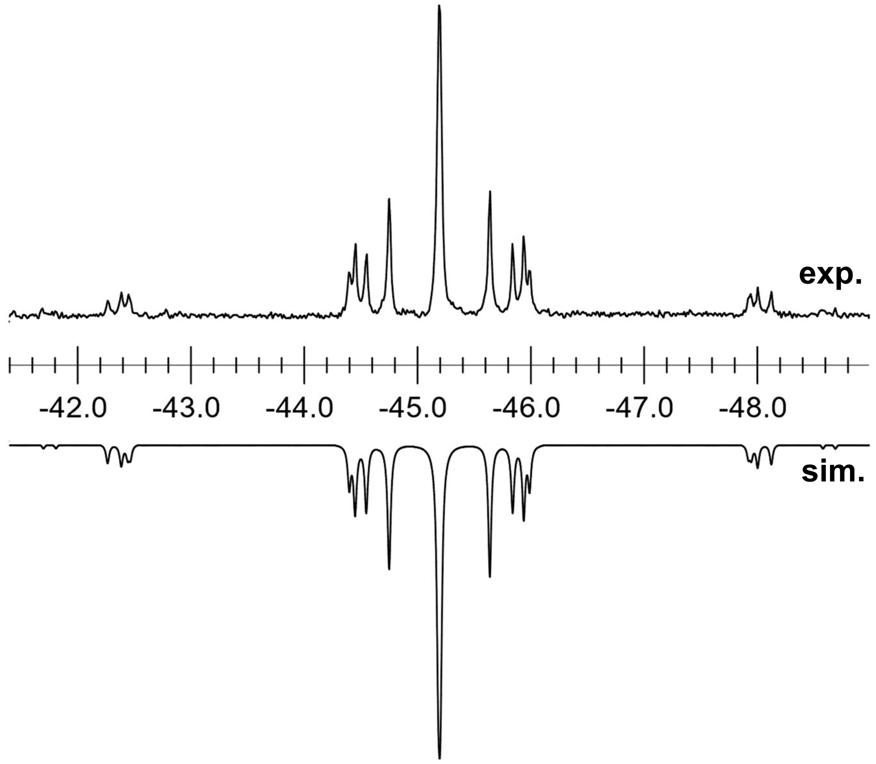

Figure S39. Top: Experimental $^{31}P\{^1H\}$ NMR (162 MHz, THF-d_8) of **2b** at 40 °C. Bottom: Simulated $^{31}P\{^1H\}$ NMR spectrum of **2b** at 40 °C

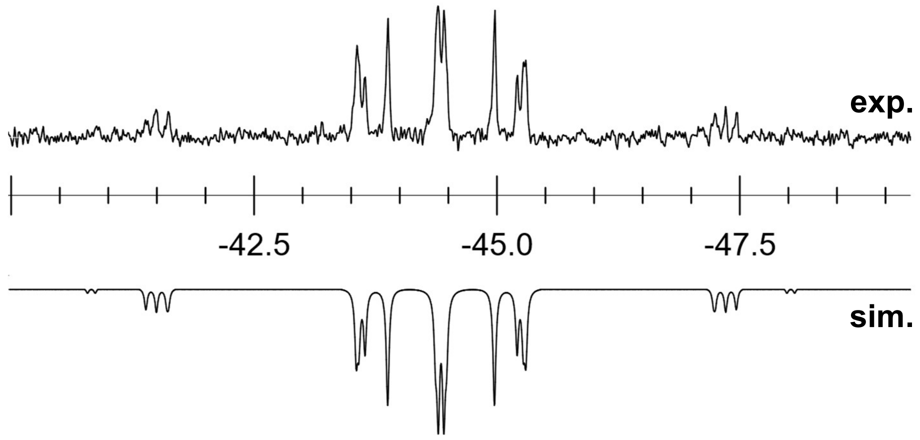

Figure S40. Top: Experimental $^{31}P\{^1H\}$ NMR (162 MHz, THF-d_8) of **2b** at 60 °C. Bottom: Simulated $^{31}P\{^1H\}$ NMR spectrum of **2b** at 60 °C

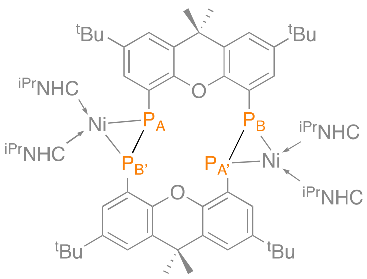

Table S1. Simulated shifts (A, B in ppm), widths (wA, wB in ppm) and coupling constants (AB, A'B', AB', A'B, AA', BB' in Hz)

[°C]	A	wA	B	wB	AB	A'B'	AB'	A'B	AA'	BB'
**60**	-45,72	5,67	-43,13	5,63	-404,7	-404,7	1,47	1,47	188,53	-0,62
**40**	-46,34	4,99	-44,05	5,43	-401,3	-406,5	9,71	-6,4	183,35	-1,03
**20**	-46,62	5,23	-44,7	4,81	-403,1	-403,1	1,4	1,4	179,78	-1,03
**0**	-46,56	5,65	-44,86	4,82	-403,6	-398,0	9,56	-6,1	175,42	-1,31
**-40**	-45,49	9,89	-44,56	5,35	-372,2	-368,2	55,3	45,2	174,33	-65,16

2. X-ray crystallographic details

Single crystals of **1b**, **1c**, **2a**, **2b**, **2b**', **3**, and **4** suitable for X-ray structural analysis were mounted in perfluoroalkyl ether oil on a nylon loop and positioned in a 150 K cold N_2 gas stream. Data collection was performed with a STOE StadiVari diffractometer (MoKα radiation) equipped with a DECTRIS PILATUS 300K detector. Structures were solved by Direct Methods (SHELXS-97)[6] and refined by full-matrix least-squares calculations against F^2 (SHELXL-2018).[6] The positions of the hydrogen atoms were calculated and refined using a riding model. All non-hydrogen atoms were treated with anisotropic displacement parameters. Crystal data, details of data collections, and refinements for all structures can be found in their CIF files, which are available free of charge via www.ccdc.cam.ac.uk/data_request/cif, and are summarized in Tables S1 and S2. In compounds **1b**, **1c**, and **4** the electron density of highly disordered co-crystallized solvent molecules was removed using the PLATON SQUEEZE function.[7]

Response to alerts in CheckCif files (A and B alerts only):

Compound **1c**:

Alert Level B: `PLAT910_ALERT_3_B Missing # of FCF Reflection(s) Below Theta(Min). 13 Note`

Given that the error values and completeness for this data are well within the accepted levels, this alert is not detrimental to this molecular structure.

Compound **2b'**:

Alert Level A: `PLAT602_ALERT_2_A Solvent Accessible VOID(S) in Structure`

No solvent was SQUEEZed from this structure, and thus this is presumably a packing effect.

Compound **4**:

Alert Level A: `PLAT213_ALERT_2_A Atom C21 has ADP max/min Ratio ..... 5.7 prolat`

This is due to significant disorder in one *tert*-butyl group of the ^{tBu}Xant ligand, which is a known effect. This has been modelled, but still leads to the above alert. Nevertheless, the overall completeness of this data, and the given error values are still well within those for publishable data.

Compound **1b·GeCl₂**:

Alert Level A: `PLAT213_ALERT_2_A Atom C22 has ADP max/min Ratio ..... 5.1 prolat`

As above, this is due to a highly disordered *tert*-butyl group of the ^{tBu}Xant ligand. The overall completeness of this data, and the given error values are still well within those for publishable data.

Alert Level B: `PLAT220_ALERT_2_B NonSolvent Resd 1 C Ueq(max)/Ueq(min) Range 6.2 Ratio`

This is directly related to the above described disorder.

Alert Level B: `PLAT910_ALERT_3_B Missing # of FCF Reflection(s) Below Theta(Min). 11 Note`

Given that the error values and completeness for this data are well within the accepted levels, this alert is not detrimental to this molecular structure.

Table S2. Summary of X-ray crystallographic data for **1b**, **1c**, **2a**, and **2b**.

	**1b**	**1c**	**2a**	**2b**
empirical form.	$C_{41}H_{60}N_4OP_2$	$C_{30}H_{38}N_4OP_2$	$C_{66}H_{88}N_8Ni_2O_2P_4$	$C_{82}H_{120}N_8Ni_2O_2P_4$
formula wt	686.87	532.58	1266.74	1491.15
crystal syst.	monoclinic	monoclinic	monoclinic	triclinic
space group	$P2_1/n$	$P2_1/n$	$P2_1/n$	*P-1*
a (Å)	18.707(4)	10.840(2)	9.890(2)	10.760(2)
b (Å)	15.187(3)	23.820(5)	21.590(4)	12.310(3)
c (Å)	31.540(6)	26.540(5)	15.520(3)	15.630(3)
α (deg.)	90	90	90	97.50(3)
β (deg.)	99.45(3)	100.20(3)	93.10(3)	99.40(3)
γ (deg.)	90	90	90	96.00(3)
vol ($Å^3$)	8839(3)	6745(2)	3309.1(12)	2008.2(7)
Z	8	8	2	1
ρ(calc) ($g.cm^{-3}$)	1.032	1.049	1.271	1.233
μ (mm^{-1})	0.130	0.154	0.714	0.599
F(000)	2976	2272	1344	800
T (K)	150(2)	150(2)	150(2)	150(2)
reflns collect.	66457	50683	31963	20319
unique reflns	17265	13267	6499	7853
R_{int}	0.0636	0.1505	0.0949	0.0411
R1 [$I>2\sigma(I)$]	0.0561	0.0764	0.0518	0.0375
*w*R2 (all data)	0.1088	0.1688	0.1306	0.0684
CCDC No.	2357717	2357716	2357713	2357711

Table S3. Summary of X-ray crystallographic data for **2b'**, **3**, **4**, and **1b·GeCl$_2$**.

	**2b'**	**3**	**4**	**1b·GeCl$_2$**
empirical form.	$C_{82}H_{120}N_8Ni_2O_4P_4\cdot(THF)_2$	$C_{72}H_{108}N_4Ni_2O_4P_4$	$C_{82}H_{120}Cl_2Cu_2N_8Ni_2O_2P_4$	$C_{41}H_{60}Cl_2GeN_4O_1P_2\cdot0.5(Et_2O)(Hex)\cdot2(CH_3CN)$
formula wt	1,635.36	1334.92	1689.13	992.61
crystal syst.	monoclinic	triclinic	tetragonal	orthorhombic
space group	*P2₁/c*	*P-1*	*I4₁/a*	*Pccn*
a (Å)	15.304(3)	9.912(2)	35.655(5)	17.370(4)
b (Å)	34.054(7)	14.075(3)	35.655(5)	18.920(4)
c (Å)	9.7484(19)	15.094(3)	15.130(3)	33.750(7)
α (deg.)	90	66.91(3)	90	90
β (deg.)	97.35(3)	70.89(3)	90	90
γ (deg.)	90	76.79(3)	90	90
vol (Å^3)	5038.9(18)	1818.3(8)	19234(7)	11092(4)
Z	2	1	8	8
ρ(calc) (g.cm^{-3})	1.078	1.219	1.167	1.189
μ (mm^{-1})	0.484	0.654	0.987	0.746
F(000)	1760	716	7136	4224
T (K)	150(2)	150(2)	150(2)	150(2)
reflns collect.	38479	24420	36349	36330
unique reflns	9847	7148	9424	10849
R_{int}	0.1622	0.0886	0.1066	0.0661
R1 [$I>2\sigma(I)$]	0.0772	0.0644	0.0635	0.0792
*w*R2 (all data)	0.2087	0.1592	0.1511	0.1609
CCDC No.	2357714	2357712	2357715	2357710

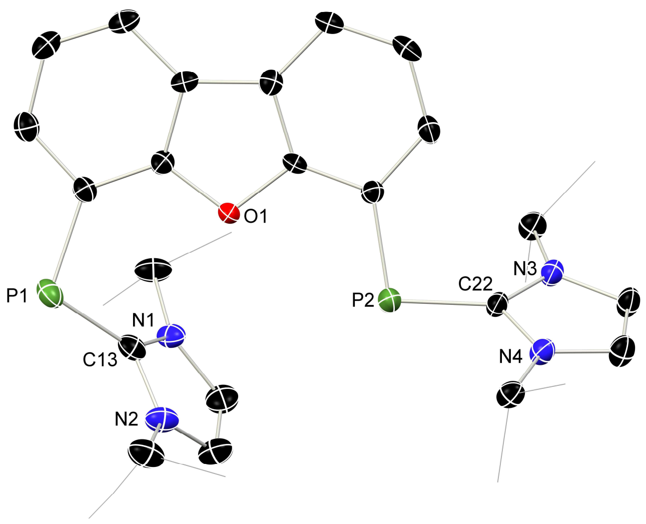

Figure S41. The molecular structure of **1c**, with thermal ellipsoids at 30% probability, and hydrogen atoms omitted for clarity.

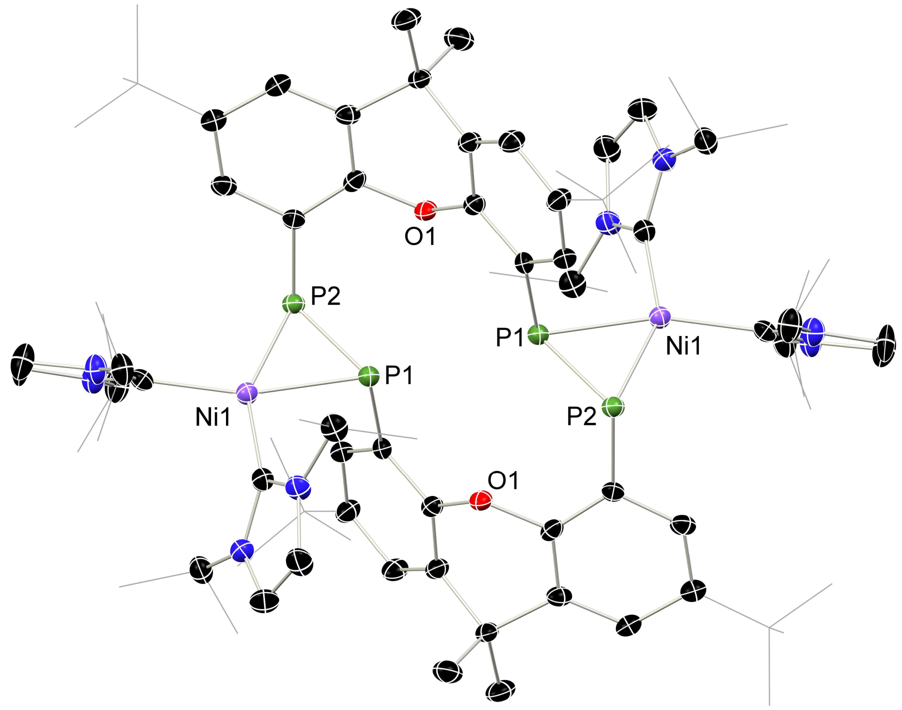

Figure S42. The molecular structure of **2b'**, with thermal ellipsoids at 30% probability, and hydrogen atoms omitted for clarity.

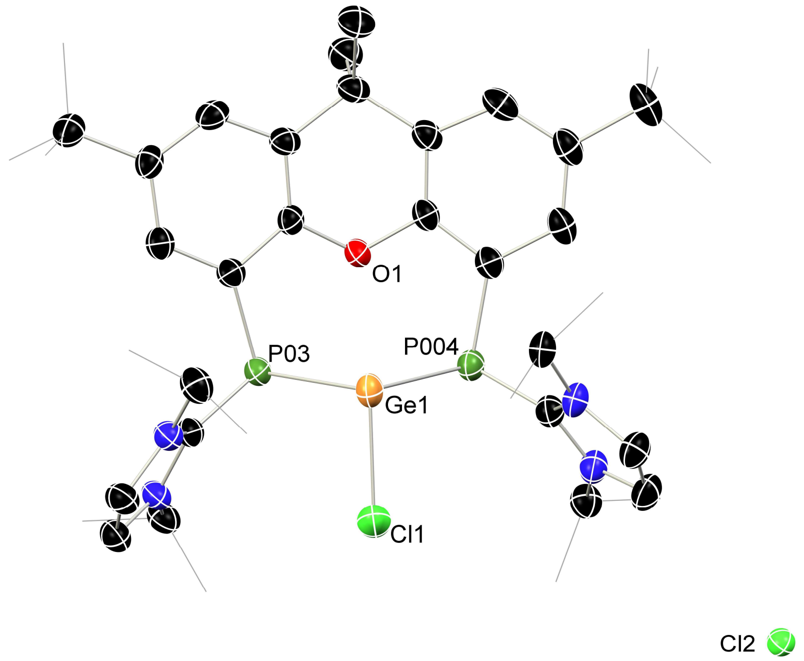

Figure S43. The molecular structure of **1b·GeCl₂**, with thermal ellipsoids at 30% probability, and hydrogen atoms omitted for clarity.

3. Computational methods and details

Computational experiments were performed using the Gaussian 16 program.[8] Geometry optimization was carried out on the full molecule of **2a**, and on **2b'** and **3** with *tBu* groups removed, at the BP86/def2SVP level of theory.[9],[10],[11],[12] Single point, TD-DFT, and NBO calculations were then carried out at the B97D3/def2TZVPP level of theory. Solvent effects were considered using the SMD model, for THF and benzene, for all calculations. Stationary points were confirmed as true minima by vibrational frequency analysis (no negative eigenvalues). Dispersion corrections were implemented with Grimme's D3 model for all calculations.[12] The QTAIM analysis for **2b'** was executed using the Multiwfn program.[13],[14],[15] An NBO analysis for **2b'** and **3** was carried out using the NBO 6 program,[16] using geometries determined through the above described methods. NLMOs are visualised using ChemCraft.

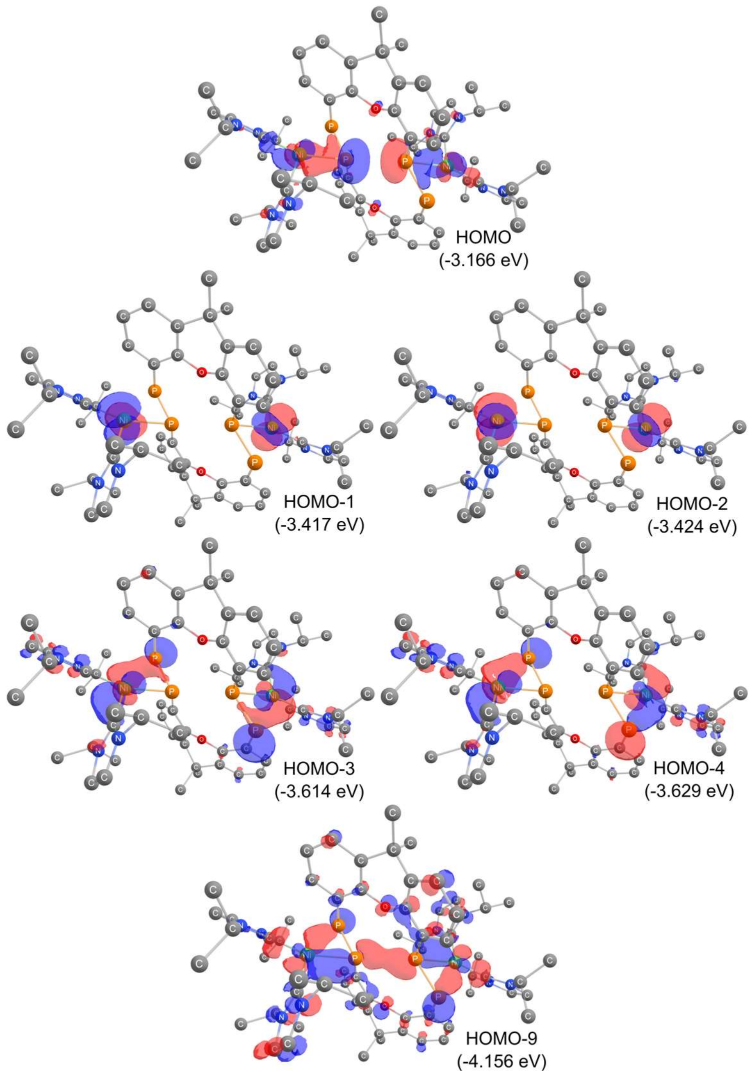

Figure S44. Graphical representations of the highest occupied Kohn-Sham-Orbitals, at the B97D3/def2SVP level of theory, for the full molecule of **2a**.

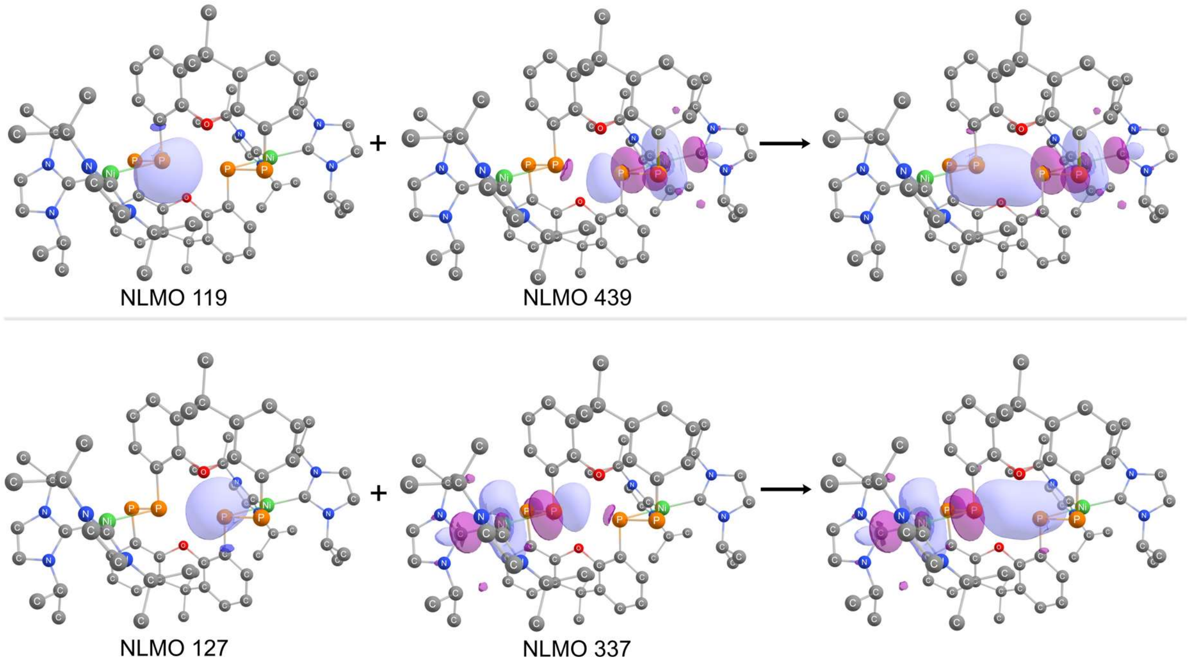

Figure S45. Orbital interactions considered as part of the P1-P1’ bond in **2b’** shown in the main text, through a second-order perturbation theory analysis at the B97D3/def2SVP level of theory.

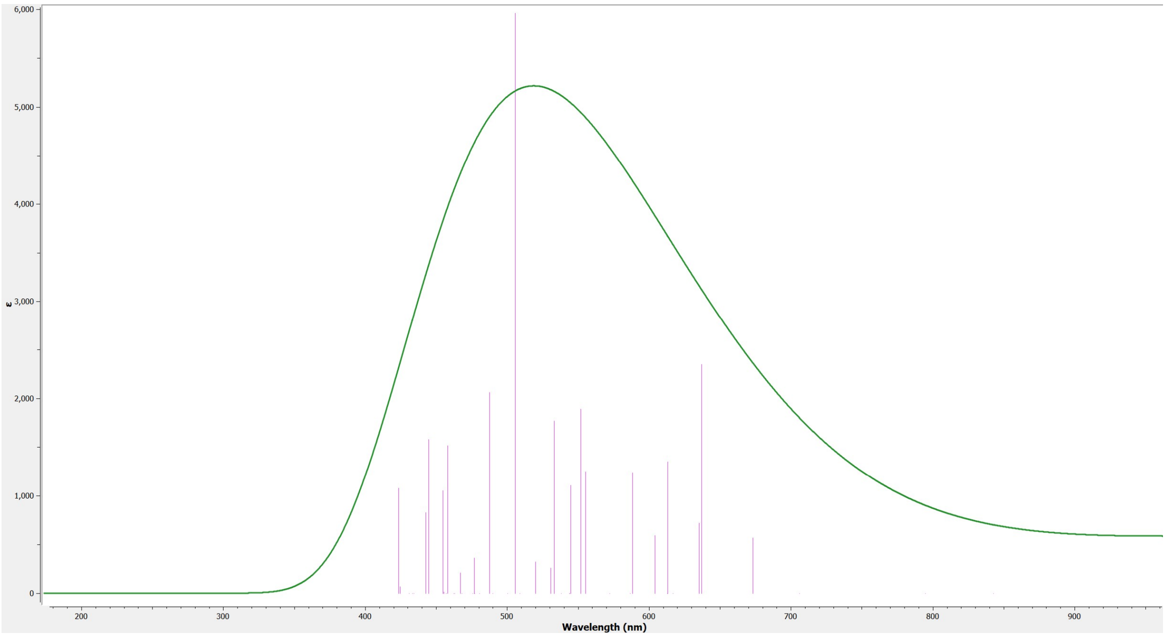

Figure S46. Calculated UV/vis spectrum for the full molecule of **3**.

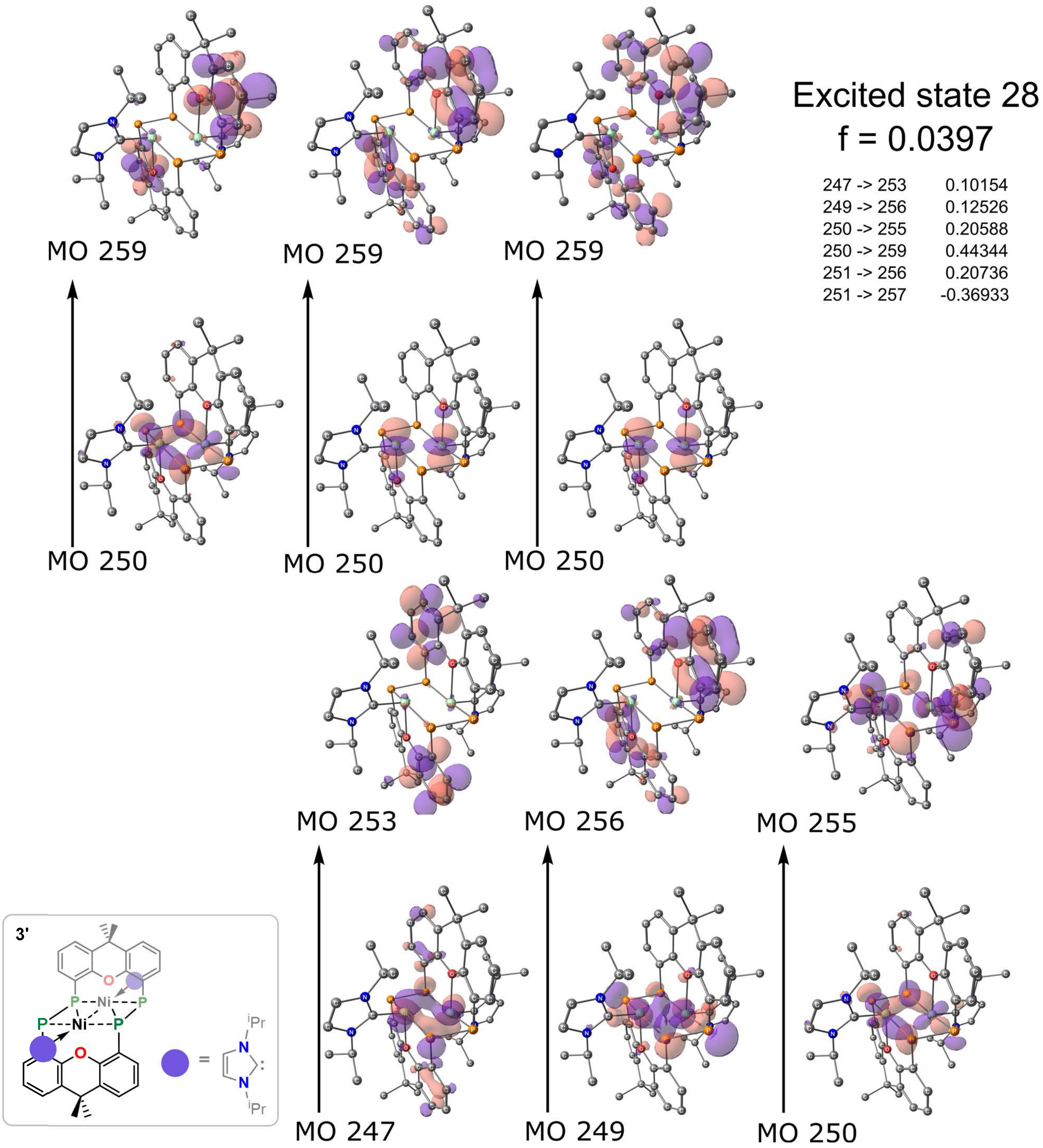

Figure S47. MO transitions involved in the strongest transition in the TD-DFT analysis of **3'**, excited state 28.

Table S4. Cartesian coordinates for **2b'** at the B97D3/def2TZVPP level of theory.

Ni	3.73755	0.10565	0.09188
P	1.48801	0.28383	0.28313
P	-2.555	0.70707	-1.89755
O	-0.24205	2.5514	-0.67485
N	4.38629	2.12896	-1.92459
N	6.29259	-1.34206	-0.15868
N	3.26147	0.49851	-2.81131

N	6.29345	-0.04245	1.57512
C	1.40811	2.01545	0.98765
C	0.57416	2.97674	0.36123
C	-2.55966	2.47891	-1.33044
C	-1.44764	3.21791	-0.85104
C	2.16123	2.46775	2.1009
H	2.78785	1.74025	2.64218
C	-1.52475	4.59952	-0.55177
C	1.31812	4.73587	1.8576
H	1.31471	5.78591	2.18364
C	-3.77058	3.19591	-1.50849
H	-4.65224	2.64554	-1.875
C	3.78894	0.92705	-1.60958
C	5.50493	-0.42682	0.50925
C	-3.87481	4.55939	-1.2048
C	0.55437	4.34085	0.74457
C	-2.75646	5.25933	-0.71849
H	-2.84237	6.33134	-0.49007
C	-0.22248	5.30669	-0.1571
C	2.10452	3.79516	2.54592
C	5.00256	2.98562	-0.89862
H	4.99189	2.33732	0.00559
C	0.95722	-0.28206	-3.26316
H	0.59053	0.45438	-2.52134
H	0.28602	-1.16305	-3.21084
H	0.87276	0.15926	-4.27952
C	4.2277	2.44448	-3.27252
C	3.51858	1.41118	-3.83121
C	7.52452	-1.51618	0.46694
C	5.89858	-1.89914	-1.46496
H	4.79623	-1.76269	-1.48099
C	5.84604	0.93112	2.58521
H	4.84443	1.23808	2.21449
C	2.39861	-0.69575	-2.94892
H	2.41963	-1.15414	-1.93662
C	-0.4713	6.6672	0.51441
H	-1.00426	7.35417	-0.17302
H	0.48792	7.1601	0.77114
H	-1.07169	6.56315	1.4412
C	7.5226	-0.69756	1.5687
C	0.61798	5.53192	-1.45159
H	0.83505	4.56943	-1.95687
H	1.58522	6.01601	-1.20145
H	0.0627	6.17989	-2.16172
C	2.97046	-1.69081	-3.96492
H	2.99088	-1.26364	-4.99037
H	2.33418	-2.59826	-3.99229
H	4.00079	-1.99859	-3.69848
C	6.76765	2.15598	2.6103

H	7.78414	1.89886	2.97785
H	6.3516	2.93281	3.28343
H	6.86775	2.59107	1.59569
C	6.51782	-1.05603	-2.5888
H	6.20455	0.00237	-2.48292
H	6.1862	-1.41773	-3.58321
H	7.62708	-1.10648	-2.55608
C	4.12798	4.2131	-0.62331
H	4.07072	4.8778	-1.5118
H	4.53958	4.80028	0.22235
H	3.1009	3.89982	-0.35113
C	6.45397	3.31995	-1.25803
H	7.04528	2.39219	-1.39936
H	6.92202	3.91327	-0.446
H	6.52017	3.9237	-2.18822
C	6.2121	-3.39269	-1.56973
H	7.30521	-3.5919	-1.57411
H	5.80053	-3.79196	-2.51885
H	5.7446	-3.94759	-0.73242
C	5.67136	0.26359	3.953
H	4.9508	-0.57522	3.87314
H	5.26955	0.99317	4.68554
H	6.63679	-0.12077	4.34653
P	-1.4881	-0.28369	-0.28281
P	2.55504	-0.70686	1.89785
O	0.24207	-2.55124	0.67512
C	-1.4081	-2.01529	-0.98738
C	-0.57411	-2.97656	-0.36099
C	2.55969	-2.4787	1.33072
C	1.44767	-3.21772	0.85132
C	-2.16119	-2.46759	-2.10066
H	-2.78787	-1.74011	-2.64189
C	1.52482	-4.59931	0.55201
C	-1.31791	-4.73565	-1.8575
H	-1.31441	-5.78567	-2.18361
C	3.77062	-3.19569	1.50876
H	4.65227	-2.64528	1.87524
C	3.87487	-4.55916	1.20507
C	-0.55425	-4.34066	-0.7444
C	2.75654	-5.2591	0.71874
H	2.84247	-6.33111	0.4903
C	0.22259	-5.30651	0.15728
C	-2.10435	-3.79495	-2.54579
C	0.47146	-6.667	-0.51425
H	1.00438	-7.35398	0.17318
H	-0.48776	-7.1599	-0.77104
H	1.07188	-6.56291	-1.44101
C	-0.61792	-5.53177	1.45172
H	-0.83502	-4.5693	1.95702

H	-1.58516	-6.01588	1.20154
H	-0.06265	-6.17975	2.16186
Ni	-3.73763	-0.10568	-0.09176
N	-4.38634	-2.12999	1.92366
N	-6.29226	1.34258	0.159
N	-3.26181	-0.49987	2.81135
N	-6.29333	0.04369	-1.57533
C	-3.78915	-0.92781	1.60935
C	-5.50486	0.42728	-0.50914
C	-5.00252	-2.98615	0.89722
H	-4.99136	-2.33757	-0.00679
C	-0.95762	0.28059	3.26366
H	-0.59092	-0.45553	2.52152
H	-0.28645	1.16162	3.21168
H	-0.87311	-0.16115	4.27983
C	-4.22778	-2.44622	3.27143
C	-3.51883	-1.41314	3.83073
C	-7.52399	1.51748	-0.46679
C	-5.89823	1.89904	1.46554
H	-4.79593	1.76223	1.48165
C	-5.84616	-0.92969	-2.58571
H	-4.84462	-1.237	-2.21509
C	-2.39903	0.69437	2.94963
H	-2.42006	1.15332	1.93758
C	-7.52219	0.69932	-1.56889
C	-2.97096	1.68882	3.96619
H	-2.99137	1.26105	4.9914
H	-2.33474	2.59629	3.99411
H	-4.0013	1.99667	3.69992
C	-6.76809	-2.1543	-2.61118
H	-7.78451	-1.89678	-2.9786
H	-6.35227	-2.93102	-3.28458
H	-6.86826	-2.58968	-1.59671
C	-6.51787	1.05567	2.58897
H	-6.20479	-0.00275	2.48277
H	-6.18636	1.41695	3.58358
H	-7.62711	1.10636	2.55606
C	-4.12825	-4.21382	0.62187
H	-4.07158	-4.87887	1.51013
H	-4.5397	-4.80055	-0.22418
H	-3.10095	-3.9008	0.35022
C	-6.45416	-3.32009	1.25607
H	-7.04524	-2.39216	1.39729
H	-6.92211	-3.91317	0.44381
H	-6.52086	-3.92392	2.18618
C	-6.21126	3.39265	1.57083
H	-7.30431	3.59222	1.57519
H	-5.79965	3.79143	2.52014
H	-5.7435	3.94769	0.73376

C	-5.6713	-0.26178	-3.95331
H	-4.95054	0.57682	-3.87319
H	-5.26967	-0.99125	-4.68607
H	-6.63665	0.12292	-4.34673
H	4.83185	-5.08499	1.35472
H	-4.83179	5.08522	-1.35443
H	-2.69367	-4.10567	-3.42364
H	-4.62046	-3.3647	3.7189
H	-3.17586	-1.26241	4.85912
H	-8.29039	2.2039	-0.09395
H	-8.28367	0.54388	-2.33987
H	3.17557	1.25992	-4.85952
H	4.62048	3.36265	-3.72053
H	8.29115	-2.20241	0.09422
H	8.28415	-0.54148	2.33946
H	2.69388	4.10588	3.42374

Table S5. Cartesian coordinates for **3** at the B97D3/def2TZVPP level of theory.

Ni	0.36559	1.19841	0.3064
P	0.25043	0.47395	-1.89301
P	0.92628	1.28017	2.43483
O	2.58752	0.3974	-0.00956
N	-0.6371	3.788	-0.28114
N	1.50112	3.91017	-0.30087
C	2.95594	-0.15446	-1.20065
C	2.61587	0.49136	2.33803
C	1.9547	-0.20137	-2.18951
C	4.25424	-0.63392	-1.38223
C	3.27735	0.16213	1.14028
C	0.47925	3.04506	-0.09022
C	2.32051	-0.74668	-3.42465
H	1.57259	-0.79862	-4.21936
C	5.28985	-0.49585	-0.26118
C	3.60872	-1.22855	-3.64624
C	4.56482	-1.16977	-2.63697
H	5.57015	-1.54335	-2.8365
C	3.31028	0.24843	3.52682
H	2.82669	0.49471	4.47558
C	4.5915	-0.29862	3.51767
C	2.90986	3.5202	-0.34179
H	2.93282	2.53448	0.1318
C	5.22139	-0.57603	2.30822
H	6.23909	-0.96888	2.32031
C	4.58066	-0.33965	1.08761
C	-1.99424	3.24683	-0.19035
H	-1.83799	2.17807	0.01414
C	6.23994	-1.70307	-0.24549
H	5.6945	-2.63942	-0.05637
H	7.01124	-1.59176	0.5286

H	6.77355	-1.80119	-1.20042
C	-0.3232	5.09174	-0.60817
C	6.11034	0.78983	-0.5087
H	6.60533	0.75307	-1.49153
H	6.87754	0.91558	0.27094
H	5.45698	1.67355	-0.4859
C	1.02882	5.16901	-0.62673
C	-2.71314	3.38098	-1.52619
H	-2.8672	4.43645	-1.80261
H	-3.69715	2.89539	-1.47392
H	-2.13452	2.88535	-2.31977
C	3.36168	3.37349	-1.79242
H	2.71268	2.66231	-2.32407
H	4.3938	2.9985	-1.84285
H	3.32489	4.34058	-2.31916
C	-2.73779	3.86394	0.9851
H	-2.18583	3.68909	1.92009
H	-3.72793	3.4007	1.0894
H	-2.87584	4.94903	0.85121
C	3.77768	4.4683	0.47525
H	3.82857	5.47438	0.02968
H	4.80418	4.07692	0.52411
H	3.39533	4.55687	1.50223
H	1.68416	6.00751	-0.84035
H	-1.0764	5.85113	-0.79456
P	-0.25029	-0.47384	1.89295
P	-0.92621	-1.28003	-2.43488
O	-2.58718	-0.39687	0.00941
C	-2.95584	0.15449	1.20064
C	-2.6159	-0.49143	-2.33809
C	-1.95466	0.2012	2.18958
C	-4.25422	0.63366	1.38231
C	-3.27729	-0.16202	-1.14032
C	-2.32062	0.74598	3.42489
H	-1.57274	0.79774	4.21966
C	-5.28983	0.49606	0.26119
C	-3.60893	1.22751	3.64661
C	-4.56497	1.16894	2.63727
H	-5.57039	1.54222	2.8369
C	-3.31052	-0.24898	-3.52684
H	-2.82701	-0.49541	-4.4756
C	-4.59187	0.29777	-3.51765
C	-5.22165	0.57535	-2.3082
H	-6.23946	0.96795	-2.32025
C	-4.5807	0.33948	-1.08759
C	-6.23926	1.70384	0.24542
H	-5.69327	2.63988	0.05636
H	-7.01054	1.59299	-0.52875
H	-6.77288	1.80227	1.2003

C	-6.11107	-0.78914	0.5087
H	-6.60609	-0.75207	1.4915
H	-6.87832	-0.91447	-0.27096
H	-5.45822	-1.67323	0.48595
Ni	-0.36544	-1.19829	-0.30645
N	0.63713	-3.78789	0.28142
N	-1.5011	-3.91003	0.30036
C	-0.47914	-3.04492	0.09013
C	-2.90984	-3.51999	0.34092
H	-2.9326	-2.53423	-0.13257
C	1.99433	-3.24676	0.19117
H	1.83822	-2.17802	-0.01351
C	0.32308	-5.09163	0.6083
C	-1.02894	-5.16888	0.62635
C	2.71265	-3.38079	1.52734
H	2.86649	-4.43624	1.80396
H	3.69674	-2.8953	1.47539
H	2.13374	-2.88499	2.3206
C	-3.36204	-3.37342	1.79144
H	-2.71316	-2.66234	2.32334
H	-4.39415	-2.99838	1.84164
H	-3.32545	-4.34057	2.31808
C	2.73839	-3.86406	-0.98386
H	2.18699	-3.68914	-1.91916
H	3.72867	-3.401	-1.08763
H	2.87618	-4.94916	-0.84985
C	-3.77748	-4.46795	-0.47648
H	-3.82852	-5.47408	-0.03105
H	-4.80395	-4.07651	-0.52556
H	-3.39486	-4.55639	-1.50337
H	-1.68438	-6.00739	0.83968
H	1.0762	-5.85105	0.79495
H	-3.87201	1.64874	4.6191
H	3.87166	-1.65022	-4.61857
H	-5.11218	0.4896	-4.45819
H	5.11161	-0.49085	4.45822

4. References

[1] A. J. Sicard, R. T. Baker, *Org. Process Res. Dev.* **2020**, *24*, 2950–2952.
[2] T. Schaub, M. Backes, U. Radius, *Organometallics* **2006**, *25*, 4196–4206.
[3] W. Zhang, P.-C. Zhang, Y.-L. Li, H.-H. Wu, J. Zhang, *J. Am. Chem. Soc.* **2022**, *144*, 19627–19634.
[4] T. J. Hadlington, A. Kostenko, M. Driess, *Chem. – Eur. J.* **2021**, *27*, 2476–2482.
[5] M. Muhr, P. Heiß, M. Schütz, R. Bühler, C. Gemel, M. H. Linden, H. B. Linden, R. A. Fischer, *Dalton Trans.* **2021**, *50*, 9031–9036.
[6] G. M. Sheldrick, *Acta Crystallogr. Sect. C Struct. Chem.* **2015**, *71*, 3–8.
[7] A. L. Spek, *Acta Crystallogr. D Biol. Crystallogr.* **2009**, *65*, 148–155.
[8] M. J. Frisch, G. W. Trucks, H. B. Schlegel, G. E. Scuseria, M. A. Robb, J. R. Cheeseman, G. Scalmani, V. Barone, G. A. Petersson, H. Nakatsuji, X. Li, M. Caricato, A. V. Marenich, J. Bloino, B. G. Janesko, R. Gomperts, B. Mennucci, H. P. Hratchian, J. V. Ortiz, A. F. Izmaylov, J. L. Sonnenberg, Williams, F. Ding, F. Lipparini, F. Egidi, J. Goings, B. Peng, A. Petrone, T. Henderson, D. Ranasinghe, V. G. Zakrzewski, J. Gao, N. Rega, G. Zheng, W. Liang, M. Hada, M. Ehara, K. Toyota, R. Fukuda, J. Hasegawa, M. Ishida, T. Nakajima, Y. Honda, O. Kitao, H. Nakai, T. Vreven, K. Throssell, J. A. Montgomery Jr., J. E. Peralta, F. Ogliaro, M. J. Bearpark, J. J. Heyd, E. N. Brothers, K. N. Kudin, V. N. Staroverov, T. A. Keith, R. Kobayashi, J. Normand, K. Raghavachari, A. P. Rendell, J. C. Burant, S. S. Iyengar, J. Tomasi, M. Cossi, J. M. Millam, M. Klene, C. Adamo, R. Cammi, J. W. Ochterski, R. L. Martin, K. Morokuma, O. Farkas, J. B. Foresman, D. J. Fox, **2016**.
[9] A. D. Becke, *J. Chem. Phys.* **1997**, *107*, 8554–8560.
[10] F. Weigend, R. Ahlrichs, *Phys. Chem. Chem. Phys.* **2005**, *7*, 3297.
[11] J.-D. Chai, M. Head-Gordon, *J. Chem. Phys.* **2008**, *128*, 084106.
[12] S. Grimme, J. Antony, S. Ehrlich, H. Krieg, *J. Chem. Phys.* **2010**, *132*, 154104.
[13] T. Lu, F. Chen, *J. Comput. Chem.* **2012**, *33*, 580–592.
[14] R. F. W. Bader, *Atoms in Molecules: A Quantum Theory*, Clarendon Press, Oxford ; New York, **1990**.
[15] R. F. W. Bader, *J. Phys. Chem. A* **1998**, *102*, 7314–7323.
[16] E. D. Glendening, C. R. Landis, F. Weinhold, *J. Comput. Chem.* **2013**, *34*, 1429–1437.
